# Reduced *Pseudomonas aeruginosa* Cell Size Observed on Planktonic Cultures Grown in the International Space Station

**DOI:** 10.3390/microorganisms12020393

**Published:** 2024-02-16

**Authors:** Katherinne Herrera-Jordan, Pamela Pennington, Luis Zea

**Affiliations:** 1 Department of Biochemistry and Microbiology, Universidad del Valle de Guatemala, Guatemala City 01015, Guatemala; her16067@uvg.edu.gt; 2 Research Institute, Universidad del Valle de Guatemala, Guatemala City 01015, Guatemala; pamelap@uvg.edu.gt; 3 Aerospace Engineering Sciences Department, University of Colorado, Boulder, CO 80309, USA

**Keywords:** bacteria, space biofilms, ISS, spaceflight, cell diameter, length

## Abstract

Bacterial growth and behavior have been studied in microgravity in the past, but little focus has been directed to cell size despite its impact on a myriad of processes, including biofilm formation, which is impactful regarding crew health. To interrogate this characteristic, supernatant aliquots of *P. aeruginosa* cultured on different materials and media on board the International Space Station (ISS) as part of the Space Biofilms Project were analyzed. For that experiment, *P. aeruginosa* was grown in microgravity—with matching Earth controls—in modified artificial urine medium (mAUMg-high Pi) or LB Lennox supplemented with KNO_3_, and its formation of biofilms on six different materials was assessed. After one, two, and three days of incubation, the ISS crew terminated subsets of the experiment by fixation in paraformaldehyde, and aliquots of the supernatant were used for the planktonic cell size study presented here. The measurements were obtained post-flight through the use of phase contrast microscopy under oil immersion, a Moticam 10+ digital camera, and the FIJI image analysis program. Statistical comparisons were conducted to identify which treatments caused significant differences in cell dimensions using the Kruskal–Wallis and Dunn tests. There were statistically significant differences as a function of material present in the culture in both LBK and mAUMg-high Pi. Along with this, the data were also grouped by gravitational condition, media, and days of incubation. Comparison of planktonic cells cultured in microgravity showed reduced cell length (from 4% to 10% depending on the material) and diameter (from 1% to 10% depending on the material) with respect to their matching Earth controls, with the caveat that the cultures may have been at different points in their growth curve at a given time. In conclusion, smaller cells were observed on the cultures grown in microgravity, and cell size changed as a function of incubation time and the material upon which the culture grew. We describe these changes here and possible implications for human space travel in terms of crew health and potential applications.

## 1. Introduction

Bacterial cells can be found in different forms in nature. Broadly speaking, they can be separated into two forms: free-floating bacterial cells suspended in a medium, known as planktonic cells; and sessile bacterial cells, growing in either simple colonies or complex communities known as biofilms [1]. Biofilm-forming cells differ from their planktonic counterparts with respect to the genes that are transcribed [2]. Bacterial cells live in a planktonic state until they encounter favorable conditions to form biofilms. When biofilms reach the dispersion stage, the cells return to their planktonic state and the cycle starts again [3]. Specifically, there are three different processes in which cells from biofilms disperse or scatter: (1) detachment (when a large part of the biofilm detaches and therefore mass is lost in the colony), (2) erosion (when, one by one, the cells are separated from the biofilm by some external force), and (3) dispersion (when cells rapidly move from the biofilm by changing from non-motile to motile and leave the colony, leaving central holes in the biofilm) [3,4]. The first two forms are related to mechanical forces that cause this separation and can be sudden at any moment of the biofilm’s life cycle, while the third happens at the end of the biofilm life cycle. The morphological changes that happen while cells are part of a biofilm are maintained by the planktonic cells that come from detachment or dispersion of biofilms. Because of this, morphological changes in cells forming a biofilm are important to address in the context of this study, where cells come from a culture where planktonic and biofilm cells co-exist.

### 1.1. *Pseudomonas aeruginosa*

The ISS and future space stations and spacecraft, as closed environments, must maintain safe and sustainable air and water quality. Nevertheless, many microbial strains have been isolated inside and outside the ISS, with some of them being identified as of medical importance and possible hazards for astronauts [5,6,7]. One of these cells is *P. aeruginosa* [8], a bacterium normally studied for its pathogenic activity, its predominant persistence in clinical settings, and the potential danger posed by its resistant biofilms [8]. It is an opportunistic pathogen [9] and presents a special hazard on the ISS as it can express stress-resistant genes and can also increase expression of virulence factors [9]. On Earth, it is a major cause of infections and may even cause death for immunocompromised individuals [10], and it represents an important threat for astronauts. Although the importance is primarily due to its biofilms, planktonic cells of *P. aeruginosa* are equally important for humans’ health on the ISS and Earth.

The planktonic cells of the PA14 strain cultivated in LB medium under anaerobic conditions have been studied and demonstrated the presence of their exponential phase around five hours after their cultivation [11] and begins its stationary phase between eight and ten hours [12]. The replication time of *P. aeruginosa* cells is between 40 and 60 min [13] and the reversible fixation time of the cells starts at the moment of the culture. After two hours, the cells begin the non-reversible fixation process for the formation of biofilms, which reach the first maturation phase at 3 days. The second maturation phase ends at 6 days and dispersion phase begins from 9 to 12 days [4].

### 1.2. Planktonic Cells

Free-living cells are of medical and industrial importance as they represent a threat for industrial materials (such as stainless steel), mainly by a process known as Microbiologically Influenced Corrosion (MIC). During this process, microbes adhere to materials (metals in this case) and induce or accelerate corrosion reactions [14,15]. Planktonic cells on water systems (for example, stainless steel used in water recycling systems) may produce highly complex biofilms and may form corrosive byproducts [16]. Bacterial presence in medical devices, catheters, for example, may lead to nosocomial infections, including urinary tract infections [17].

This problem with planktonic cells and biofilms is something that affects not only materials on Earth but also in space. Problems within the ISS water processing assembly have been found to be caused by biofilms [6]; due to this, many characterization studies of the species present in these biofilms have been carried out [7] together with biofilm studies [18,19,20], and possible solutions have also been sought to reduce the threats that these present for current and future missions [19].

Due to the importance that planktonic cells have in the space environment, previous research explored their phenotypic characteristics to better understand their behavior. An example of this is a study published in 2017, where phenotypic changes in *Escherichia coli* cells potentially related to exposure to microgravity in the ISS were explored [20]. In that study, it was found that, on average, cells in space have only 37% the volume of their counterparts on Earth. In addition to this, more research has been carried out that mainly takes into account changes in cell length and diameter.

### 1.3. Cell Length and Diameter

Studies performed under simulated and actual microgravity [21], under low oxygen conditions [22], and low availability of nutrients [23] have shown reactive changes in cellular length and diameter. According to research on cell length, cell growth, and cell division, the minimum length of cells is the length of cells at a time zero (initial) of cell growth. In other words, in the lag phase of adaptation to the medium, they can be found at their minimum length until they begin to grow [12]. The cell then starts elongating and changing its morphology according to the environmental conditions, but, 20 min before cell division, the cell length must be approximately twice the minimum cell length throughout the growth [12]. Finally, the cell length should gradually decrease as the cells enter the stationary phase [24].

On the other hand, a limitation regarding oxygen has been shown to be related to a massive release of planktonic cells from biofilms in *P. aeruginosa* after approximately four hours of development [4], and this study presents an oxygen limitation for microbial cultures. Additionally, during the biofilm maturation process, the cells can disassociate from the biofilm matrix and resume planktonic life [25]. Also, anaerobic respiration has shown elongation effects on bacterial cells [26], which would make it equally interesting to study as it has been observed that some cells, including *P. aeruginosa*, adopt an anaerobic mode of growth during spaceflight [5]. In addition, it should be noted that this type of anaerobic growth also has effects on cell diameter [27].

The work presented here seeks to characterize the changes in cell length and diameter by comparing *P. aeruginosa* cultures under microgravity conditions on the ISS and under 1 g conditions. In addition to this variable with two conditions, the effects of the growth of biofilms on different materials with different characteristics, on the dimensions and morphology of the planktonic cells present in the culture, are studied. Finally, the effects of different culture media on the aforementioned changes regarding planktonic cells are also discussed.

### 1.4. Biofilms Project

The cells studied in the present manuscript are aliquots collected from the Space Biofilms Project, a NASA-funded investigation that took place on board the ISS [19,28,29]. In that study, *P. aeruginosa* biofilms were formed for one, two, and three days on six different materials.

## 2. Material and Methods

**Figure 1:**
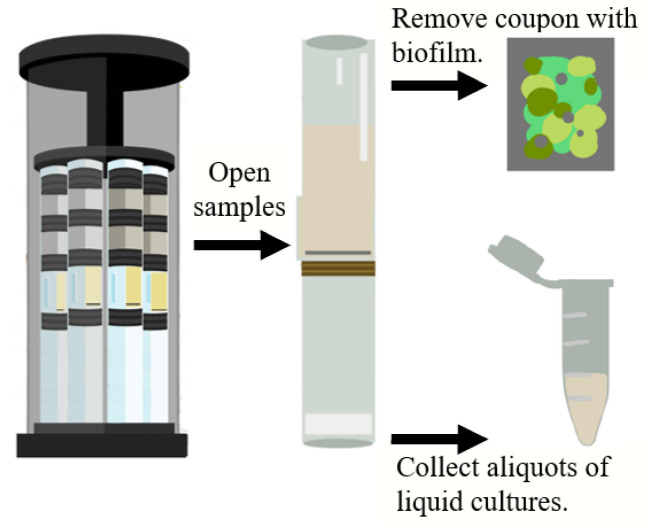
Aliquot sample collection process, showing the BioServe’s Group Activation Pack (GAP) loaded with eight fluid processing apparatuses (FPAs) (**left**), an opened FPA (**center**) [24,26], an example coupon as used by BioServe for their studies (**top right**), and a 0.5 mL centrifuge conical tube as the ones sent to the Universidad del Valle de Guatemala with suspended planktonic cells (**bottom right**).

In this mission, microgravity (μg) cultures of *P. aeruginosa* PA14 were grown in Luria-Bertani broth medium supplemented with potassium nitrate (KNO_3_) (here referred to as LBK) as the final electron acceptor and exposed to coupons of different materials: SS316, p- SS316, and Lubricated Impregnated Surface (LIS). Separately, cultures of *P. aeruginosa* PA14 were grown in modified Artificial Urine Medium supplemented with glucose and high phosphate (here referred to as mAUMg-high Pi) and exposed to coupons of cellulose membrane, silicone, and silicone-DLIP. As a control, equivalent cultures were performed in 1 g. Subsets of cultures were grown anaerobically for one, two, and three days. At the end of the incubation, the samples were fixed with a final 4% concentration of paraformaldehyde (PFA) (ACROS, Cat. No. 41678, New Jersey, NJ, USA). For each test condition (gravity, incubation time, media, and material), there were four replicates. After fixation, samples were stowed at 4 °C through return to Earth. Once back on Earth, the coupons with the biofilms were removed for separate analyses, and the liquid culture was aspirated. Aliquots of these liquid cultures were collected and shipped to Universidad del Valle de Guatemala (UVG) for this parallel and in-house-funded investigation (Figure 1). The exact method for this first part of the investigation can be found in Flores et al., 2022 [27,29], and more details can be found in Herrera-Jordan, 2022 [28].

### 2.1. Sample Preparation

**Figure 2:**
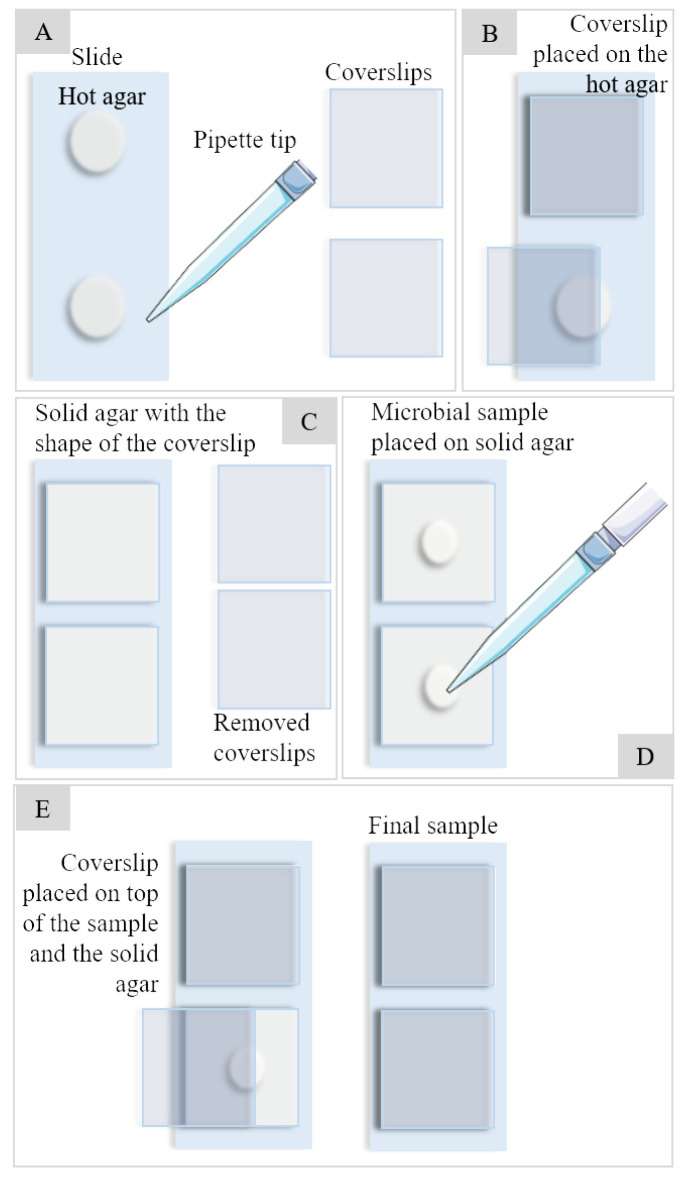
Sample preparation full process from agar bed preparation (**A**–**C**) to microbial sample pouring and fixation (**D**,**E**).

For the preparation of the samples (Figure 2), microscope slides (Marienfield Superior, Cat. No. 1000000) were used, and the steps are as presented in Figure 2A–E. Indeed, 400 μL of warm agarose at 4% (*w*/*v*) (Fisher Scientific, Product code 10367033) were placed in the first half of the slide and a coverslip was quickly placed. The process was repeated on the second half of the slide and allowed to cool. After cooling and having a solid agarose bed, the coverslip was removed and 3 μL of the sample was added to the center of the box formed by the coverslip in the agarose. The coverslip was replaced to protect the sample and it was observed under the microscope. No type of staining was used; only crude samples were used.

### 2.2. Microscopy and Photo-Sampling

**Figure 3:**
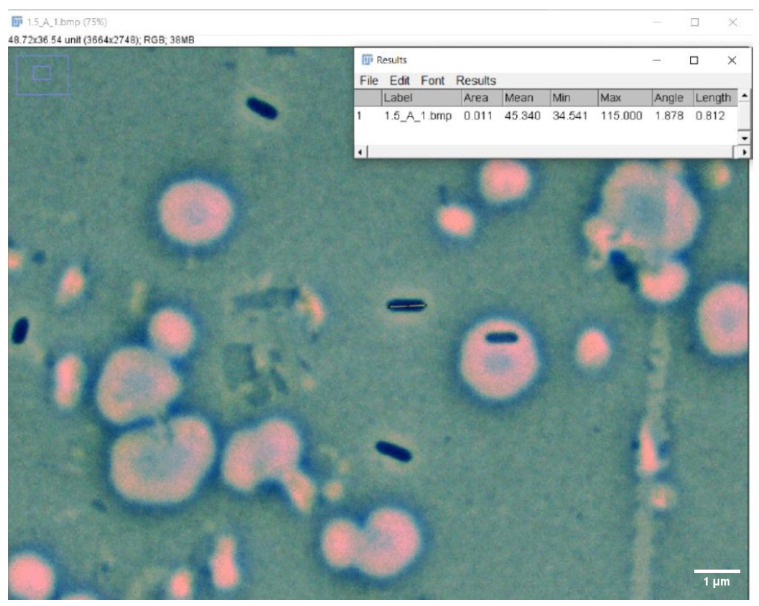
Cell measuring process. The figure shows the FIJI software and how cell length was measured. For cell diameter measurements, the same tool and process was used but the line was drawn horizontally.

A Fisher Scientific Micromaster phase contrast microscope (Fisher Scientific, Waltham, MA, USA) was used with 20×, 40×, and 100× AMSCOPE PHP objectives with a Halogen light. The immersion oil used was Cargille Type A, formula code 1248, standardized at 23 °C. Following the methodology of Luppens and Steinberger of samples recommended for statistical analysis for the measurement of cell diameter and length, 100 cells were measured for each variable [30,31]. For this, a Moticam 10+ (Motic Xiamen, Fujian, China) was used, attached to the left ocular of the microscope and never moved during the whole sampling process. To measure the cells’ length and diameter, the program FIJI ImageJ of 2017 was used (Figure 3), calibrated with the calibration circle of the Moticam 10+ camera (Motic, Xiamen, China). All the measurements resulted from this step and used in the present paper can be found in the supplementary material, Table S1.

### 2.3. Statistical Analysis

**Figure 4:**
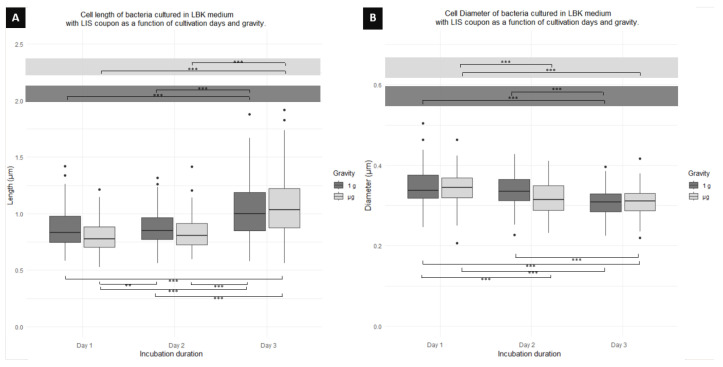
Boxplot diagrams of planktonic cells of *P. aeruginosa* co-cultured using an LIS coupon showing the (**A**) length and (**B**) diameter differences as function of the days of fixation and gravity conditions. Horizontal lines represent significant differences with *p*-values ** < 0.001, *** < 0.0001; n = 300 for μg and 300 for 1 g.

**Figure 5:**
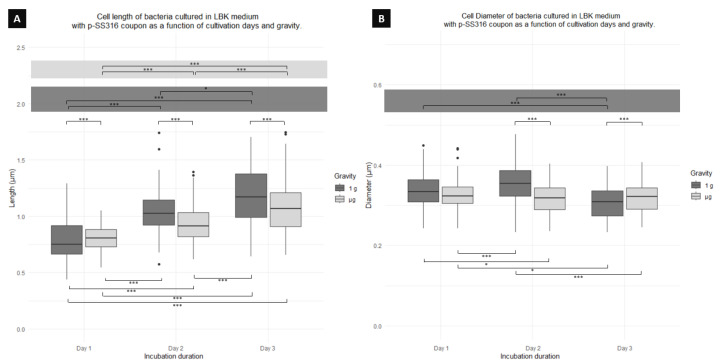
Boxplot diagrams of planktonic cells of *P. aeruginosa* co-cultured using a p-SS316 coupon showing the (**A**) length and (**B**) diameter differences as function of the days of fixation and gravity conditions. Horizontal lines represent significant differences with *p*-values * < 0.05, *** < 0.0001; n = 300 for μg and 300 for 1 g.

**Figure 6:**
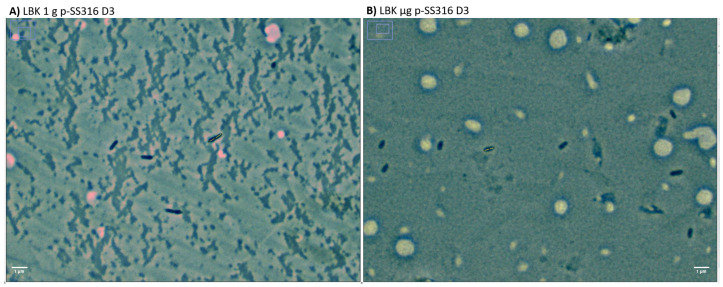
Representative images of planktonic cells of *P. aeruginosa* from day 1 co-cultured using a p-SS316 coupon showing the (**A**) cells cultured under 1 g and (**B**) cells cultured under μg. It is possible to appreciate how cells cultured under 1 g are larger than those cultured under μg.

**Figure 7:**
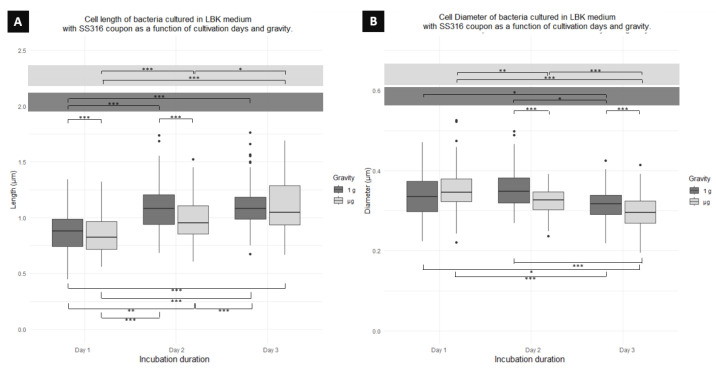
Boxplot diagrams of planktonic cells of *P. aeruginosa* co-cultured using an SS316 coupon showing the (**A**) length and (**B**) diameter differences as function of the days of fixation and gravity conditions. Horizontal lines represent significant differences with *p*-values * < 0.05, ** < 0.001, *** < 0.0001; n = 300 for μg and 300 for 1 g.

**Figure 8:**
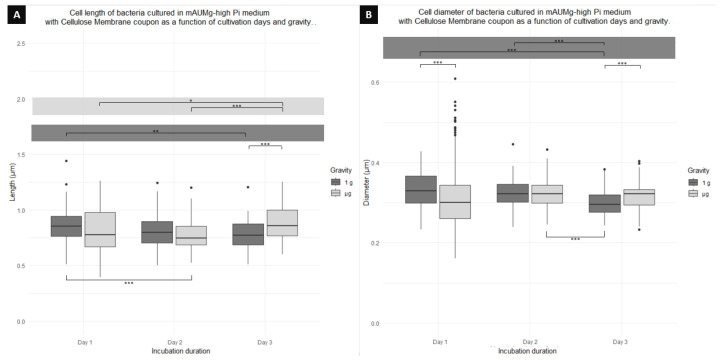
Boxplot diagrams of planktonic cells of *P. aeruginosa* co-cultured using a cellulose membrane coupon showing the (**A**) length and (**B**) diameter differences as function of the days of fixation and gravity conditions. Horizontal lines represent significant differences with *p*-values * < 0.05, ** < 0.001, *** < 0.0001; n = 300 for μg and 300 for 1 g.

**Figure 9:**
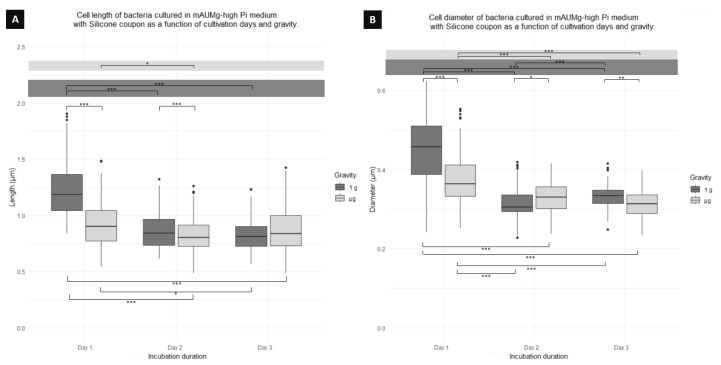
Boxplot diagrams of planktonic cells of *P. aeruginosa* co-cultured using a silicone coupon showing the (**A**) length and (**B**) diameter differences as function of the days of fixation and gravity conditions. Horizontal lines represent significant differences with *p*-values * < 0.05, ** < 0.001, *** < 0.0001; n = 300 for μg and 300 for 1 g.

**Figure 10:**
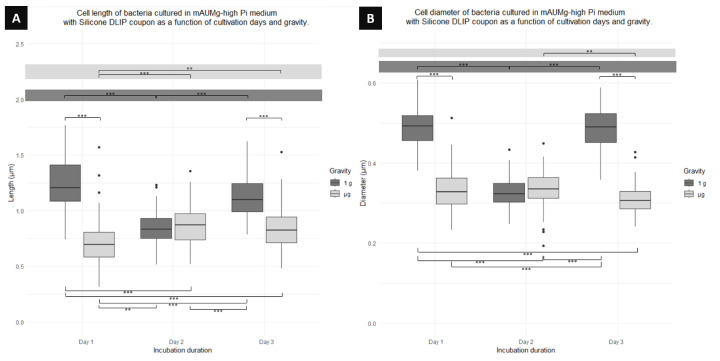
Boxplot diagrams of planktonic cells of *P. aeruginosa* co-cultured using a Silicone DLIP coupon showing the (**A**) length and (**B**) diameter differences as function of the days of fixation and gravity conditions. Horizontal lines represent significant differences with *p*-values ** < 0.001, *** < 0.0001; n = 300 for μg and 300 for 1 g.

Normality and homogeneity of samples were measured for each group of data and the whole dataset. Each group was assessed by a Levene test. A Kruskall–Wallis test was carried out to compare means for all growth conditions (incubation duration, material, and gravitational conditions). Finally, a Dunn test was conducted. Significant differences were calculated with the Dunn’s test and are shown with *p*-values * < 0.05, ** < 0.001, *** < 0.0001. Boxplot diagrams were generated for a visual representation of cell characteristics through days 1–3 for each material used. Lines at the top of Figures 3–10 connect days with significant differences and present the *p*-value above the line.

## 3. Results

### 3.1. LBK Results

#### 3.1.1. Cell Length and Diameter Changes as a Function of Incubation Time and Gravity, Co-Cultured with LIS Coupon

Figure 4A shows a significant difference in the cell length of those cultivated in 1 g, with a tendency of elongating with time. Cells on day 1 and 2 are significantly shorter than cells of day 3, with a difference of 17% between day 1 and 3. Similarly, cells cultured in μg also showed an elongation with time. On days 1 and 2, there is a significant difference when compared to cells on day 3, with a difference in length from day 1 to 3 of 26%. When comparing cell length of cultures of the same day but different gravity conditions, no significant difference was found for any of the 3 days (Table 1). On the other hand, when comparing cell lengths between days and gravity conditions, a significant difference is shown between day 1 in 1 g and day 3 in μg, as well as a significant difference between day 1 in μg and day 2 in 1 g; day 2 in μg and day 3 in 1 g; day 1 in μg and day 3 in 1 g; and, finally, between day 2 in 1 g and day 3 in μg.

Figure 4B displays a decrease in the cell diameter of cells with time, those cultured in 1 g on days 1 and 2 being significantly wider (31% and 29%, respectively) than the cells of day 3. Similarly, cells cultured in μg showed a diameter decrease with time, the cells on days 2 and 3 being significantly shorter in diameter when compared to cells on day 1. Cells on day 1 showed a diameter 10% greater than those on day 3. No significant difference in cell diameter is observed between cells in 1 g and μg for any of the fixation days (Table 1). A significant difference was found when comparing the diameters of cells from day 1 in 1 g with cells from day 2 in μg and cells from day 3 in μg. Similarly, a significant difference in cell diameter was found for the culture on day 1 in μg when compared to day 3 in 1 g. Finally, a significant difference was found when comparing cells from day 2 in 1 g with cells from day 3 in μg.

#### 3.1.2. Cell Length and Diameter Changes as a Function of Incubation Time and Gravity, Co-Cultured with p-SS316 Coupon

Figure 5A shows a significant difference in cell length between cells cultured in 1 g and μg for all days (days 1, 2, and 3). Table 1 shows that cells of 1 g are significantly shorter (2%) than those of μg for day 1, cells of μg shorter (10%) than those of 1 g for day 2, and cells of μg shorter than 1 g (9%) for day 3 (Figure 6). For cells cultured in 1 g, a tendency of an increase in length with time is observed, with a significant difference between cell length on day 1 and day 2, day 1 and day 3, and day 2 and day 3, the difference between day 1 and 3 being the most notable (33%). Similarly, for cultures in μg, an increase in length with time was also found, with significant differences between cells on day 1 and day 2, day 1 and day 3, and day 2 and 3, the difference between day 1 and 3 being the most extreme (25%). Finally, when comparing between days and gravity condition, a significant difference was found in the cell length of cultures from day 1 in 1g and day 3 in μg; day 1 in μg and day 3 in 1 g; day 1 in 1g and day 2 in μg; day 1 in μg and day 2 in 1g; and between day 2 in μg and day 1 in 1g.

Figure 5B shows significant differences in cell diameter between cultures comparing 1 g and μg for day 2 and day 3. Table 1 shows that cells cultured under μg for day 2 have the smallest diameter, with a difference of 10% when compared to 1 g. The table also shows that cells under μg showed the smallest diameter for day 3 with a difference of 9% when compared to 1 g. For cultures in 1 g, cells were significantly wider between days 1 (1%) and 2 (6%) compared to day 3. There were no significant differences between culture days for cells cultured in μg. Finally, between days and gravity conditions, significant differences were found between cells from day 1 in 1 g and day 2 in μg, between day 1 in μg and day 2 in 1 g, and day 1 in μg and day 3 in 1 g; and similarly, significant differences were found between day 2 in 1 g and day 3 in μg.

#### 3.1.3. Cell Length and Diameter Changes as a Function of Incubation Time and Gravity, Co-Cultured with SS316 Coupon

Figure 7A indicates a significant difference between cells cultured in 1 g and μg for days 1 and 2, with 1g cells being longer by 4% than μg cells, and cells on day 2 being longer 10% than μg cells (Table 1). On day 3, there was no significant difference between gravity conditions. Additionally, among the cells cultured in 1g, a significant difference was found in cell length between day 1 and day 2, and day 1 and day 3. Similarly, for cells cultured in μg, a significant difference was found between cells on day 1 and day 2, day 1 and day 3, and day 2 and day 3, indicating a cellular elongation across days. Finally, significant differences were observed between days and gravity conditions. The length of cells on day 1 in 1 g is significantly different from 1 g cells on day 3 and μg cells on day 2. μg cells on day 1 are significantly different from 1g cells on day 2 and day 3. Cells on day 2 in μg are significantly different from cells on day 3 in 1g.

Figure 7B also shows differences in cell diameter. When comparing cells from the same day but different gravitational conditions, cells on day 2 in μg have a significantly shorter diameter (9% Table 1) than cells from the same day cultured in 1 g. Similarly, cells on day 3 in μg have a significantly smaller diameter (1%) than cells cultured in 1 g. When comparing between culture days for cells cultured in 1 g, a significant difference was found between cells on day 1 and 3, and day 2 and 3, but no difference between cells on day 1 and 2. For cells in μg, a significant difference was found between cells on day 1 and 2, 1 and 3, and 2 and 3. Finally, comparing between different culture days and gravity conditions, a significant difference was found between cells on day 1 in 1g and day 3 in μg, as well as between cells on day 1 in μg and cells on day 3 in 1 g, and cells on day 2 in 1 g and cells on day 3 in μg.

### 3.2. mAUMg-High Pi Results

#### 3.2.1. Cell Length and Diameter Changes as a Function of Incubation Time and Gravity, Co-Cultured with Cellulose Membrane Coupon

Figure 8A indicates a significant difference between cells cultured in μg and 1 g only on day 3, where 1 g cells are 13% shorter than μg cells (Table 2). On the other hand, when comparing cultures performed in 1g, a significant difference is observed between day 1 and day 3. Similarly, in cultures performed in μg over days, there is a significant difference only between cells on day 1 and day 3, and day 2 and day 3. Finally, comparing different gravity conditions and culture days, a significant change is detected only for day 1 in 1 g and day 2 for μg.

Figure 8B also shows differences in cell diameter. In this case, we find a significant difference depending on the day in function of gravity for days 1 and 3, where there is a difference in the diameter of cells cultured in 1 g and μg. Cells cultured in μg on day 1 have a diameter 12% shorter than cells in 1 g. On day 3, the cell diameter of μg cultures is again smaller than that of 1 g by 6%. For this coupon, no significant difference was found between the culture days for cells grown in μg. However, cells cultured in 1 g showed a significant difference when comparing day 1 with day 3, and day 2 with day 3. The greater difference is between day 1 and 3, the cells of day 1 being wider than those of day 3 by 28%. Finally, comparing between days and gravity conditions, we found only a significant difference between day 2 in μg and day 3 in 1 g.

#### 3.2.2. Cell Length and Diameter Changes as a Function of Incubation Time and Gravity, Co-Cultured with Silicone Coupon

Comparing the significant difference in cell length across days, depending on gravity, Figure 9A, shows a significant difference between 1 g and μg cells on day 1, where 1 g cells are 25% longer than μg cells. Similarly, on day 2, a significant difference between 1 g cells occurs, again having 1 g cells being longer than μg cells, this time by 4% (Table 2). On the other hand, when comparing only among cells cultured in 1 g over days, a significant difference is found between cells on day 1 and day 2, with the cells from day 1 being longer than day 2 cells by 30% and cells on day 1 and day 3 with day 1 cells being 33% longer than day 3 cells. When comparing cells cultured in μg, only a significant difference between day 1 and day 2 is found. Finally, comparing between gravity conditions and days, a significant difference is found between day 1 in 1 g and day 2 in μg; day 1 in 1g and day 3 in μg; and between day 1 in μg and day 3 in 1 g.

Figure 9B also shows differences in cell diameter. Comparing between 1 g and μg for each day, there is a significant difference in cell diameter on day 1, where 1g cells have a diameter 15% larger than that of the μg culture. Likewise, there is a difference between cultures on day 2, but, this time, μg cells have a diameter 5% larger than 1 g cells. Similarly, day 3 also shows a difference where, again, 1 g cells have a diameter 6% larger than μg cells. On the other hand, when comparing cells cultured in 1 g over days, a difference is found between day 1 and day 2; day 1 and day 3; and day 2 and day 3. The cells of day 1 were 30% wider than those of day 2, and 26% wider than those of day 3. Cells cultured in μg also showed significant differences across days, displaying a difference between day 1 and day 2 and day 1 and day 3. Finally, comparing between gravity conditions and days, a difference is found between day 1 in 1g compared to day 2 in μg and day 3 in μg. Similarly, a difference is found between day 1 in μg when compared to day 2 in 1 g and day 3 in 1 g.

#### 3.2.3. Cell Length and Diameter Changes as a Function of Incubation Time and Gravity, Co-Cultured with Silicone-DLIP Coupon

Comparing the cell length of the cultures presented in Figure 10A, a significant difference in cell growth between cells grown in μg and 1 g is apparent. On day 1, 1 g cells are 42% longer than μg cells (Table 2). Similarly, on day 3, 1 g cells are 37% longer than μg cells. On the other hand, when comparing cell growth over days for cells cultured in 1 g, there is a significant difference between cells on day 1 and day 2 (cells of day 1 are 31% longer than cells of day 2), and between day 2 and day 3 (with day 3 being 25% longer than day 2). However, observing the growth of cells cultured in μg over days, a significant difference is noted between day 1 and 2, where day 2 has longer cells, and between day 1 and 3, where day 3 has longer cells compared to day 1. Finally, comparing between gravity conditions and days, a length difference is observed between day 1 in 1g compared to day 2 in μg and day 3 in μg. Similarly, day 1 cells in μg show a significant difference compared to day 2 in 1 g and day 3 in 1 g. Day 2 in μg also shows a significant difference in length compared to day 3 in 1 g.

The same Figure 10B shows differences in cell diameter for this culture. It was found that, over days and depending on gravity conditions, there is a significant difference for days 1 and 2. On day 1, cells in 1 g have a diameter 32% larger than cells in μg. Similarly, on day 3, cells in 1 g have a diameter 37% larger than cells cultured in μg for the same day. When comparing cells cultured in 1 g over days, day 1 shows the largest diameter with cells 33% wider from day 2. At the same time, cells from day 2 are significantly different from day 3, where cells of day 3 are also 33% wider than cells of day 2, but there is no difference between day 1 and 3. Likewise, when comparing cells cultured in μg, it is found that there is only a significant difference between cells on day 2 and 3, with day 3 cells having a smaller diameter. Finally, comparing between gravity conditions and days, there is a diameter difference between day 1 in 1 g and days 2 and 3 in μg. Day 1 in μg also showed a significant difference with day 3 in 1 g, and day 2 in μg demonstrated a difference with day 3 in 1 g.

## 4. Discussion

### 4.1. LBK Cultures

The cells used during this study may have been between the non-reversible fixation processes for the formation of biofilms, just reaching the first maturation phase on the third day. This suggests that the studied planktonic cells did not come from the process of dispersion of biofilms but may have originated from a detachment. This is supported by the literature as it has been reported that *P. aeruginosa* cells can grow anaerobically through the use of nitrate or nitrite [32], but this results in elongation of the cells [31] and a massive release of planktonic cells from biofilms of *P. aeruginosa* after approximately 4 h of development [24]. As the present paper presents an oxygen limitation, this could explain the elongation of *P. aeruginosa* cells cultured under 1 g.

Something interesting that is observed in the results is that the three materials start with a minimum length in their planktonic cells, both under 1 g and in μg, and experience elongation by day 2 and 3. This may indicate that, upon reaching the first day, the original planktonic cells that were in the process of division had already reached their stationary phase/biofilm maturation phase, mainly for the cultures created with LIS, explaining the short length. Additionally, cell elongation with thickness maintained has been reported with nutritional imbalances in carbon or nitrogen, low carbon availability, or even a higher nutrient diffusion pathway [15].

Regarding the cell diameter, the width of *P. aeruginosa* cells is reported to remain constant regardless of cell length. However, the cells of the present study show a different behavior: when the cell length is short, the diameter is large, and, when the length is greater, the width is small. In μg cultures (Figure 4, Figure 5, and Figure 7), a similar trend was observed. A possible explanation of why the diameter decreases when the length increases agrees with the availability of nutrients in the medium. When the availability of nutrients is low, the cell must begin to increase its volume (radius length/width) in order to obtain a greater amount of nutrients. On the other hand, when the cell finds nutrients, it must decrease it [33]. This would indicate that, on the first day, planktonic cells compete with sessile cells for nutrients, so they must increase their volume. As the biofilm matures, it leaves more nutrients available for the planktonic cells, and these can finally decrease their volume. When comparing cells from μg and 1 g in the same conditions (Table 1), the differences begin on day 2, showing cells in 1 g with larger diameter than those cultured in μg. This follows the principle of homogeneity of nutrients in the culture medium since, in μg, the lack of force exerted by gravity prevents nutrients from settling and allows them to be distributed more homogeneously in a medium [34].

In the present paper, we report that cells in microgravity tend to have a shorter length (4–10% shorter depending on the day and the material) and a smaller diameter (1–10% smaller depending on the day and the material) (Table 1). It is important to note that similar behavior has been reported [20], where it was found that *E. coli* cells on space cultures were 59% the length of cells on Earth and 83% the diameter of cells on Earth controls. The paper [20] uses the same culture medium (LBK) and mentions a probable role of nutrient concentration on the resulting smaller length and diameter of cultured cells in μg compared to those of 1 g. In other studies using *P. aeruginosa* as a model organism, cellular elongation of 20–25% has been reported during biofilm formation compared to control cells [31]. In this case, the cells show ∼33% elongation compared to cells from day 2 and 3 for the silicone and silicone-DLIP culture, which further supports the hypothesis of the elongation being a result of the dispersion of cells from biofilms.

Finally, cell diameter changes are noteworthy when using LBK because it behaves inversely compared to mAUMg-high Pi, as expressed in the next subsection. Here, cells change their diameter inversely to the cells’ length, meaning that, the day on which the cell has the shortest length, it has the largest diameter. This may be due to the fact that the cell is in its initial state on day 1 and its diameter decreases as the cells lengthen to finally form the septum. Another explanation of why the diameter increases when the length decreases could be the availability of nutrients in the medium. This would indicate that, on the first day, planktonic cells compete with sessile cells for nutrients, so they must increase their volume. As the days go by and the biofilm matures, it leaves more nutrients available for the planktonic cells, and these can finally decrease their volume.

### 4.2. mAUM-High Pi Cultures

The most important thing to state from the beginning is that the mAUMg-high Pi culture is an anaerobic culture with respiration activated by nitrate. This is important as this nitrate-activated respiration induces greater formation of biofilm [34], quicker growth and time to reach the stationary phase, cell elongation as a consequence of defective cell division, and weaker biofilms [25]. With our results, it is interesting to note that the cells measured for silicone and silicone-DLIP, on day 1 under 1 g, are larger than the average of the rest of the cells (Figure 9 and Figure 10). These large measurements could result from a detachment of cells from the biofilm to their planktonic life form through a process of dispersion of active type or a detachment process. However, it would be necessary to perform flagella staining to ensure whether this hypothesis is correct or not. It would be interesting to review the matrix of the biofilms on day 1 of silicone and silicone-DLIP to see if there is detachment of cells that go from sessile life to free life due to defective elongation and weak attachment to the biofilm.

An important thing to note is that, conversely to the results of cells cultured with LBK, cells cultured with mAUMg-high Pi showed that an increase in cell length results in increased cell diameter. This growth in cell volume may be related to the need to access nutrients, or support the idea of possible failures in the formation of the separation septum at the moment of beginning its replication process and, therefore, failed cell reproduction. For the cells cultured under μg conditions (Figure 8, Figure 9 and Figure 10), such differences were not observed, with a tendency to a homogeneous cell length over time. This may indicate that enough nutrients were available for cells under microgravity compared with the LBK medium. This is true as the amount of nutrients for this culture medium (mAUMg-high Pi) is higher compared to LBK.

In another study that used *Psedomonas aeruginosa* and explored the biofilm formation of this cell under microgravity conditions, biofilms grown in the International Space Station show a dense mat-like structure that forms a canopy over the columns. In contrast to this, cells cultured under normal gravity showed uniformly dense structures. Either way, both biofilms show strong and dense structures, which could explain why planktonic cells of the cultures conducted with cellulose membrane showed little difference, with the exception of day 3 for microgravity planktonic cells, which could be explained by a dispersion process caused by the stage of the biofilm [34]. It is also important to note that, in the same study, no differences between spaceflight and normal gravity were reported between planktonic cells when the phosphate concentration is high, as it is in our culture medium mAUMg-high Pi. This is consistent with our results that report no difference between planktonic cells cultured under microgravity and normal gravity conditions on day 1 and 2 but a small difference (*p*-value < 0.05 *) on day 3.

In a similar way, cells cultured with silicone-DLIP, a material designed to inhibit cell growth, demonstrated shorter cells on the first day, followed by a significant enlargement of these cells on day 2, and shortening by the third day. This may indicate that the cells are being kept from generating biofilms, and a planktonic life is being favored. The planktonic cell dispersion in this case appears to be the normal dispersion method of biofilms as part of their life cycle. Planktonic cells from a dispersal process overall show a similar physiology to that of planktonic cells derived from biofilm [35,36]; however, they present a longer lag phase of approximately 3 extra hours than that of the original planktonic cells [37].

## 5. Conclusions

The existence of a statistically significant difference in the diameter and length of planktonic cells of *P. aeruginosa* cultivated in microgravity in different culture media and materials of interest was determined. For the LBK culture medium, and the LIS, SS316, and p-SS316 materials, it was determined that the cells’ length increased from day 1 to day 3 for all the materials and gravitational conditions. In addition, for the LIS and SS316 materials, the cells with the largest diameter were found on the first day, indicating that the cells became longer and thinner with time, except in the case of pSS316 in μg, where the cells became longer without a change in diameter. LIS was the only material in which there was no detectable difference in length or diameter according to gravitational conditions. This suggests that this material could be effective in reducing the effects of μg on cellular growth.

Regarding the culture medium mAUMg-high Pi and the cellulose membrane, silicone, and silicone-DLIP materials, it was found that the length of the cell in the cellulose membrane does not vary significantly. However, for silicone and silicone-DLIP, the day with the greatest cell length turns out to be day 1. For silicone-DLIP, this length decreases drastically on day 2 and increases again on day 3. Regarding the diameter, it was possible to determine that the diameter was also the largest on day 1 for silicone and silicone-DLIP, in addition to increasing again on day 3 for silicone-DLIP.

The study showed that *P. aeruginosa* cells grown for 24 h under microgravity environments tend to be shorter compared to cells grown at 1 g, except when grown in the presence of cellulose membrane, LIS, and pSS316. Analysis of these materials may provide insight into the characteristics required to inhibit the effects of microgravity on bacterial growth in support of space exploration strategies. Future studies should assess cell density at each time point to determine the effects of microgravity and the materials under study on growth rates.

## Figures and Tables

**Table 1:** Cellular length and diameter differences between 1 g and μg cultures using LBK and LIS, SS316, and p-SS316 coupons as function of time. Significant differences with *p*-values *** < 0.0001 on Dunn’s test. Not significant difference (NS).

Cell Length Comparison						
	**Day 1**		**Day 2**		**Day 3**	
**Material Present in Culture**	**Shortest Length**	**Difference (%)**	**Shortest Length**	**Difference (%)**	**Shortest Length**	**Difference (%)**
**LIS**	NS	NS	NS	NS	NS	NS
**SS316**	μg ***	4	μg ***	10	NS	NS
**p-SS316**	1 g ***	2	μg ***	10	μg ***	9
**Cell Diameter Comparison**						
	**Day 1**		**Day 2**		**Day 3**	
**Material Present in Culture**	**Shortest Diameter**	**Difference (%)**	**Shortest Diameter**	**Difference (%)**	**Shortest Diameter**	**Difference (%)**
**LIS**	NS	NS	NS	NS	NS	NS
**SS316**	NS	NS	μg ***	9	μg ***	1
**p-SS316**	NS	NS	μg ***	10	1 g ***	4

**Table 2:** Cellular length and diameter differences between 1 g and μg cultures using mAUMg-high Pi and cellulose membrane, silicone, and silicone-DLIP coupons as function of time. Significant differences with *p*-values * < 0.05, ** < 0.001, *** < 0.0001 on Dunn’s test. Not significant difference (NS).

Cell Length Comparison						
	**Day 1**		**Day 2**		**Day 3**	
**Material Present in Culture**	**Shortest Length**	**Difference (%)**	**Shortest Length**	**Difference (%)**	**Shortest Length**	**Difference (%)**
**Cell Memb**	NS	NS	NS	NS	1 g ***	13
**Silicone**	μg ***	25	μg ***	4	NS	NS
**Silicone-DLIP**	1 g ***	42	NS	NS	μg ***	25
**Cell Diameter Comparison**						
	**Day 1**		**Day 2**		**Day 3**	
**Material Present in Culture**	**Shortest Diameter**	**Difference (%)**	**Shortest Diameter**	**Difference (%)**	**Shortest Diameter**	**Difference (%)**
**Cell Memb**	μg ***	12	NS	NS	1 g *	6
**Silicone**	μg ***	15	1 g *	5	μg **	6
**Silicone-DLIP**	μg ***	32	NS	NS	μg ***	37

## Data Availability

Data are contained within the article.

## Supplementary Materials

The following supporting information can be downloaded at: https://www.mdpi.com/article/10.3390/microorganisms12020393/s1, Table S1: Cell length and diameter measurements according to gravity, day of fixation, replicate, medium and coupon material.
