# Supplementary material for: Reduced Pseudomonas aeruginosa Cell Size Observed on Planktonic Cultures Grown in the International Space Station
Source: Microorganisms. 2024 Feb 16;12(2):393. doi: 10.3390/microorganisms12020393 (PMC10892763; doi:10.3390/microorganisms12020393)
Supplement: Supplementary file 1 [file microorganisms-12-00393-s001.zip › microorganisms-2810989-supplementary.pdf]

Table S1: Cell length and diameter measurements according to gravity, day of fixation, replicate, medium and coupon material.

Sample	Replicate	Day	Gravity	Medium	Material	Length (µm)	Diameter (µm)	Cell ID
G1.1	1	1	1 g	LBK	SS316	0.929	0.388	1
G1.1	1	1	1 g	LBK	SS316	0.708	0.271	2
G1.1	1	1	1 g	LBK	SS316	0.879	0.267	3
G1.1	1	1	1 g	LBK	SS316	0.938	0.287	4
G1.1	1	1	1 g	LBK	SS316	0.701	0.354	5
G1.1	1	1	1 g	LBK	SS316	0.681	0.296	6
G1.1	1	1	1 g	LBK	SS316	0.629	0.373	7
G1.1	1	1	1 g	LBK	SS316	0.875	0.298	8
G1.1	1	1	1 g	LBK	SS316	0.648	0.290	9
G1.1	1	1	1 g	LBK	SS316	0.807	0.313	10
G1.1	1	1	1 g	LBK	SS316	0.828	0.283	11
G1.1	1	1	1 g	LBK	SS316	1.002	0.342	12
G1.1	1	1	1 g	LBK	SS316	1.236	0.255	13
G1.1	1	1	1 g	LBK	SS316	0.846	0.392	14
G1.1	1	1	1 g	LBK	SS316	0.889	0.287	15
G1.1	1	1	1 g	LBK	SS316	0.955	0.403	16
G1.1	1	1	1 g	LBK	SS316	0.933	0.375	17
G1.1	1	1	1 g	LBK	SS316	0.706	0.358	18
G1.1	1	1	1 g	LBK	SS316	0.846	0.376	19
G1.1	1	1	1 g	LBK	SS316	0.976	0.425	20
G1.1	1	1	1 g	LBK	SS316	0.765	0.363	21
G1.1	1	1	1 g	LBK	SS316	0.909	0.300	22
G1.1	1	1	1 g	LBK	SS316	0.877	0.351	23
G1.1	1	1	1 g	LBK	SS316	1.083	0.331	24
G1.1	1	1	1 g	LBK	SS316	0.909	0.309	25
G1.1	2	1	1 g	LBK	SS316	0.954	0.366	1
G1.1	2	1	1 g	LBK	SS316	0.661	0.362	2
G1.1	2	1	1 g	LBK	SS316	0.565	0.329	3
G1.1	2	1	1 g	LBK	SS316	0.779	0.300	4
G1.1	2	1	1 g	LBK	SS316	0.589	0.234	5
G1.1	2	1	1 g	LBK	SS316	1.085	0.376	6
G1.1	2	1	1 g	LBK	SS316	0.888	0.331	7
G1.1	2	1	1 g	LBK	SS316	0.841	0.249	8
G1.1	2	1	1 g	LBK	SS316	0.884	0.289	9
G1.1	2	1	1 g	LBK	SS316	0.690	0.351	10
G1.1	2	1	1 g	LBK	SS316	1.174	0.349	11
G1.1	2	1	1 g	LBK	SS316	0.867	0.380	12
G1.1	2	1	1 g	LBK	SS316	1.125	0.376	13
G1.1	2	1	1 g	LBK	SS316	0.614	0.283	14
G1.1	2	1	1 g	LBK	SS316	0.447	0.308	15
G1.1	2	1	1 g	LBK	SS316	0.951	0.316	16
G1.1	2	1	1 g	LBK	SS316	0.745	0.362	17
G1.1	2	1	1 g	LBK	SS316	0.907	0.376	18
G1.1	2	1	1 g	LBK	SS316	0.751	0.376	19
G1.1	2	1	1 g	LBK	SS316	0.471	0.309	20
G1.1	2	1	1 g	LBK	SS316	0.722	0.304	21
G1.1	2	1	1 g	LBK	SS316	0.962	0.276	22

G1.1	2	1	1 g	LBK	SS316	0.578	0.268	23
G1.1	2	1	1 g	LBK	SS316	0.860	0.436	24
G1.1	2	1	1 g	LBK	SS316	0.947	0.309	25
G1.1	3	1	1 g	LBK	SS316	1.066	0.233	1
G1.1	3	1	1 g	LBK	SS316	0.726	0.309	2
G1.1	3	1	1 g	LBK	SS316	1.079	0.361	3
G1.1	3	1	1 g	LBK	SS316	0.736	0.316	4
G1.1	3	1	1 g	LBK	SS316	0.801	0.342	5
G1.1	3	1	1 g	LBK	SS316	1.171	0.283	6
G1.1	3	1	1 g	LBK	SS316	0.802	0.223	7
G1.1	3	1	1 g	LBK	SS316	0.760	0.313	8
G1.1	3	1	1 g	LBK	SS316	1.341	0.292	9
G1.1	3	1	1 g	LBK	SS316	1.154	0.347	10
G1.1	3	1	1 g	LBK	SS316	0.969	0.299	11
G1.1	3	1	1 g	LBK	SS316	0.992	0.350	12
G1.1	3	1	1 g	LBK	SS316	0.678	0.325	13
G1.1	3	1	1 g	LBK	SS316	0.770	0.295	14
G1.1	3	1	1 g	LBK	SS316	1.187	0.325	15
G1.1	3	1	1 g	LBK	SS316	1.020	0.351	16
G1.1	3	1	1 g	LBK	SS316	0.682	0.288	17
G1.1	3	1	1 g	LBK	SS316	1.090	0.321	18
G1.1	3	1	1 g	LBK	SS316	1.022	0.371	19
G1.1	3	1	1 g	LBK	SS316	0.767	0.342	20
G1.1	3	1	1 g	LBK	SS316	0.887	0.360	21
G1.1	3	1	1 g	LBK	SS316	1.262	0.282	22
G1.1	3	1	1 g	LBK	SS316	0.647	0.357	23
G1.1	3	1	1 g	LBK	SS316	0.888	0.471	24
G1.1	3	1	1 g	LBK	SS316	0.681	0.318	25
G1.1	4	1	1 g	LBK	SS316	0.917	0.334	1
G1.1	4	1	1 g	LBK	SS316	1.048	0.383	2
G1.1	4	1	1 g	LBK	SS316	1.205	0.383	3
G1.1	4	1	1 g	LBK	SS316	1.049	0.413	4
G1.1	4	1	1 g	LBK	SS316	1.003	0.383	5
G1.1	4	1	1 g	LBK	SS316	0.897	0.245	6
G1.1	4	1	1 g	LBK	SS316	0.826	0.35	7
G1.1	4	1	1 g	LBK	SS316	0.823	0.336	8
G1.1	4	1	1 g	LBK	SS316	0.916	0.402	9
G1.1	4	1	1 g	LBK	SS316	1.051	0.323	10
G1.1	4	1	1 g	LBK	SS316	0.783	0.288	11
G1.1	4	1	1 g	LBK	SS316	0.983	0.401	12
G1.1	4	1	1 g	LBK	SS316	0.686	0.444	13
G1.1	4	1	1 g	LBK	SS316	0.792	0.24	14
G1.1	4	1	1 g	LBK	SS316	0.838	0.322	15
G1.1	4	1	1 g	LBK	SS316	0.596	0.269	16
G1.1	4	1	1 g	LBK	SS316	0.899	0.392	17
G1.1	4	1	1 g	LBK	SS316	0.891	0.381	18
G1.1	4	1	1 g	LBK	SS316	1.32	0.362	19
G1.1	4	1	1 g	LBK	SS316	0.883	0.431	20
G1.1	4	1	1 g	LBK	SS316	0.851	0.368	21
G1.1	4	1	1 g	LBK	SS316	0.682	0.364	22

G1.1	4	1	1 g	LBK	SS316	0.992	0.389	23
G1.1	4	1	1 g	LBK	SS316	0.62	0.331	24
G1.1	4	1	1 g	LBK	SS316	1.233	0.407	25
G1.5	1	1	1 g	LBK	p-SS316	1.22	0.323	1
G1.5	1	1	1 g	LBK	p-SS316	0.72	0.449	2
G1.5	1	1	1 g	LBK	p-SS316	0.587	0.308	3
G1.5	1	1	1 g	LBK	p-SS316	0.789	0.349	4
G1.5	1	1	1 g	LBK	p-SS316	0.693	0.355	5
G1.5	1	1	1 g	LBK	p-SS316	1.116	0.321	6
G1.5	1	1	1 g	LBK	p-SS316	0.602	0.349	7
G1.5	1	1	1 g	LBK	p-SS316	0.809	0.321	8
G1.5	1	1	1 g	LBK	p-SS316	0.885	0.364	9
G1.5	1	1	1 g	LBK	p-SS316	0.624	0.423	10
G1.5	1	1	1 g	LBK	p-SS316	0.789	0.334	11
G1.5	1	1	1 g	LBK	p-SS316	0.805	0.415	12
G1.5	1	1	1 g	LBK	p-SS316	0.644	0.405	13
G1.5	1	1	1 g	LBK	p-SS316	0.963	0.383	14
G1.5	1	1	1 g	LBK	p-SS316	0.933	0.308	15
G1.5	1	1	1 g	LBK	p-SS316	0.919	0.44	16
G1.5	1	1	1 g	LBK	p-SS316	0.972	0.389	17
G1.5	1	1	1 g	LBK	p-SS316	0.78	0.398	18
G1.5	1	1	1 g	LBK	p-SS316	1.049	0.376	19
G1.5	1	1	1 g	LBK	p-SS316	0.966	0.37	20
G1.5	1	1	1 g	LBK	p-SS316	0.746	0.315	21
G1.5	1	1	1 g	LBK	p-SS316	0.881	0.349	22
G1.5	1	1	1 g	LBK	p-SS316	0.969	0.382	23
G1.5	1	1	1 g	LBK	p-SS316	1.134	0.333	24
G1.5	1	1	1 g	LBK	p-SS316	1.073	0.371	25
G1.5	2	1	1 g	LBK	p-SS316	0.918	0.336	1
G1.5	2	1	1 g	LBK	p-SS316	0.772	0.307	2
G1.5	2	1	1 g	LBK	p-SS316	0.633	0.35	3
G1.5	2	1	1 g	LBK	p-SS316	0.693	0.3	4
G1.5	2	1	1 g	LBK	p-SS316	1.018	0.308	5
G1.5	2	1	1 g	LBK	p-SS316	0.672	0.311	6
G1.5	2	1	1 g	LBK	p-SS316	1.217	0.298	7
G1.5	2	1	1 g	LBK	p-SS316	0.959	0.315	8
G1.5	2	1	1 g	LBK	p-SS316	0.634	0.308	9
G1.5	2	1	1 g	LBK	p-SS316	0.885	0.353	10
G1.5	2	1	1 g	LBK	p-SS316	0.709	0.314	11
G1.5	2	1	1 g	LBK	p-SS316	0.752	0.301	12
G1.5	2	1	1 g	LBK	p-SS316	0.775	0.341	13
G1.5	2	1	1 g	LBK	p-SS316	0.69	0.312	14
G1.5	2	1	1 g	LBK	p-SS316	0.614	0.342	15
G1.5	2	1	1 g	LBK	p-SS316	0.553	0.312	16
G1.5	2	1	1 g	LBK	p-SS316	0.783	0.334	17
G1.5	2	1	1 g	LBK	p-SS316	1.07	0.341	18
G1.5	2	1	1 g	LBK	p-SS316	0.614	0.329	19
G1.5	2	1	1 g	LBK	p-SS316	0.688	0.296	20
G1.5	2	1	1 g	LBK	p-SS316	0.856	0.298	21
G1.5	2	1	1 g	LBK	p-SS316	0.665	0.282	22

G1.5	2	1	1 g	LBK	p-SS316	0.956	0.242	23
G1.5	2	1	1 g	LBK	p-SS316	0.898	0.266	24
G1.5	2	1	1 g	LBK	p-SS316	0.69	0.34	25
G1.5	3	1	1 g	LBK	p-SS316	0.786	0.376	1
G1.5	3	1	1 g	LBK	p-SS316	1.179	0.339	2
G1.5	3	1	1 g	LBK	p-SS316	1.01	0.328	3
G1.5	3	1	1 g	LBK	p-SS316	1.185	0.302	4
G1.5	3	1	1 g	LBK	p-SS316	1.29	0.299	5
G1.5	3	1	1 g	LBK	p-SS316	0.65	0.342	6
G1.5	3	1	1 g	LBK	p-SS316	0.571	0.341	7
G1.5	3	1	1 g	LBK	p-SS316	0.721	0.364	8
G1.5	3	1	1 g	LBK	p-SS316	0.438	0.408	9
G1.5	3	1	1 g	LBK	p-SS316	0.962	0.332	10
G1.5	3	1	1 g	LBK	p-SS316	0.74	0.366	11
G1.5	3	1	1 g	LBK	p-SS316	0.698	0.344	12
G1.5	3	1	1 g	LBK	p-SS316	0.7	0.375	13
G1.5	3	1	1 g	LBK	p-SS316	0.696	0.35	14
G1.5	3	1	1 g	LBK	p-SS316	0.601	0.349	15
G1.5	3	1	1 g	LBK	p-SS316	1.153	0.32	16
G1.5	3	1	1 g	LBK	p-SS316	0.624	0.374	17
G1.5	3	1	1 g	LBK	p-SS316	0.929	0.378	18
G1.5	3	1	1 g	LBK	p-SS316	0.909	0.311	19
G1.5	3	1	1 g	LBK	p-SS316	0.761	0.401	20
G1.5	3	1	1 g	LBK	p-SS316	0.775	0.308	21
G1.5	3	1	1 g	LBK	p-SS316	0.705	0.349	22
G1.5	3	1	1 g	LBK	p-SS316	0.931	0.389	23
G1.5	3	1	1 g	LBK	p-SS316	0.766	0.361	24
G1.5	3	1	1 g	LBK	p-SS316	0.983	0.348	25
G1.5	4	1	1 g	LBK	p-SS316	0.751	0.37	1
G1.5	4	1	1 g	LBK	p-SS316	0.754	0.308	2
G1.5	4	1	1 g	LBK	p-SS316	0.705	0.312	3
G1.5	4	1	1 g	LBK	p-SS316	0.668	0.286	4
G1.5	4	1	1 g	LBK	p-SS316	0.62	0.261	5
G1.5	4	1	1 g	LBK	p-SS316	0.858	0.278	6
G1.5	4	1	1 g	LBK	p-SS316	0.629	0.433	7
G1.5	4	1	1 g	LBK	p-SS316	0.7	0.279	8
G1.5	4	1	1 g	LBK	p-SS316	0.595	0.314	9
G1.5	4	1	1 g	LBK	p-SS316	0.631	0.328	10
G1.5	4	1	1 g	LBK	p-SS316	0.755	0.281	11
G1.5	4	1	1 g	LBK	p-SS316	0.733	0.302	12
G1.5	4	1	1 g	LBK	p-SS316	0.643	0.348	13
G1.5	4	1	1 g	LBK	p-SS316	0.715	0.348	14
G1.5	4	1	1 g	LBK	p-SS316	0.644	0.33	15
G1.5	4	1	1 g	LBK	p-SS316	0.629	0.288	16
G1.5	4	1	1 g	LBK	p-SS316	0.682	0.328	17
G1.5	4	1	1 g	LBK	p-SS316	0.592	0.303	18
G1.5	4	1	1 g	LBK	p-SS316	0.659	0.345	19
G1.5	4	1	1 g	LBK	p-SS316	0.629	0.312	20
G1.5	4	1	1 g	LBK	p-SS316	0.734	0.364	21
G1.5	4	1	1 g	LBK	p-SS316	0.613	0.386	22

G1.5	4	1	1 g	LBK	p-SS316	0.668	0.249	23
G1.5	4	1	1 g	LBK	p-SS316	0.79	0.31	24
G1.5	4	1	1 g	LBK	p-SS316	0.775	0.304	25
G2.1	1	1	1 g	LBK	MIT-LIS	0.925	0.354	1
G2.1	1	1	1 g	LBK	MIT-LIS	1.079	0.373	2
G2.1	1	1	1 g	LBK	MIT-LIS	0.881	0.299	3
G2.1	1	1	1 g	LBK	MIT-LIS	1.104	0.304	4
G2.1	1	1	1 g	LBK	MIT-LIS	0.864	0.262	5
G2.1	1	1	1 g	LBK	MIT-LIS	0.935	0.304	6
G2.1	1	1	1 g	LBK	MIT-LIS	0.691	0.321	7
G2.1	1	1	1 g	LBK	MIT-LIS	0.907	0.361	8
G2.1	1	1	1 g	LBK	MIT-LIS	1.337	0.314	9
G2.1	1	1	1 g	LBK	MIT-LIS	1.063	0.371	10
G2.1	1	1	1 g	LBK	MIT-LIS	1.419	0.39	11
G2.1	1	1	1 g	LBK	MIT-LIS	0.751	0.348	12
G2.1	1	1	1 g	LBK	MIT-LIS	0.806	0.396	13
G2.1	1	1	1 g	LBK	MIT-LIS	1.064	0.352	14
G2.1	1	1	1 g	LBK	MIT-LIS	0.775	0.323	15
G2.1	1	1	1 g	LBK	MIT-LIS	1.122	0.365	16
G2.1	1	1	1 g	LBK	MIT-LIS	1.019	0.34	17
G2.1	1	1	1 g	LBK	MIT-LIS	1.064	0.394	18
G2.1	1	1	1 g	LBK	MIT-LIS	0.829	0.316	19
G2.1	1	1	1 g	LBK	MIT-LIS	1.052	0.362	20
G2.1	1	1	1 g	LBK	MIT-LIS	0.892	0.318	21
G2.1	1	1	1 g	LBK	MIT-LIS	0.652	0.304	22
G2.1	1	1	1 g	LBK	MIT-LIS	0.733	0.329	23
G2.1	1	1	1 g	LBK	MIT-LIS	1.262	0.379	24
G2.1	1	1	1 g	LBK	MIT-LIS	0.703	0.337	25
G2.1	2	1	1 g	LBK	MIT-LIS	1.009	0.384	1
G2.1	2	1	1 g	LBK	MIT-LIS	0.828	0.246	2
G2.1	2	1	1 g	LBK	MIT-LIS	1.019	0.34	3
G2.1	2	1	1 g	LBK	MIT-LIS	1.172	0.392	4
G2.1	2	1	1 g	LBK	MIT-LIS	0.796	0.347	5
G2.1	2	1	1 g	LBK	MIT-LIS	0.89	0.302	6
G2.1	2	1	1 g	LBK	MIT-LIS	0.781	0.342	7
G2.1	2	1	1 g	LBK	MIT-LIS	0.901	0.322	8
G2.1	2	1	1 g	LBK	MIT-LIS	0.788	0.39	9
G2.1	2	1	1 g	LBK	MIT-LIS	0.758	0.358	10
G2.1	2	1	1 g	LBK	MIT-LIS	0.873	0.331	11
G2.1	2	1	1 g	LBK	MIT-LIS	0.985	0.324	12
G2.1	2	1	1 g	LBK	MIT-LIS	0.89	0.336	13
G2.1	2	1	1 g	LBK	MIT-LIS	0.769	0.319	14
G2.1	2	1	1 g	LBK	MIT-LIS	0.759	0.31	15
G2.1	2	1	1 g	LBK	MIT-LIS	0.778	0.318	16
G2.1	2	1	1 g	LBK	MIT-LIS	0.904	0.342	17
G2.1	2	1	1 g	LBK	MIT-LIS	0.894	0.412	18
G2.1	2	1	1 g	LBK	MIT-LIS	1.052	0.415	19
G2.1	2	1	1 g	LBK	MIT-LIS	0.859	0.31	20
G2.1	2	1	1 g	LBK	MIT-LIS	0.936	0.366	21
G2.1	2	1	1 g	LBK	MIT-LIS	0.823	0.337	22

G2.1	2	1	1 g	LBK	MIT-LIS	0.61	0.383	23
G2.1	2	1	1 g	LBK	MIT-LIS	0.67	0.292	24
G2.1	2	1	1 g	LBK	MIT-LIS	1.02	0.324	25
G2.1	3	1	1 g	LBK	MIT-LIS	0.766	0.332	1
G2.1	3	1	1 g	LBK	MIT-LIS	1.253	0.333	2
G2.1	3	1	1 g	LBK	MIT-LIS	0.721	0.337	3
G2.1	3	1	1 g	LBK	MIT-LIS	0.759	0.376	4
G2.1	3	1	1 g	LBK	MIT-LIS	0.889	0.324	5
G2.1	3	1	1 g	LBK	MIT-LIS	0.998	0.308	6
G2.1	3	1	1 g	LBK	MIT-LIS	0.904	0.404	7
G2.1	3	1	1 g	LBK	MIT-LIS	0.727	0.333	8
G2.1	3	1	1 g	LBK	MIT-LIS	0.786	0.363	9
G2.1	3	1	1 g	LBK	MIT-LIS	0.974	0.356	10
G2.1	3	1	1 g	LBK	MIT-LIS	1.176	0.299	11
G2.1	3	1	1 g	LBK	MIT-LIS	0.788	0.333	12
G2.1	3	1	1 g	LBK	MIT-LIS	0.618	0.283	13
G2.1	3	1	1 g	LBK	MIT-LIS	0.888	0.316	14
G2.1	3	1	1 g	LBK	MIT-LIS	0.707	0.321	15
G2.1	3	1	1 g	LBK	MIT-LIS	1.073	0.316	16
G2.1	3	1	1 g	LBK	MIT-LIS	0.584	0.379	17
G2.1	3	1	1 g	LBK	MIT-LIS	1.004	0.271	18
G2.1	3	1	1 g	LBK	MIT-LIS	1.174	0.339	19
G2.1	3	1	1 g	LBK	MIT-LIS	0.914	0.331	20
G2.1	3	1	1 g	LBK	MIT-LIS	0.776	0.38	21
G2.1	3	1	1 g	LBK	MIT-LIS	0.764	0.319	22
G2.1	3	1	1 g	LBK	MIT-LIS	1.089	0.279	23
G2.1	3	1	1 g	LBK	MIT-LIS	0.682	0.373	24
G2.1	3	1	1 g	LBK	MIT-LIS	1.169	0.36	25
G2.1	4	1	1 g	LBK	MIT-LIS	0.88	0.392	1
G2.1	4	1	1 g	LBK	MIT-LIS	0.705	0.463	2
G2.1	4	1	1 g	LBK	MIT-LIS	0.833	0.376	3
G2.1	4	1	1 g	LBK	MIT-LIS	0.692	0.323	4
G2.1	4	1	1 g	LBK	MIT-LIS	0.933	0.41	5
G2.1	4	1	1 g	LBK	MIT-LIS	0.757	0.318	6
G2.1	4	1	1 g	LBK	MIT-LIS	0.693	0.357	7
G2.1	4	1	1 g	LBK	MIT-LIS	0.708	0.31	8
G2.1	4	1	1 g	LBK	MIT-LIS	0.703	0.395	9
G2.1	4	1	1 g	LBK	MIT-LIS	0.613	0.31	10
G2.1	4	1	1 g	LBK	MIT-LIS	0.642	0.336	11
G2.1	4	1	1 g	LBK	MIT-LIS	0.714	0.415	12
G2.1	4	1	1 g	LBK	MIT-LIS	0.833	0.351	13
G2.1	4	1	1 g	LBK	MIT-LIS	0.703	0.324	14
G2.1	4	1	1 g	LBK	MIT-LIS	0.783	0.387	15
G2.1	4	1	1 g	LBK	MIT-LIS	0.827	0.321	16
G2.1	4	1	1 g	LBK	MIT-LIS	0.827	0.438	17
G2.1	4	1	1 g	LBK	MIT-LIS	0.775	0.3	18
G2.1	4	1	1 g	LBK	MIT-LIS	0.871	0.378	19
G2.1	4	1	1 g	LBK	MIT-LIS	0.976	0.438	20
G2.1	4	1	1 g	LBK	MIT-LIS	0.726	0.504	21
G2.1	4	1	1 g	LBK	MIT-LIS	0.722	0.424	22

G2.1	4	1	1 g	LBK	MIT-LIS	0.765	0.298	23
G2.1	4	1	1 g	LBK	MIT-LIS	0.73	0.279	24
G2.1	4	1	1 g	LBK	MIT-LIS	0.665	0.351	25
G7.1	1	2	1 g	LBK	SS316	0.754	0.448	1
G7.1	1	2	1 g	LBK	SS316	0.91	0.33	2
G7.1	1	2	1 g	LBK	SS316	1.03	0.383	3
G7.1	1	2	1 g	LBK	SS316	1.512	0.363	4
G7.1	1	2	1 g	LBK	SS316	1.061	0.344	5
G7.1	1	2	1 g	LBK	SS316	0.883	0.305	6
G7.1	1	2	1 g	LBK	SS316	1.01	0.377	7
G7.1	1	2	1 g	LBK	SS316	1.066	0.441	8
G7.1	1	2	1 g	LBK	SS316	0.771	0.359	9
G7.1	1	2	1 g	LBK	SS316	0.953	0.313	10
G7.1	1	2	1 g	LBK	SS316	0.9	0.369	11
G7.1	1	2	1 g	LBK	SS316	1.231	0.34	12
G7.1	1	2	1 g	LBK	SS316	1.161	0.306	13
G7.1	1	2	1 g	LBK	SS316	1.047	0.327	14
G7.1	1	2	1 g	LBK	SS316	1.061	0.317	15
G7.1	1	2	1 g	LBK	SS316	0.918	0.344	16
G7.1	1	2	1 g	LBK	SS316	1.208	0.321	17
G7.1	1	2	1 g	LBK	SS316	1.026	0.457	18
G7.1	1	2	1 g	LBK	SS316	1.239	0.361	19
G7.1	1	2	1 g	LBK	SS316	1.016	0.38	20
G7.1	1	2	1 g	LBK	SS316	0.93	0.345	21
G7.1	1	2	1 g	LBK	SS316	0.936	0.393	22
G7.1	1	2	1 g	LBK	SS316	0.852	0.383	23
G7.1	1	2	1 g	LBK	SS316	0.766	0.403	24
G7.1	1	2	1 g	LBK	SS316	1.027	0.353	25
G7.1	2	2	1 g	LBK	SS316	0.969	0.344	1
G7.1	2	2	1 g	LBK	SS316	0.94	0.413	2
G7.1	2	2	1 g	LBK	SS316	1.043	0.269	3
G7.1	2	2	1 g	LBK	SS316	1.452	0.298	4
G7.1	2	2	1 g	LBK	SS316	1.138	0.307	5
G7.1	2	2	1 g	LBK	SS316	1.516	0.342	6
G7.1	2	2	1 g	LBK	SS316	1.173	0.393	7
G7.1	2	2	1 g	LBK	SS316	0.978	0.368	8
G7.1	2	2	1 g	LBK	SS316	0.91	0.369	9
G7.1	2	2	1 g	LBK	SS316	1.122	0.404	10
G7.1	2	2	1 g	LBK	SS316	1.322	0.363	11
G7.1	2	2	1 g	LBK	SS316	1.178	0.323	12
G7.1	2	2	1 g	LBK	SS316	1.113	0.292	13
G7.1	2	2	1 g	LBK	SS316	1.146	0.307	14
G7.1	2	2	1 g	LBK	SS316	0.914	0.378	15
G7.1	2	2	1 g	LBK	SS316	0.899	0.323	16
G7.1	2	2	1 g	LBK	SS316	1.129	0.287	17
G7.1	2	2	1 g	LBK	SS316	1.269	0.305	18
G7.1	2	2	1 g	LBK	SS316	1.135	0.38	19
G7.1	2	2	1 g	LBK	SS316	0.711	0.305	20
G7.1	2	2	1 g	LBK	SS316	1.203	0.333	21
G7.1	2	2	1 g	LBK	SS316	1.13	0.28	22

G7.1	2	2	1 g	LBK	SS316	1.26	0.369	23
G7.1	2	2	1 g	LBK	SS316	1.347	0.294	24
G7.1	2	2	1 g	LBK	SS316	1.737	0.498	25
G7.1	3	2	1 g	LBK	SS316	1.311	0.353	1
G7.1	3	2	1 g	LBK	SS316	1.075	0.323	2
G7.1	3	2	1 g	LBK	SS316	1.248	0.34	3
G7.1	3	2	1 g	LBK	SS316	1.182	0.348	4
G7.1	3	2	1 g	LBK	SS316	0.946	0.358	5
G7.1	3	2	1 g	LBK	SS316	1.022	0.313	6
G7.1	3	2	1 g	LBK	SS316	0.682	0.344	7
G7.1	3	2	1 g	LBK	SS316	0.821	0.409	8
G7.1	3	2	1 g	LBK	SS316	1.105	0.306	9
G7.1	3	2	1 g	LBK	SS316	1.164	0.305	10
G7.1	3	2	1 g	LBK	SS316	1.523	0.338	11
G7.1	3	2	1 g	LBK	SS316	1.099	0.335	12
G7.1	3	2	1 g	LBK	SS316	1.141	0.432	13
G7.1	3	2	1 g	LBK	SS316	1.042	0.369	14
G7.1	3	2	1 g	LBK	SS316	1.493	0.466	15
G7.1	3	2	1 g	LBK	SS316	1.081	0.393	16
G7.1	3	2	1 g	LBK	SS316	1.184	0.342	17
G7.1	3	2	1 g	LBK	SS316	1.119	0.369	18
G7.1	3	2	1 g	LBK	SS316	1.218	0.381	19
G7.1	3	2	1 g	LBK	SS316	1.685	0.409	20
G7.1	3	2	1 g	LBK	SS316	1.09	0.445	21
G7.1	3	2	1 g	LBK	SS316	1.113	0.448	22
G7.1	3	2	1 g	LBK	SS316	1.242	0.282	23
G7.1	3	2	1 g	LBK	SS316	1.082	0.282	24
G7.1	3	2	1 g	LBK	SS316	1.437	0.285	25
G7.1	4	2	1 g	LBK	SS316	0.979	0.361	1
G7.1	4	2	1 g	LBK	SS316	1.552	0.386	2
G7.1	4	2	1 g	LBK	SS316	1.203	0.369	3
G7.1	4	2	1 g	LBK	SS316	1.157	0.323	4
G7.1	4	2	1 g	LBK	SS316	0.848	0.366	5
G7.1	4	2	1 g	LBK	SS316	1.241	0.306	6
G7.1	4	2	1 g	LBK	SS316	0.865	0.398	7
G7.1	4	2	1 g	LBK	SS316	0.97	0.384	8
G7.1	4	2	1 g	LBK	SS316	0.869	0.341	9
G7.1	4	2	1 g	LBK	SS316	0.981	0.394	10
G7.1	4	2	1 g	LBK	SS316	0.932	0.38	11
G7.1	4	2	1 g	LBK	SS316	0.716	0.333	12
G7.1	4	2	1 g	LBK	SS316	1.322	0.432	13
G7.1	4	2	1 g	LBK	SS316	1.004	0.323	14
G7.1	4	2	1 g	LBK	SS316	0.953	0.36	15
G7.1	4	2	1 g	LBK	SS316	1.106	0.298	16
G7.1	4	2	1 g	LBK	SS316	0.851	0.489	17
G7.1	4	2	1 g	LBK	SS316	1.276	0.409	18
G7.1	4	2	1 g	LBK	SS316	0.915	0.292	19
G7.1	4	2	1 g	LBK	SS316	1.305	0.381	20
G7.1	4	2	1 g	LBK	SS316	1.12	0.313	21
G7.1	4	2	1 g	LBK	SS316	1.07	0.348	22

G7.1	4	2	1 g	LBK	SS316	0.925	0.348	23
G7.1	4	2	1 g	LBK	SS316	0.815	0.314	24
G7.1	4	2	1 g	LBK	SS316	1.198	0.337	25
G7.5	1	2	1 g	LBK	p-SS316	1.146	0.323	1
G7.5	1	2	1 g	LBK	p-SS316	1.175	0.34	2
G7.5	1	2	1 g	LBK	p-SS316	1.18	0.313	3
G7.5	1	2	1 g	LBK	p-SS316	1.034	0.323	4
G7.5	1	2	1 g	LBK	p-SS316	1.412	0.38	5
G7.5	1	2	1 g	LBK	p-SS316	1.276	0.348	6
G7.5	1	2	1 g	LBK	p-SS316	1.003	0.403	7
G7.5	1	2	1 g	LBK	p-SS316	1.398	0.338	8
G7.5	1	2	1 g	LBK	p-SS316	0.931	0.313	9
G7.5	1	2	1 g	LBK	p-SS316	0.869	0.378	10
G7.5	1	2	1 g	LBK	p-SS316	1.349	0.342	11
G7.5	1	2	1 g	LBK	p-SS316	0.959	0.363	12
G7.5	1	2	1 g	LBK	p-SS316	0.781	0.321	13
G7.5	1	2	1 g	LBK	p-SS316	1.019	0.393	14
G7.5	1	2	1 g	LBK	p-SS316	1.154	0.345	15
G7.5	1	2	1 g	LBK	p-SS316	0.95	0.358	16
G7.5	1	2	1 g	LBK	p-SS316	0.953	0.42	17
G7.5	1	2	1 g	LBK	p-SS316	1.355	0.317	18
G7.5	1	2	1 g	LBK	p-SS316	1.064	0.254	19
G7.5	1	2	1 g	LBK	p-SS316	0.914	0.285	20
G7.5	1	2	1 g	LBK	p-SS316	1.02	0.378	21
G7.5	1	2	1 g	LBK	p-SS316	0.969	0.35	22
G7.5	1	2	1 g	LBK	p-SS316	1.266	0.308	23
G7.5	1	2	1 g	LBK	p-SS316	0.868	0.327	24
G7.5	1	2	1 g	LBK	p-SS316	0.889	0.323	25
G7.5	2	2	1 g	LBK	p-SS316	1.594	0.356	1
G7.5	2	2	1 g	LBK	p-SS316	0.938	0.249	2
G7.5	2	2	1 g	LBK	p-SS316	0.98	0.317	3
G7.5	2	2	1 g	LBK	p-SS316	1.327	0.255	4
G7.5	2	2	1 g	LBK	p-SS316	0.99	0.363	5
G7.5	2	2	1 g	LBK	p-SS316	1.098	0.294	6
G7.5	2	2	1 g	LBK	p-SS316	0.844	0.287	7
G7.5	2	2	1 g	LBK	p-SS316	0.924	0.3	8
G7.5	2	2	1 g	LBK	p-SS316	0.743	0.387	9
G7.5	2	2	1 g	LBK	p-SS316	0.677	0.418	10
G7.5	2	2	1 g	LBK	p-SS316	0.896	0.261	11
G7.5	2	2	1 g	LBK	p-SS316	1.288	0.283	12
G7.5	2	2	1 g	LBK	p-SS316	0.917	0.287	13
G7.5	2	2	1 g	LBK	p-SS316	0.879	0.323	14
G7.5	2	2	1 g	LBK	p-SS316	0.922	0.33	15
G7.5	2	2	1 g	LBK	p-SS316	1.136	0.323	16
G7.5	2	2	1 g	LBK	p-SS316	0.576	0.378	17
G7.5	2	2	1 g	LBK	p-SS316	1.026	0.377	18
G7.5	2	2	1 g	LBK	p-SS316	0.923	0.388	19
G7.5	2	2	1 g	LBK	p-SS316	1	0.42	20
G7.5	2	2	1 g	LBK	p-SS316	0.954	0.273	21
G7.5	2	2	1 g	LBK	p-SS316	0.844	0.396	22

G7.5	2	2	1 g	LBK	p-SS316	0.852	0.266	23
G7.5	2	2	1 g	LBK	p-SS316	1.093	0.233	24
G7.5	2	2	1 g	LBK	p-SS316	1.108	0.357	25
G7.5	3	2	1 g	LBK	p-SS316	0.946	0.393	1
G7.5	3	2	1 g	LBK	p-SS316	1.078	0.393	2
G7.5	3	2	1 g	LBK	p-SS316	1.082	0.439	3
G7.5	3	2	1 g	LBK	p-SS316	0.94	0.313	4
G7.5	3	2	1 g	LBK	p-SS316	0.763	0.345	5
G7.5	3	2	1 g	LBK	p-SS316	0.978	0.344	6
G7.5	3	2	1 g	LBK	p-SS316	1.026	0.386	7
G7.5	3	2	1 g	LBK	p-SS316	1.243	0.329	8
G7.5	3	2	1 g	LBK	p-SS316	0.854	0.33	9
G7.5	3	2	1 g	LBK	p-SS316	1.074	0.369	10
G7.5	3	2	1 g	LBK	p-SS316	1.05	0.333	11
G7.5	3	2	1 g	LBK	p-SS316	1.015	0.373	12
G7.5	3	2	1 g	LBK	p-SS316	0.954	0.409	13
G7.5	3	2	1 g	LBK	p-SS316	0.805	0.274	14
G7.5	3	2	1 g	LBK	p-SS316	1.164	0.342	15
G7.5	3	2	1 g	LBK	p-SS316	0.901	0.451	16
G7.5	3	2	1 g	LBK	p-SS316	1.091	0.409	17
G7.5	3	2	1 g	LBK	p-SS316	1.024	0.406	18
G7.5	3	2	1 g	LBK	p-SS316	1.182	0.377	19
G7.5	3	2	1 g	LBK	p-SS316	1.15	0.391	20
G7.5	3	2	1 g	LBK	p-SS316	0.95	0.377	21
G7.5	3	2	1 g	LBK	p-SS316	1.056	0.363	22
G7.5	3	2	1 g	LBK	p-SS316	0.929	0.373	23
G7.5	3	2	1 g	LBK	p-SS316	1.089	0.313	24
G7.5	3	2	1 g	LBK	p-SS316	0.809	0.302	25
G7.5	4	2	1 g	LBK	p-SS316	1.064	0.338	1
G7.5	4	2	1 g	LBK	p-SS316	1.142	0.38	2
G7.5	4	2	1 g	LBK	p-SS316	1.025	0.409	3
G7.5	4	2	1 g	LBK	p-SS316	1.74	0.342	4
G7.5	4	2	1 g	LBK	p-SS316	0.904	0.47	5
G7.5	4	2	1 g	LBK	p-SS316	0.854	0.359	6
G7.5	4	2	1 g	LBK	p-SS316	1.383	0.352	7
G7.5	4	2	1 g	LBK	p-SS316	1.144	0.358	8
G7.5	4	2	1 g	LBK	p-SS316	1.092	0.373	9
G7.5	4	2	1 g	LBK	p-SS316	1.353	0.373	10
G7.5	4	2	1 g	LBK	p-SS316	0.901	0.366	11
G7.5	4	2	1 g	LBK	p-SS316	0.969	0.459	12
G7.5	4	2	1 g	LBK	p-SS316	1.14	0.38	13
G7.5	4	2	1 g	LBK	p-SS316	1.142	0.393	14
G7.5	4	2	1 g	LBK	p-SS316	1.347	0.418	15
G7.5	4	2	1 g	LBK	p-SS316	1.22	0.42	16
G7.5	4	2	1 g	LBK	p-SS316	0.956	0.325	17
G7.5	4	2	1 g	LBK	p-SS316	1.348	0.401	18
G7.5	4	2	1 g	LBK	p-SS316	1.372	0.477	19
G7.5	4	2	1 g	LBK	p-SS316	1.054	0.398	20
G7.5	4	2	1 g	LBK	p-SS316	1.311	0.348	21
G7.5	4	2	1 g	LBK	p-SS316	1.144	0.338	22

G7.5	4	2	1 g	LBK	p-SS316	1.254	0.47	23
G7.5	4	2	1 g	LBK	p-SS316	0.895	0.344	24
G7.5	4	2	1 g	LBK	p-SS316	1.02	0.3	25
G8.1	1	2	1 g	LBK	MIT-LIS	0.827	0.294	1
G8.1	1	2	1 g	LBK	MIT-LIS	0.756	0.324	2
G8.1	1	2	1 g	LBK	MIT-LIS	0.957	0.331	3
G8.1	1	2	1 g	LBK	MIT-LIS	0.831	0.326	4
G8.1	1	2	1 g	LBK	MIT-LIS	0.931	0.372	5
G8.1	1	2	1 g	LBK	MIT-LIS	0.75	0.39	6
G8.1	1	2	1 g	LBK	MIT-LIS	0.849	0.312	7
G8.1	1	2	1 g	LBK	MIT-LIS	0.7	0.321	8
G8.1	1	2	1 g	LBK	MIT-LIS	0.735	0.311	9
G8.1	1	2	1 g	LBK	MIT-LIS	0.841	0.288	10
G8.1	1	2	1 g	LBK	MIT-LIS	0.7	0.341	11
G8.1	1	2	1 g	LBK	MIT-LIS	0.87	0.341	12
G8.1	1	2	1 g	LBK	MIT-LIS	1.236	0.343	13
G8.1	1	2	1 g	LBK	MIT-LIS	0.716	0.351	14
G8.1	1	2	1 g	LBK	MIT-LIS	0.868	0.379	15
G8.1	1	2	1 g	LBK	MIT-LIS	1.316	0.227	16
G8.1	1	2	1 g	LBK	MIT-LIS	0.577	0.364	17
G8.1	1	2	1 g	LBK	MIT-LIS	0.849	0.278	18
G8.1	1	2	1 g	LBK	MIT-LIS	0.963	0.369	19
G8.1	1	2	1 g	LBK	MIT-LIS	0.846	0.335	20
G8.1	1	2	1 g	LBK	MIT-LIS	1.138	0.33	21
G8.1	1	2	1 g	LBK	MIT-LIS	0.915	0.415	22
G8.1	1	2	1 g	LBK	MIT-LIS	0.904	0.264	23
G8.1	1	2	1 g	LBK	MIT-LIS	0.605	0.378	24
G8.1	1	2	1 g	LBK	MIT-LIS	1.022	0.375	25
G8.1	2	2	1 g	LBK	MIT-LIS	0.835	0.328	1
G8.1	2	2	1 g	LBK	MIT-LIS	0.936	0.36	2
G8.1	2	2	1 g	LBK	MIT-LIS	0.756	0.329	3
G8.1	2	2	1 g	LBK	MIT-LIS	1.031	0.375	4
G8.1	2	2	1 g	LBK	MIT-LIS	0.891	0.324	5
G8.1	2	2	1 g	LBK	MIT-LIS	0.873	0.366	6
G8.1	2	2	1 g	LBK	MIT-LIS	0.9	0.397	7
G8.1	2	2	1 g	LBK	MIT-LIS	1.017	0.363	8
G8.1	2	2	1 g	LBK	MIT-LIS	0.736	0.334	9
G8.1	2	2	1 g	LBK	MIT-LIS	0.848	0.349	10
G8.1	2	2	1 g	LBK	MIT-LIS	0.735	0.336	11
G8.1	2	2	1 g	LBK	MIT-LIS	0.706	0.389	12
G8.1	2	2	1 g	LBK	MIT-LIS	1.131	0.341	13
G8.1	2	2	1 g	LBK	MIT-LIS	1.006	0.427	14
G8.1	2	2	1 g	LBK	MIT-LIS	0.981	0.401	15
G8.1	2	2	1 g	LBK	MIT-LIS	0.845	0.283	16
G8.1	2	2	1 g	LBK	MIT-LIS	0.716	0.3	17
G8.1	2	2	1 g	LBK	MIT-LIS	0.89	0.312	18
G8.1	2	2	1 g	LBK	MIT-LIS	1.005	0.325	19
G8.1	2	2	1 g	LBK	MIT-LIS	0.969	0.308	20
G8.1	2	2	1 g	LBK	MIT-LIS	0.829	0.361	21
G8.1	2	2	1 g	LBK	MIT-LIS	0.786	0.365	22

G8.1	2	2	1 g	LBK	MIT-LIS	0.846	0.36	23
G8.1	2	2	1 g	LBK	MIT-LIS	0.735	0.37	24
G8.1	2	2	1 g	LBK	MIT-LIS	0.789	0.383	25
G8.1	3	2	1 g	LBK	MIT-LIS	0.965	0.297	1
G8.1	3	2	1 g	LBK	MIT-LIS	1.185	0.284	2
G8.1	3	2	1 g	LBK	MIT-LIS	0.844	0.281	3
G8.1	3	2	1 g	LBK	MIT-LIS	1.021	0.39	4
G8.1	3	2	1 g	LBK	MIT-LIS	1.263	0.31	5
G8.1	3	2	1 g	LBK	MIT-LIS	0.786	0.312	6
G8.1	3	2	1 g	LBK	MIT-LIS	0.936	0.267	7
G8.1	3	2	1 g	LBK	MIT-LIS	1.032	0.35	8
G8.1	3	2	1 g	LBK	MIT-LIS	0.871	0.26	9
G8.1	3	2	1 g	LBK	MIT-LIS	1.02	0.319	10
G8.1	3	2	1 g	LBK	MIT-LIS	1.169	0.268	11
G8.1	3	2	1 g	LBK	MIT-LIS	0.56	0.407	12
G8.1	3	2	1 g	LBK	MIT-LIS	0.586	0.271	13
G8.1	3	2	1 g	LBK	MIT-LIS	0.936	0.282	14
G8.1	3	2	1 g	LBK	MIT-LIS	0.77	0.341	15
G8.1	3	2	1 g	LBK	MIT-LIS	0.813	0.331	16
G8.1	3	2	1 g	LBK	MIT-LIS	1.114	0.317	17
G8.1	3	2	1 g	LBK	MIT-LIS	1.046	0.371	18
G8.1	3	2	1 g	LBK	MIT-LIS	0.93	0.365	19
G8.1	3	2	1 g	LBK	MIT-LIS	1.233	0.308	20
G8.1	3	2	1 g	LBK	MIT-LIS	0.847	0.252	21
G8.1	3	2	1 g	LBK	MIT-LIS	0.754	0.323	22
G8.1	3	2	1 g	LBK	MIT-LIS	0.839	0.355	23
G8.1	3	2	1 g	LBK	MIT-LIS	0.779	0.359	24
G8.1	3	2	1 g	LBK	MIT-LIS	0.727	0.329	25
G8.1	4	2	1 g	LBK	MIT-LIS	0.827	0.346	1
G8.1	4	2	1 g	LBK	MIT-LIS	0.795	0.307	2
G8.1	4	2	1 g	LBK	MIT-LIS	0.781	0.328	3
G8.1	4	2	1 g	LBK	MIT-LIS	0.785	0.341	4
G8.1	4	2	1 g	LBK	MIT-LIS	0.771	0.278	5
G8.1	4	2	1 g	LBK	MIT-LIS	0.909	0.355	6
G8.1	4	2	1 g	LBK	MIT-LIS	0.822	0.363	7
G8.1	4	2	1 g	LBK	MIT-LIS	1.019	0.332	8
G8.1	4	2	1 g	LBK	MIT-LIS	0.87	0.379	9
G8.1	4	2	1 g	LBK	MIT-LIS	0.894	0.407	10
G8.1	4	2	1 g	LBK	MIT-LIS	1.106	0.292	11
G8.1	4	2	1 g	LBK	MIT-LIS	0.781	0.369	12
G8.1	4	2	1 g	LBK	MIT-LIS	0.66	0.34	13
G8.1	4	2	1 g	LBK	MIT-LIS	0.852	0.284	14
G8.1	4	2	1 g	LBK	MIT-LIS	0.869	0.375	15
G8.1	4	2	1 g	LBK	MIT-LIS	0.831	0.327	16
G8.1	4	2	1 g	LBK	MIT-LIS	0.93	0.407	17
G8.1	4	2	1 g	LBK	MIT-LIS	0.697	0.321	18
G8.1	4	2	1 g	LBK	MIT-LIS	0.71	0.371	19
G8.1	4	2	1 g	LBK	MIT-LIS	0.702	0.328	20
G8.1	4	2	1 g	LBK	MIT-LIS	0.767	0.411	21
G8.1	4	2	1 g	LBK	MIT-LIS	1.027	0.321	22

G8.1	4	2	1 g	LBK	MIT-LIS	0.724	0.321	23
G8.1	4	2	1 g	LBK	MIT-LIS	0.961	0.324	24
G8.1	4	2	1 g	LBK	MIT-LIS	1.026	0.34	25
G13.1	1	3	1 g	LBK	SS316	1.128	0.323	1
G13.1	1	3	1 g	LBK	SS316	0.977	0.332	2
G13.1	1	3	1 g	LBK	SS316	0.77	0.287	3
G13.1	1	3	1 g	LBK	SS316	0.858	0.269	4
G13.1	1	3	1 g	LBK	SS316	1.025	0.332	5
G13.1	1	3	1 g	LBK	SS316	1.083	0.286	6
G13.1	1	3	1 g	LBK	SS316	0.794	0.295	7
G13.1	1	3	1 g	LBK	SS316	0.751	0.296	8
G13.1	1	3	1 g	LBK	SS316	0.793	0.257	9
G13.1	1	3	1 g	LBK	SS316	0.836	0.296	10
G13.1	1	3	1 g	LBK	SS316	1.052	0.254	11
G13.1	1	3	1 g	LBK	SS316	0.974	0.25	12
G13.1	1	3	1 g	LBK	SS316	1.212	0.284	13
G13.1	1	3	1 g	LBK	SS316	1.023	0.312	14
G13.1	1	3	1 g	LBK	SS316	1.112	0.31	15
G13.1	1	3	1 g	LBK	SS316	1.044	0.351	16
G13.1	1	3	1 g	LBK	SS316	1.004	0.335	17
G13.1	1	3	1 g	LBK	SS316	1.104	0.377	18
G13.1	1	3	1 g	LBK	SS316	1.074	0.298	19
G13.1	1	3	1 g	LBK	SS316	0.872	0.317	20
G13.1	1	3	1 g	LBK	SS316	1.308	0.345	21
G13.1	1	3	1 g	LBK	SS316	0.987	0.306	22
G13.1	1	3	1 g	LBK	SS316	1.047	0.308	23
G13.1	1	3	1 g	LBK	SS316	0.984	0.298	24
G13.1	1	3	1 g	LBK	SS316	1.124	0.305	25
G13.1	2	3	1 g	LBK	SS316	1.077	0.361	1
G13.1	2	3	1 g	LBK	SS316	0.938	0.326	2
G13.1	2	3	1 g	LBK	SS316	1.225	0.425	3
G13.1	2	3	1 g	LBK	SS316	0.971	0.286	4
G13.1	2	3	1 g	LBK	SS316	0.898	0.281	5
G13.1	2	3	1 g	LBK	SS316	0.988	0.318	6
G13.1	2	3	1 g	LBK	SS316	1.022	0.296	7
G13.1	2	3	1 g	LBK	SS316	1.131	0.28	8
G13.1	2	3	1 g	LBK	SS316	1.028	0.342	9
G13.1	2	3	1 g	LBK	SS316	1.094	0.338	10
G13.1	2	3	1 g	LBK	SS316	0.936	0.323	11
G13.1	2	3	1 g	LBK	SS316	0.996	0.358	12
G13.1	2	3	1 g	LBK	SS316	1.172	0.332	13
G13.1	2	3	1 g	LBK	SS316	1.062	0.344	14
G13.1	2	3	1 g	LBK	SS316	1.328	0.35	15
G13.1	2	3	1 g	LBK	SS316	1.032	0.361	16
G13.1	2	3	1 g	LBK	SS316	1.071	0.344	17
G13.1	2	3	1 g	LBK	SS316	1.336	0.289	18
G13.1	2	3	1 g	LBK	SS316	1.079	0.322	19
G13.1	2	3	1 g	LBK	SS316	1.174	0.321	20
G13.1	2	3	1 g	LBK	SS316	1.073	0.29	21
G13.1	2	3	1 g	LBK	SS316	1.082	0.331	22

G13.1	2	3	1 g	LBK	SS316	1.186	0.29	23
G13.1	2	3	1 g	LBK	SS316	1.228	0.373	24
G13.1	2	3	1 g	LBK	SS316	1.098	0.267	25
G13.1	3	3	1 g	LBK	SS316	1.067	0.35	1
G13.1	3	3	1 g	LBK	SS316	1.145	0.328	2
G13.1	3	3	1 g	LBK	SS316	1.498	0.31	3
G13.1	3	3	1 g	LBK	SS316	1.494	0.323	4
G13.1	3	3	1 g	LBK	SS316	1.762	0.219	5
G13.1	3	3	1 g	LBK	SS316	1.066	0.314	6
G13.1	3	3	1 g	LBK	SS316	1.357	0.296	7
G13.1	3	3	1 g	LBK	SS316	0.947	0.32	8
G13.1	3	3	1 g	LBK	SS316	1.053	0.35	9
G13.1	3	3	1 g	LBK	SS316	1.234	0.322	10
G13.1	3	3	1 g	LBK	SS316	1.169	0.379	11
G13.1	3	3	1 g	LBK	SS316	1.662	0.303	12
G13.1	3	3	1 g	LBK	SS316	1.11	0.327	13
G13.1	3	3	1 g	LBK	SS316	0.899	0.248	14
G13.1	3	3	1 g	LBK	SS316	1.216	0.291	15
G13.1	3	3	1 g	LBK	SS316	1.008	0.239	16
G13.1	3	3	1 g	LBK	SS316	0.809	0.284	17
G13.1	3	3	1 g	LBK	SS316	0.842	0.266	18
G13.1	3	3	1 g	LBK	SS316	0.673	0.35	19
G13.1	3	3	1 g	LBK	SS316	1.553	0.283	20
G13.1	3	3	1 g	LBK	SS316	1.081	0.347	21
G13.1	3	3	1 g	LBK	SS316	1.502	0.284	22
G13.1	3	3	1 g	LBK	SS316	1.208	0.399	23
G13.1	3	3	1 g	LBK	SS316	0.949	0.336	24
G13.1	3	3	1 g	LBK	SS316	1.406	0.403	25
G13.1	4	3	1 g	LBK	SS316	1.06	0.355	1
G13.1	4	3	1 g	LBK	SS316	1.08	0.315	2
G13.1	4	3	1 g	LBK	SS316	1.247	0.363	3
G13.1	4	3	1 g	LBK	SS316	1.141	0.261	4
G13.1	4	3	1 g	LBK	SS316	1.152	0.316	5
G13.1	4	3	1 g	LBK	SS316	1.146	0.379	6
G13.1	4	3	1 g	LBK	SS316	1.147	0.248	7
G13.1	4	3	1 g	LBK	SS316	1.142	0.296	8
G13.1	4	3	1 g	LBK	SS316	1.114	0.278	9
G13.1	4	3	1 g	LBK	SS316	1.263	0.317	10
G13.1	4	3	1 g	LBK	SS316	1.25	0.325	11
G13.1	4	3	1 g	LBK	SS316	1.144	0.358	12
G13.1	4	3	1 g	LBK	SS316	1.168	0.301	13
G13.1	4	3	1 g	LBK	SS316	1.032	0.323	14
G13.1	4	3	1 g	LBK	SS316	1.154	0.309	15
G13.1	4	3	1 g	LBK	SS316	1.185	0.363	16
G13.1	4	3	1 g	LBK	SS316	0.956	0.3	17
G13.1	4	3	1 g	LBK	SS316	1.566	0.264	18
G13.1	4	3	1 g	LBK	SS316	0.853	0.323	19
G13.1	4	3	1 g	LBK	SS316	1.451	0.314	20
G13.1	4	3	1 g	LBK	SS316	0.935	0.352	21
G13.1	4	3	1 g	LBK	SS316	0.905	0.308	22

G13.1	4	3	1 g	LBK	SS316	1.261	0.312	23
G13.1	4	3	1 g	LBK	SS316	1.025	0.323	24
G13.1	4	3	1 g	LBK	SS316	1.268	0.328	25
G13.5	1	3	1 g	LBK	p-SS316	1.317	0.325	1
G13.5	1	3	1 g	LBK	p-SS316	1.562	0.277	2
G13.5	1	3	1 g	LBK	p-SS316	1.533	0.31	3
G13.5	1	3	1 g	LBK	p-SS316	0.816	0.274	4
G13.5	1	3	1 g	LBK	p-SS316	0.854	0.27	5
G13.5	1	3	1 g	LBK	p-SS316	0.895	0.284	6
G13.5	1	3	1 g	LBK	p-SS316	1.118	0.285	7
G13.5	1	3	1 g	LBK	p-SS316	1.066	0.362	8
G13.5	1	3	1 g	LBK	p-SS316	1.584	0.254	9
G13.5	1	3	1 g	LBK	p-SS316	1.16	0.336	10
G13.5	1	3	1 g	LBK	p-SS316	0.987	0.269	11
G13.5	1	3	1 g	LBK	p-SS316	1.274	0.269	12
G13.5	1	3	1 g	LBK	p-SS316	1.326	0.349	13
G13.5	1	3	1 g	LBK	p-SS316	1.346	0.235	14
G13.5	1	3	1 g	LBK	p-SS316	0.799	0.287	15
G13.5	1	3	1 g	LBK	p-SS316	0.921	0.288	16
G13.5	1	3	1 g	LBK	p-SS316	1.319	0.27	17
G13.5	1	3	1 g	LBK	p-SS316	0.947	0.296	18
G13.5	1	3	1 g	LBK	p-SS316	1.066	0.349	19
G13.5	1	3	1 g	LBK	p-SS316	1.381	0.346	20
G13.5	1	3	1 g	LBK	p-SS316	0.98	0.314	21
G13.5	1	3	1 g	LBK	p-SS316	0.902	0.269	22
G13.5	1	3	1 g	LBK	p-SS316	1.492	0.277	23
G13.5	1	3	1 g	LBK	p-SS316	1.381	0.329	24
G13.5	1	3	1 g	LBK	p-SS316	0.97	0.304	25
G13.5	2	3	1 g	LBK	p-SS316	1.522	0.371	1
G13.5	2	3	1 g	LBK	p-SS316	1.458	0.269	2
G13.5	2	3	1 g	LBK	p-SS316	1.257	0.295	3
G13.5	2	3	1 g	LBK	p-SS316	1.082	0.307	4
G13.5	2	3	1 g	LBK	p-SS316	0.933	0.295	5
G13.5	2	3	1 g	LBK	p-SS316	1.701	0.269	6
G13.5	2	3	1 g	LBK	p-SS316	1.353	0.325	7
G13.5	2	3	1 g	LBK	p-SS316	1.503	0.267	8
G13.5	2	3	1 g	LBK	p-SS316	1.376	0.247	9
G13.5	2	3	1 g	LBK	p-SS316	1.26	0.361	10
G13.5	2	3	1 g	LBK	p-SS316	1.099	0.349	11
G13.5	2	3	1 g	LBK	p-SS316	1.697	0.292	12
G13.5	2	3	1 g	LBK	p-SS316	1.351	0.318	13
G13.5	2	3	1 g	LBK	p-SS316	1.138	0.258	14
G13.5	2	3	1 g	LBK	p-SS316	1.457	0.298	15
G13.5	2	3	1 g	LBK	p-SS316	0.643	0.289	16
G13.5	2	3	1 g	LBK	p-SS316	1.517	0.258	17
G13.5	2	3	1 g	LBK	p-SS316	1.282	0.28	18
G13.5	2	3	1 g	LBK	p-SS316	1.147	0.244	19
G13.5	2	3	1 g	LBK	p-SS316	1.101	0.321	20
G13.5	2	3	1 g	LBK	p-SS316	1.632	0.257	21
G13.5	2	3	1 g	LBK	p-SS316	1.405	0.258	22

G13.5	2	3	1 g	LBK	p-SS316	1.182	0.323	23
G13.5	2	3	1 g	LBK	p-SS316	0.9	0.28	24
G13.5	2	3	1 g	LBK	p-SS316	1.703	0.252	25
G13.5	3	3	1 g	LBK	p-SS316	0.913	0.309	1
G13.5	3	3	1 g	LBK	p-SS316	1.388	0.341	2
G13.5	3	3	1 g	LBK	p-SS316	1.166	0.323	3
G13.5	3	3	1 g	LBK	p-SS316	1.209	0.251	4
G13.5	3	3	1 g	LBK	p-SS316	0.928	0.38	5
G13.5	3	3	1 g	LBK	p-SS316	1.242	0.345	6
G13.5	3	3	1 g	LBK	p-SS316	0.968	0.344	7
G13.5	3	3	1 g	LBK	p-SS316	1.22	0.366	8
G13.5	3	3	1 g	LBK	p-SS316	1.19	0.325	9
G13.5	3	3	1 g	LBK	p-SS316	1.419	0.337	10
G13.5	3	3	1 g	LBK	p-SS316	0.986	0.337	11
G13.5	3	3	1 g	LBK	p-SS316	1.175	0.346	12
G13.5	3	3	1 g	LBK	p-SS316	1.483	0.321	13
G13.5	3	3	1 g	LBK	p-SS316	1.028	0.362	14
G13.5	3	3	1 g	LBK	p-SS316	1.202	0.361	15
G13.5	3	3	1 g	LBK	p-SS316	1.267	0.397	16
G13.5	3	3	1 g	LBK	p-SS316	1.022	0.39	17
G13.5	3	3	1 g	LBK	p-SS316	0.932	0.321	18
G13.5	3	3	1 g	LBK	p-SS316	0.982	0.383	19
G13.5	3	3	1 g	LBK	p-SS316	1.022	0.306	20
G13.5	3	3	1 g	LBK	p-SS316	0.917	0.325	21
G13.5	3	3	1 g	LBK	p-SS316	0.894	0.325	22
G13.5	3	3	1 g	LBK	p-SS316	1.092	0.353	23
G13.5	3	3	1 g	LBK	p-SS316	1.233	0.323	24
G13.5	3	3	1 g	LBK	p-SS316	1.024	0.334	25
G13.5	4	3	1 g	LBK	p-SS316	0.975	0.296	1
G13.5	4	3	1 g	LBK	p-SS316	1.113	0.277	2
G13.5	4	3	1 g	LBK	p-SS316	1.067	0.258	3
G13.5	4	3	1 g	LBK	p-SS316	1.152	0.344	4
G13.5	4	3	1 g	LBK	p-SS316	1.416	0.366	5
G13.5	4	3	1 g	LBK	p-SS316	1.467	0.325	6
G13.5	4	3	1 g	LBK	p-SS316	1.29	0.313	7
G13.5	4	3	1 g	LBK	p-SS316	1.015	0.258	8
G13.5	4	3	1 g	LBK	p-SS316	0.928	0.3	9
G13.5	4	3	1 g	LBK	p-SS316	1.382	0.274	10
G13.5	4	3	1 g	LBK	p-SS316	1.652	0.353	11
G13.5	4	3	1 g	LBK	p-SS316	0.699	0.233	12
G13.5	4	3	1 g	LBK	p-SS316	1.451	0.274	13
G13.5	4	3	1 g	LBK	p-SS316	0.99	0.289	14
G13.5	4	3	1 g	LBK	p-SS316	1.066	0.317	15
G13.5	4	3	1 g	LBK	p-SS316	0.681	0.329	16
G13.5	4	3	1 g	LBK	p-SS316	1.089	0.258	17
G13.5	4	3	1 g	LBK	p-SS316	1.288	0.274	18
G13.5	4	3	1 g	LBK	p-SS316	1.579	0.321	19
G13.5	4	3	1 g	LBK	p-SS316	1.153	0.304	20
G13.5	4	3	1 g	LBK	p-SS316	1.165	0.296	21
G13.5	4	3	1 g	LBK	p-SS316	1.058	0.307	22

G13.5	4	3	1 g	LBK	p-SS316	1.278	0.312	23
G13.5	4	3	1 g	LBK	p-SS316	1.235	0.352	24
G13.5	4	3	1 g	LBK	p-SS316	1.216	0.319	25
G14.1	1	3	1 g	LBK	MIT-LIS	1.49	0.303	1
G14.1	1	3	1 g	LBK	MIT-LIS	1.318	0.284	2
G14.1	1	3	1 g	LBK	MIT-LIS	1.157	0.352	3
G14.1	1	3	1 g	LBK	MIT-LIS	1.017	0.224	4
G14.1	1	3	1 g	LBK	MIT-LIS	0.895	0.285	5
G14.1	1	3	1 g	LBK	MIT-LIS	1.358	0.289	6
G14.1	1	3	1 g	LBK	MIT-LIS	0.831	0.302	7
G14.1	1	3	1 g	LBK	MIT-LIS	0.727	0.284	8
G14.1	1	3	1 g	LBK	MIT-LIS	0.685	0.319	9
G14.1	1	3	1 g	LBK	MIT-LIS	1.177	0.283	10
G14.1	1	3	1 g	LBK	MIT-LIS	1.46	0.284	11
G14.1	1	3	1 g	LBK	MIT-LIS	1.102	0.309	12
G14.1	1	3	1 g	LBK	MIT-LIS	0.971	0.276	13
G14.1	1	3	1 g	LBK	MIT-LIS	1.229	0.239	14
G14.1	1	3	1 g	LBK	MIT-LIS	0.903	0.284	15
G14.1	1	3	1 g	LBK	MIT-LIS	0.857	0.288	16
G14.1	1	3	1 g	LBK	MIT-LIS	1.067	0.317	17
G14.1	1	3	1 g	LBK	MIT-LIS	0.964	0.331	18
G14.1	1	3	1 g	LBK	MIT-LIS	0.941	0.257	19
G14.1	1	3	1 g	LBK	MIT-LIS	0.673	0.287	20
G14.1	1	3	1 g	LBK	MIT-LIS	1.326	0.302	21
G14.1	1	3	1 g	LBK	MIT-LIS	0.92	0.327	22
G14.1	1	3	1 g	LBK	MIT-LIS	0.887	0.336	23
G14.1	1	3	1 g	LBK	MIT-LIS	0.704	0.288	24
G14.1	1	3	1 g	LBK	MIT-LIS	0.711	0.306	25
G14.1	2	3	1 g	LBK	MIT-LIS	0.795	0.323	1
G14.1	2	3	1 g	LBK	MIT-LIS	0.98	0.306	2
G14.1	2	3	1 g	LBK	MIT-LIS	1.145	0.313	3
G14.1	2	3	1 g	LBK	MIT-LIS	0.662	0.312	4
G14.1	2	3	1 g	LBK	MIT-LIS	0.789	0.314	5
G14.1	2	3	1 g	LBK	MIT-LIS	0.856	0.313	6
G14.1	2	3	1 g	LBK	MIT-LIS	0.914	0.295	7
G14.1	2	3	1 g	LBK	MIT-LIS	0.805	0.301	8
G14.1	2	3	1 g	LBK	MIT-LIS	0.688	0.309	9
G14.1	2	3	1 g	LBK	MIT-LIS	0.83	0.25	10
G14.1	2	3	1 g	LBK	MIT-LIS	0.793	0.323	11
G14.1	2	3	1 g	LBK	MIT-LIS	1.432	0.333	12
G14.1	2	3	1 g	LBK	MIT-LIS	1.016	0.376	13
G14.1	2	3	1 g	LBK	MIT-LIS	1.095	0.267	14
G14.1	2	3	1 g	LBK	MIT-LIS	1.079	0.333	15
G14.1	2	3	1 g	LBK	MIT-LIS	1.067	0.258	16
G14.1	2	3	1 g	LBK	MIT-LIS	1.125	0.313	17
G14.1	2	3	1 g	LBK	MIT-LIS	0.759	0.283	18
G14.1	2	3	1 g	LBK	MIT-LIS	1.411	0.313	19
G14.1	2	3	1 g	LBK	MIT-LIS	0.997	0.353	20
G14.1	2	3	1 g	LBK	MIT-LIS	0.894	0.34	21
G14.1	2	3	1 g	LBK	MIT-LIS	1.004	0.242	22

G14.1	2	3	1 g	LBK	MIT-LIS	1.076	0.298	23
G14.1	2	3	1 g	LBK	MIT-LIS	0.956	0.309	24
G14.1	2	3	1 g	LBK	MIT-LIS	0.97	0.296	25
G14.1	3	3	1 g	LBK	MIT-LIS	0.887	0.366	1
G14.1	3	3	1 g	LBK	MIT-LIS	1.611	0.289	2
G14.1	3	3	1 g	LBK	MIT-LIS	0.826	0.396	3
G14.1	3	3	1 g	LBK	MIT-LIS	0.93	0.326	4
G14.1	3	3	1 g	LBK	MIT-LIS	1.166	0.341	5
G14.1	3	3	1 g	LBK	MIT-LIS	0.713	0.305	6
G14.1	3	3	1 g	LBK	MIT-LIS	1.31	0.336	7
G14.1	3	3	1 g	LBK	MIT-LIS	0.798	0.281	8
G14.1	3	3	1 g	LBK	MIT-LIS	1.306	0.321	9
G14.1	3	3	1 g	LBK	MIT-LIS	0.908	0.328	10
G14.1	3	3	1 g	LBK	MIT-LIS	1.146	0.352	11
G14.1	3	3	1 g	LBK	MIT-LIS	0.772	0.305	12
G14.1	3	3	1 g	LBK	MIT-LIS	1.308	0.278	13
G14.1	3	3	1 g	LBK	MIT-LIS	1.67	0.274	14
G14.1	3	3	1 g	LBK	MIT-LIS	1.036	0.296	15
G14.1	3	3	1 g	LBK	MIT-LIS	1.51	0.342	16
G14.1	3	3	1 g	LBK	MIT-LIS	1.16	0.33	17
G14.1	3	3	1 g	LBK	MIT-LIS	1.257	0.369	18
G14.1	3	3	1 g	LBK	MIT-LIS	1.026	0.365	19
G14.1	3	3	1 g	LBK	MIT-LIS	1.405	0.306	20
G14.1	3	3	1 g	LBK	MIT-LIS	1.119	0.291	21
G14.1	3	3	1 g	LBK	MIT-LIS	0.887	0.374	22
G14.1	3	3	1 g	LBK	MIT-LIS	1.385	0.363	23
G14.1	3	3	1 g	LBK	MIT-LIS	0.882	0.344	24
G14.1	3	3	1 g	LBK	MIT-LIS	0.866	0.377	25
G14.1	4	3	1 g	LBK	MIT-LIS	0.787	0.265	1
G14.1	4	3	1 g	LBK	MIT-LIS	0.792	0.332	2
G14.1	4	3	1 g	LBK	MIT-LIS	0.986	0.29	3
G14.1	4	3	1 g	LBK	MIT-LIS	1.377	0.28	4
G14.1	4	3	1 g	LBK	MIT-LIS	0.579	0.315	5
G14.1	4	3	1 g	LBK	MIT-LIS	1.398	0.319	6
G14.1	4	3	1 g	LBK	MIT-LIS	1.278	0.327	7
G14.1	4	3	1 g	LBK	MIT-LIS	1.079	0.276	8
G14.1	4	3	1 g	LBK	MIT-LIS	0.809	0.324	9
G14.1	4	3	1 g	LBK	MIT-LIS	0.854	0.265	10
G14.1	4	3	1 g	LBK	MIT-LIS	0.738	0.385	11
G14.1	4	3	1 g	LBK	MIT-LIS	1.086	0.339	12
G14.1	4	3	1 g	LBK	MIT-LIS	0.789	0.277	13
G14.1	4	3	1 g	LBK	MIT-LIS	1.877	0.235	14
G14.1	4	3	1 g	LBK	MIT-LIS	1.122	0.239	15
G14.1	4	3	1 g	LBK	MIT-LIS	0.802	0.302	16
G14.1	4	3	1 g	LBK	MIT-LIS	0.854	0.272	17
G14.1	4	3	1 g	LBK	MIT-LIS	1.464	0.328	18
G14.1	4	3	1 g	LBK	MIT-LIS	0.992	0.328	19
G14.1	4	3	1 g	LBK	MIT-LIS	1.252	0.347	20
G14.1	4	3	1 g	LBK	MIT-LIS	1.514	0.315	21
G14.1	4	3	1 g	LBK	MIT-LIS	1.353	0.325	22

G14.1	4	3	1 g	LBK	MIT-LIS	1.027	0.234	23
G14.1	4	3	1 g	LBK	MIT-LIS	1.133	0.319	24
G14.1	4	3	1 g	LBK	MIT-LIS	1.031	0.249	25
1.1	1	1	µg	LBK	SS316	0.6	0.342	1
1.1	1	1	µg	LBK	SS316	0.663	0.331	2
1.1	1	1	µg	LBK	SS316	0.795	0.279	3
1.1	1	1	µg	LBK	SS316	0.938	0.251	4
1.1	1	1	µg	LBK	SS316	0.848	0.343	5
1.1	1	1	µg	LBK	SS316	0.724	0.389	6
1.1	1	1	µg	LBK	SS316	0.721	0.375	7
1.1	1	1	µg	LBK	SS316	0.83	0.322	8
1.1	1	1	µg	LBK	SS316	1.191	0.341	9
1.1	1	1	µg	LBK	SS316	0.808	0.365	10
1.1	1	1	µg	LBK	SS316	0.687	0.325	11
1.1	1	1	µg	LBK	SS316	0.557	0.392	12
1.1	1	1	µg	LBK	SS316	0.896	0.354	13
1.1	1	1	µg	LBK	SS316	0.812	0.396	14
1.1	1	1	µg	LBK	SS316	0.845	0.375	15
1.1	1	1	µg	LBK	SS316	1.075	0.526	16
1.1	1	1	µg	LBK	SS316	0.661	0.383	17
1.1	1	1	µg	LBK	SS316	0.581	0.354	18
1.1	1	1	µg	LBK	SS316	0.741	0.367	19
1.1	1	1	µg	LBK	SS316	0.968	0.474	20
1.1	1	1	µg	LBK	SS316	0.813	0.322	21
1.1	1	1	µg	LBK	SS316	0.711	0.459	22
1.1	1	1	µg	LBK	SS316	1.005	0.265	23
1.1	1	1	µg	LBK	SS316	1.2	0.409	24
1.1	1	1	µg	LBK	SS316	0.778	0.35	25
1.1	2	1	µg	LBK	SS316	1.138	0.337	1
1.1	2	1	µg	LBK	SS316	0.956	0.379	2
1.1	2	1	µg	LBK	SS316	1.062	0.336	3
1.1	2	1	µg	LBK	SS316	0.812	0.258	4
1.1	2	1	µg	LBK	SS316	1.074	0.3	5
1.1	2	1	µg	LBK	SS316	0.972	0.342	6
1.1	2	1	µg	LBK	SS316	0.759	0.384	7
1.1	2	1	µg	LBK	SS316	0.825	0.301	8
1.1	2	1	µg	LBK	SS316	0.96	0.523	9
1.1	2	1	µg	LBK	SS316	0.999	0.313	10
1.1	2	1	µg	LBK	SS316	0.885	0.346	11
1.1	2	1	µg	LBK	SS316	0.83	0.293	12
1.1	2	1	µg	LBK	SS316	0.631	0.29	13
1.1	2	1	µg	LBK	SS316	1.005	0.354	14
1.1	2	1	µg	LBK	SS316	0.842	0.346	15
1.1	2	1	µg	LBK	SS316	0.792	0.307	16
1.1	2	1	µg	LBK	SS316	0.808	0.333	17
1.1	2	1	µg	LBK	SS316	0.794	0.35	18
1.1	2	1	µg	LBK	SS316	1.036	0.425	19
1.1	2	1	µg	LBK	SS316	0.988	0.419	20
1.1	2	1	µg	LBK	SS316	0.941	0.333	21
1.1	2	1	µg	LBK	SS316	1.086	0.375	22

1.1	2	1	µg	LBK	SS316	0.855	0.409	23
1.1	2	1	µg	LBK	SS316	0.744	0.273	24
1.1	2	1	µg	LBK	SS316	1.181	0.373	25
1.1	3	1	µg	LBK	SS316	0.696	0.324	1
1.1	3	1	µg	LBK	SS316	0.753	0.422	2
1.1	3	1	µg	LBK	SS316	0.82	0.456	3
1.1	3	1	µg	LBK	SS316	0.586	0.353	4
1.1	3	1	µg	LBK	SS316	0.695	0.377	5
1.1	3	1	µg	LBK	SS316	0.632	0.381	6
1.1	3	1	µg	LBK	SS316	0.735	0.333	7
1.1	3	1	µg	LBK	SS316	0.795	0.326	8
1.1	3	1	µg	LBK	SS316	0.922	0.353	9
1.1	3	1	µg	LBK	SS316	0.598	0.389	10
1.1	3	1	µg	LBK	SS316	0.674	0.375	11
1.1	3	1	µg	LBK	SS316	0.754	0.292	12
1.1	3	1	µg	LBK	SS316	0.825	0.39	13
1.1	3	1	µg	LBK	SS316	0.633	0.323	14
1.1	3	1	µg	LBK	SS316	0.608	0.382	15
1.1	3	1	µg	LBK	SS316	0.755	0.344	16
1.1	3	1	µg	LBK	SS316	0.597	0.455	17
1.1	3	1	µg	LBK	SS316	1.018	0.293	18
1.1	3	1	µg	LBK	SS316	0.711	0.413	19
1.1	3	1	µg	LBK	SS316	0.732	0.29	20
1.1	3	1	µg	LBK	SS316	0.717	0.296	21
1.1	3	1	µg	LBK	SS316	0.852	0.329	22
1.1	3	1	µg	LBK	SS316	0.962	0.324	23
1.1	3	1	µg	LBK	SS316	0.809	0.304	24
1.1	3	1	µg	LBK	SS316	0.904	0.394	25
1.1	4	1	µg	LBK	SS316	0.717	0.334	1
1.1	4	1	µg	LBK	SS316	1.054	0.288	2
1.1	4	1	µg	LBK	SS316	0.911	0.304	3
1.1	4	1	µg	LBK	SS316	0.828	0.357	4
1.1	4	1	µg	LBK	SS316	0.988	0.221	5
1.1	4	1	µg	LBK	SS316	0.679	0.354	6
1.1	4	1	µg	LBK	SS316	0.654	0.367	7
1.1	4	1	µg	LBK	SS316	0.957	0.317	8
1.1	4	1	µg	LBK	SS316	0.637	0.367	9
1.1	4	1	µg	LBK	SS316	0.999	0.333	10
1.1	4	1	µg	LBK	SS316	0.998	0.389	11
1.1	4	1	µg	LBK	SS316	0.84	0.299	12
1.1	4	1	µg	LBK	SS316	1.052	0.333	13
1.1	4	1	µg	LBK	SS316	1.322	0.334	14
1.1	4	1	µg	LBK	SS316	0.919	0.379	15
1.1	4	1	µg	LBK	SS316	0.909	0.285	16
1.1	4	1	µg	LBK	SS316	0.813	0.342	17
1.1	4	1	µg	LBK	SS316	0.691	0.346	18
1.1	4	1	µg	LBK	SS316	1.007	0.41	19
1.1	4	1	µg	LBK	SS316	0.932	0.337	20
1.1	4	1	µg	LBK	SS316	1.058	0.243	21
1.1	4	1	µg	LBK	SS316	0.764	0.379	22

1.1	4	1	µg	LBK	SS316	0.642	0.372	23
1.1	4	1	µg	LBK	SS316	0.563	0.349	24
1.1	4	1	µg	LBK	SS316	1.047	0.396	25
1.5	1	1	µg	LBK	p-SS316	0.874	0.38	1
1.5	1	1	µg	LBK	p-SS316	0.789	0.303	2
1.5	1	1	µg	LBK	p-SS316	0.702	0.361	3
1.5	1	1	µg	LBK	p-SS316	0.91	0.292	4
1.5	1	1	µg	LBK	p-SS316	0.912	0.279	5
1.5	1	1	µg	LBK	p-SS316	0.729	0.31	6
1.5	1	1	µg	LBK	p-SS316	0.902	0.327	7
1.5	1	1	µg	LBK	p-SS316	0.801	0.317	8
1.5	1	1	µg	LBK	p-SS316	0.859	0.418	9
1.5	1	1	µg	LBK	p-SS316	0.851	0.337	10
1.5	1	1	µg	LBK	p-SS316	0.78	0.336	11
1.5	1	1	µg	LBK	p-SS316	0.903	0.327	12
1.5	1	1	µg	LBK	p-SS316	0.846	0.347	13
1.5	1	1	µg	LBK	p-SS316	0.859	0.44	14
1.5	1	1	µg	LBK	p-SS316	0.632	0.377	15
1.5	1	1	µg	LBK	p-SS316	0.983	0.274	16
1.5	1	1	µg	LBK	p-SS316	0.774	0.269	17
1.5	1	1	µg	LBK	p-SS316	0.807	0.265	18
1.5	1	1	µg	LBK	p-SS316	0.792	0.295	19
1.5	1	1	µg	LBK	p-SS316	0.626	0.346	20
1.5	1	1	µg	LBK	p-SS316	1.052	0.369	21
1.5	1	1	µg	LBK	p-SS316	0.896	0.398	22
1.5	1	1	µg	LBK	p-SS316	0.875	0.341	23
1.5	1	1	µg	LBK	p-SS316	0.937	0.319	24
1.5	1	1	µg	LBK	p-SS316	0.972	0.442	25
1.5	2	1	µg	LBK	p-SS316	0.76	0.346	1
1.5	2	1	µg	LBK	p-SS316	0.727	0.307	2
1.5	2	1	µg	LBK	p-SS316	0.713	0.319	3
1.5	2	1	µg	LBK	p-SS316	0.807	0.25	4
1.5	2	1	µg	LBK	p-SS316	0.706	0.326	5
1.5	2	1	µg	LBK	p-SS316	0.783	0.247	6
1.5	2	1	µg	LBK	p-SS316	0.674	0.316	7
1.5	2	1	µg	LBK	p-SS316	0.74	0.337	8
1.5	2	1	µg	LBK	p-SS316	1.014	0.38	9
1.5	2	1	µg	LBK	p-SS316	0.843	0.31	10
1.5	2	1	µg	LBK	p-SS316	0.679	0.354	11
1.5	2	1	µg	LBK	p-SS316	0.88	0.268	12
1.5	2	1	µg	LBK	p-SS316	0.871	0.341	13
1.5	2	1	µg	LBK	p-SS316	0.836	0.243	14
1.5	2	1	µg	LBK	p-SS316	0.668	0.279	15
1.5	2	1	µg	LBK	p-SS316	0.863	0.323	16
1.5	2	1	µg	LBK	p-SS316	0.733	0.373	17
1.5	2	1	µg	LBK	p-SS316	0.746	0.347	18
1.5	2	1	µg	LBK	p-SS316	0.794	0.356	19
1.5	2	1	µg	LBK	p-SS316	0.893	0.294	20
1.5	2	1	µg	LBK	p-SS316	0.961	0.289	21
1.5	2	1	µg	LBK	p-SS316	1.026	0.327	22

1.5	2	1	µg	LBK	p-SS316	0.714	0.327	23
1.5	2	1	µg	LBK	p-SS316	0.884	0.274	24
1.5	2	1	µg	LBK	p-SS316	0.782	0.336	25
1.5	3	1	µg	LBK	p-SS316	0.955	0.313	1
1.5	3	1	µg	LBK	p-SS316	0.57	0.346	2
1.5	3	1	µg	LBK	p-SS316	0.711	0.387	3
1.5	3	1	µg	LBK	p-SS316	0.775	0.322	4
1.5	3	1	µg	LBK	p-SS316	0.596	0.365	5
1.5	3	1	µg	LBK	p-SS316	0.543	0.367	6
1.5	3	1	µg	LBK	p-SS316	0.731	0.341	7
1.5	3	1	µg	LBK	p-SS316	0.811	0.319	8
1.5	3	1	µg	LBK	p-SS316	0.823	0.317	9
1.5	3	1	µg	LBK	p-SS316	0.565	0.305	10
1.5	3	1	µg	LBK	p-SS316	0.729	0.299	11
1.5	3	1	µg	LBK	p-SS316	0.835	0.328	12
1.5	3	1	µg	LBK	p-SS316	0.686	0.333	13
1.5	3	1	µg	LBK	p-SS316	0.844	0.347	14
1.5	3	1	µg	LBK	p-SS316	0.665	0.333	15
1.5	3	1	µg	LBK	p-SS316	0.919	0.375	16
1.5	3	1	µg	LBK	p-SS316	0.67	0.277	17
1.5	3	1	µg	LBK	p-SS316	1.02	0.32	18
1.5	3	1	µg	LBK	p-SS316	0.733	0.31	19
1.5	3	1	µg	LBK	p-SS316	0.847	0.319	20
1.5	3	1	µg	LBK	p-SS316	1.039	0.317	21
1.5	3	1	µg	LBK	p-SS316	0.713	0.35	22
1.5	3	1	µg	LBK	p-SS316	0.692	0.31	23
1.5	3	1	µg	LBK	p-SS316	0.721	0.333	24
1.5	3	1	µg	LBK	p-SS316	0.765	0.31	25
1.5	4	1	µg	LBK	p-SS316	0.842	0.301	1
1.5	4	1	µg	LBK	p-SS316	0.837	0.307	2
1.5	4	1	µg	LBK	p-SS316	0.741	0.303	3
1.5	4	1	µg	LBK	p-SS316	0.828	0.346	4
1.5	4	1	µg	LBK	p-SS316	0.733	0.361	5
1.5	4	1	µg	LBK	p-SS316	0.965	0.317	6
1.5	4	1	µg	LBK	p-SS316	0.848	0.286	7
1.5	4	1	µg	LBK	p-SS316	0.795	0.387	8
1.5	4	1	µg	LBK	p-SS316	0.793	0.305	9
1.5	4	1	µg	LBK	p-SS316	0.902	0.307	10
1.5	4	1	µg	LBK	p-SS316	0.922	0.301	11
1.5	4	1	µg	LBK	p-SS316	0.927	0.334	12
1.5	4	1	µg	LBK	p-SS316	0.739	0.342	13
1.5	4	1	µg	LBK	p-SS316	0.797	0.283	14
1.5	4	1	µg	LBK	p-SS316	0.711	0.317	15
1.5	4	1	µg	LBK	p-SS316	0.764	0.341	16
1.5	4	1	µg	LBK	p-SS316	0.707	0.288	17
1.5	4	1	µg	LBK	p-SS316	1.003	0.334	18
1.5	4	1	µg	LBK	p-SS316	0.852	0.318	19
1.5	4	1	µg	LBK	p-SS316	0.831	0.317	20
1.5	4	1	µg	LBK	p-SS316	1.009	0.299	21
1.5	4	1	µg	LBK	p-SS316	0.888	0.33	22

1.5	4	1	µg	LBK	p-SS316	0.736	0.361	23
1.5	4	1	µg	LBK	p-SS316	0.979	0.3	24
1.5	4	1	µg	LBK	p-SS316	0.834	0.383	25
2.1	1	1	µg	LBK	MIT-LIS	1.214	0.393	1
2.1	1	1	µg	LBK	MIT-LIS	0.887	0.385	2
2.1	1	1	µg	LBK	MIT-LIS	0.81	0.382	3
2.1	1	1	µg	LBK	MIT-LIS	0.768	0.317	4
2.1	1	1	µg	LBK	MIT-LIS	0.674	0.327	5
2.1	1	1	µg	LBK	MIT-LIS	1.094	0.36	6
2.1	1	1	µg	LBK	MIT-LIS	0.939	0.397	7
2.1	1	1	µg	LBK	MIT-LIS	0.583	0.36	8
2.1	1	1	µg	LBK	MIT-LIS	0.806	0.344	9
2.1	1	1	µg	LBK	MIT-LIS	0.794	0.379	10
2.1	1	1	µg	LBK	MIT-LIS	0.954	0.368	11
2.1	1	1	µg	LBK	MIT-LIS	0.849	0.297	12
2.1	1	1	µg	LBK	MIT-LIS	0.954	0.346	13
2.1	1	1	µg	LBK	MIT-LIS	0.936	0.356	14
2.1	1	1	µg	LBK	MIT-LIS	0.805	0.302	15
2.1	1	1	µg	LBK	MIT-LIS	1.013	0.36	16
2.1	1	1	µg	LBK	MIT-LIS	1.146	0.302	17
2.1	1	1	µg	LBK	MIT-LIS	0.77	0.351	18
2.1	1	1	µg	LBK	MIT-LIS	0.661	0.322	19
2.1	1	1	µg	LBK	MIT-LIS	0.881	0.368	20
2.1	1	1	µg	LBK	MIT-LIS	0.876	0.346	21
2.1	1	1	µg	LBK	MIT-LIS	0.869	0.343	22
2.1	1	1	µg	LBK	MIT-LIS	0.825	0.325	23
2.1	1	1	µg	LBK	MIT-LIS	1.016	0.34	24
2.1	1	1	µg	LBK	MIT-LIS	0.784	0.28	25
2.1	2	1	µg	LBK	MIT-LIS	0.629	0.414	1
2.1	2	1	µg	LBK	MIT-LIS	0.782	0.368	2
2.1	2	1	µg	LBK	MIT-LIS	0.769	0.331	3
2.1	2	1	µg	LBK	MIT-LIS	0.787	0.335	4
2.1	2	1	µg	LBK	MIT-LIS	0.773	0.309	5
2.1	2	1	µg	LBK	MIT-LIS	0.734	0.251	6
2.1	2	1	µg	LBK	MIT-LIS	0.743	0.37	7
2.1	2	1	µg	LBK	MIT-LIS	0.764	0.322	8
2.1	2	1	µg	LBK	MIT-LIS	0.938	0.323	9
2.1	2	1	µg	LBK	MIT-LIS	0.698	0.391	10
2.1	2	1	µg	LBK	MIT-LIS	0.529	0.317	11
2.1	2	1	µg	LBK	MIT-LIS	0.823	0.356	12
2.1	2	1	µg	LBK	MIT-LIS	0.903	0.376	13
2.1	2	1	µg	LBK	MIT-LIS	0.719	0.292	14
2.1	2	1	µg	LBK	MIT-LIS	0.71	0.296	15
2.1	2	1	µg	LBK	MIT-LIS	0.875	0.345	16
2.1	2	1	µg	LBK	MIT-LIS	0.789	0.385	17
2.1	2	1	µg	LBK	MIT-LIS	0.971	0.355	18
2.1	2	1	µg	LBK	MIT-LIS	0.867	0.296	19
2.1	2	1	µg	LBK	MIT-LIS	0.771	0.368	20
2.1	2	1	µg	LBK	MIT-LIS	0.686	0.404	21
2.1	2	1	µg	LBK	MIT-LIS	0.64	0.309	22

2.1	2	1	µg	LBK	MIT-LIS	0.798	0.371	23
2.1	2	1	µg	LBK	MIT-LIS	0.953	0.345	24
2.1	2	1	µg	LBK	MIT-LIS	0.745	0.291	25
2.1	3	1	µg	LBK	MIT-LIS	1.105	0.36	1
2.1	3	1	µg	LBK	MIT-LIS	0.778	0.355	2
2.1	3	1	µg	LBK	MIT-LIS	0.749	0.413	3
2.1	3	1	µg	LBK	MIT-LIS	0.683	0.302	4
2.1	3	1	µg	LBK	MIT-LIS	0.688	0.302	5
2.1	3	1	µg	LBK	MIT-LIS	0.929	0.32	6
2.1	3	1	µg	LBK	MIT-LIS	0.674	0.32	7
2.1	3	1	µg	LBK	MIT-LIS	0.728	0.355	8
2.1	3	1	µg	LBK	MIT-LIS	0.71	0.25	9
2.1	3	1	µg	LBK	MIT-LIS	0.769	0.296	10
2.1	3	1	µg	LBK	MIT-LIS	0.847	0.424	11
2.1	3	1	µg	LBK	MIT-LIS	0.637	0.281	12
2.1	3	1	µg	LBK	MIT-LIS	0.703	0.31	13
2.1	3	1	µg	LBK	MIT-LIS	0.529	0.393	14
2.1	3	1	µg	LBK	MIT-LIS	0.636	0.327	15
2.1	3	1	µg	LBK	MIT-LIS	0.629	0.355	16
2.1	3	1	µg	LBK	MIT-LIS	0.781	0.463	17
2.1	3	1	µg	LBK	MIT-LIS	0.712	0.299	18
2.1	3	1	µg	LBK	MIT-LIS	0.932	0.337	19
2.1	3	1	µg	LBK	MIT-LIS	1.006	0.397	20
2.1	3	1	µg	LBK	MIT-LIS	0.704	0.207	21
2.1	3	1	µg	LBK	MIT-LIS	0.543	0.376	22
2.1	3	1	µg	LBK	MIT-LIS	0.603	0.32	23
2.1	3	1	µg	LBK	MIT-LIS	0.806	0.36	24
2.1	3	1	µg	LBK	MIT-LIS	0.764	0.319	25
2.1	4	1	µg	LBK	MIT-LIS	0.629	0.411	1
2.1	4	1	µg	LBK	MIT-LIS	0.926	0.359	2
2.1	4	1	µg	LBK	MIT-LIS	0.767	0.331	3
2.1	4	1	µg	LBK	MIT-LIS	0.883	0.406	4
2.1	4	1	µg	LBK	MIT-LIS	0.734	0.343	5
2.1	4	1	µg	LBK	MIT-LIS	0.583	0.331	6
2.1	4	1	µg	LBK	MIT-LIS	1.014	0.351	7
2.1	4	1	µg	LBK	MIT-LIS	0.657	0.403	8
2.1	4	1	µg	LBK	MIT-LIS	0.958	0.319	9
2.1	4	1	µg	LBK	MIT-LIS	0.801	0.386	10
2.1	4	1	µg	LBK	MIT-LIS	0.855	0.348	11
2.1	4	1	µg	LBK	MIT-LIS	1.136	0.355	12
2.1	4	1	µg	LBK	MIT-LIS	0.71	0.323	13
2.1	4	1	µg	LBK	MIT-LIS	0.571	0.294	14
2.1	4	1	µg	LBK	MIT-LIS	0.862	0.349	15
2.1	4	1	µg	LBK	MIT-LIS	0.674	0.344	16
2.1	4	1	µg	LBK	MIT-LIS	0.603	0.393	17
2.1	4	1	µg	LBK	MIT-LIS	0.988	0.355	18
2.1	4	1	µg	LBK	MIT-LIS	0.615	0.379	19
2.1	4	1	µg	LBK	MIT-LIS	0.741	0.345	20
2.1	4	1	µg	LBK	MIT-LIS	0.771	0.403	21
2.1	4	1	µg	LBK	MIT-LIS	0.948	0.31	22

2.1	4	1	µg	LBK	MIT-LIS	0.688	0.285	23
2.1	4	1	µg	LBK	MIT-LIS	0.785	0.345	24
2.1	4	1	µg	LBK	MIT-LIS	0.712	0.323	25
7.1	1	2	µg	LBK	SS316	1.111	0.347	1
7.1	1	2	µg	LBK	SS316	1.082	0.283	2
7.1	1	2	µg	LBK	SS316	0.923	0.302	3
7.1	1	2	µg	LBK	SS316	0.991	0.295	4
7.1	1	2	µg	LBK	SS316	1.161	0.348	5
7.1	1	2	µg	LBK	SS316	0.904	0.306	6
7.1	1	2	µg	LBK	SS316	0.936	0.369	7
7.1	1	2	µg	LBK	SS316	0.944	0.347	8
7.1	1	2	µg	LBK	SS316	0.94	0.292	9
7.1	1	2	µg	LBK	SS316	0.91	0.346	10
7.1	1	2	µg	LBK	SS316	1.097	0.299	11
7.1	1	2	µg	LBK	SS316	1.179	0.32	12
7.1	1	2	µg	LBK	SS316	1.242	0.345	13
7.1	1	2	µg	LBK	SS316	0.929	0.337	14
7.1	1	2	µg	LBK	SS316	0.995	0.391	15
7.1	1	2	µg	LBK	SS316	1.114	0.303	16
7.1	1	2	µg	LBK	SS316	1.09	0.325	17
7.1	1	2	µg	LBK	SS316	1.026	0.332	18
7.1	1	2	µg	LBK	SS316	0.979	0.388	19
7.1	1	2	µg	LBK	SS316	1.274	0.305	20
7.1	1	2	µg	LBK	SS316	0.885	0.363	21
7.1	1	2	µg	LBK	SS316	0.913	0.288	22
7.1	1	2	µg	LBK	SS316	1.107	0.317	23
7.1	1	2	µg	LBK	SS316	0.812	0.265	24
7.1	1	2	µg	LBK	SS316	0.77	0.333	25
7.1	2	2	µg	LBK	SS316	0.774	0.317	1
7.1	2	2	µg	LBK	SS316	0.825	0.294	2
7.1	2	2	µg	LBK	SS316	1.122	0.337	3
7.1	2	2	µg	LBK	SS316	0.913	0.368	4
7.1	2	2	µg	LBK	SS316	0.652	0.306	5
7.1	2	2	µg	LBK	SS316	0.982	0.299	6
7.1	2	2	µg	LBK	SS316	0.759	0.236	7
7.1	2	2	µg	LBK	SS316	0.767	0.304	8
7.1	2	2	µg	LBK	SS316	0.763	0.366	9
7.1	2	2	µg	LBK	SS316	0.893	0.349	10
7.1	2	2	µg	LBK	SS316	1.125	0.322	11
7.1	2	2	µg	LBK	SS316	0.785	0.333	12
7.1	2	2	µg	LBK	SS316	1.323	0.347	13
7.1	2	2	µg	LBK	SS316	0.971	0.254	14
7.1	2	2	µg	LBK	SS316	1.215	0.273	15
7.1	2	2	µg	LBK	SS316	1.049	0.269	16
7.1	2	2	µg	LBK	SS316	1.155	0.336	17
7.1	2	2	µg	LBK	SS316	0.868	0.274	18
7.1	2	2	µg	LBK	SS316	1.015	0.329	19
7.1	2	2	µg	LBK	SS316	0.769	0.297	20
7.1	2	2	µg	LBK	SS316	0.712	0.31	21
7.1	2	2	µg	LBK	SS316	1.523	0.26	22

7.1	2	2	µg	LBK	SS316	0.849	0.266	23
7.1	2	2	µg	LBK	SS316	0.857	0.336	24
7.1	2	2	µg	LBK	SS316	0.968	0.3	25
7.1	3	2	µg	LBK	SS316	0.937	0.336	1
7.1	3	2	µg	LBK	SS316	0.998	0.327	2
7.1	3	2	µg	LBK	SS316	0.796	0.331	3
7.1	3	2	µg	LBK	SS316	0.907	0.32	4
7.1	3	2	µg	LBK	SS316	1.079	0.303	5
7.1	3	2	µg	LBK	SS316	1.068	0.289	6
7.1	3	2	µg	LBK	SS316	1.119	0.249	7
7.1	3	2	µg	LBK	SS316	0.728	0.366	8
7.1	3	2	µg	LBK	SS316	0.757	0.342	9
7.1	3	2	µg	LBK	SS316	0.8	0.306	10
7.1	3	2	µg	LBK	SS316	0.952	0.357	11
7.1	3	2	µg	LBK	SS316	1.177	0.359	12
7.1	3	2	µg	LBK	SS316	0.606	0.344	13
7.1	3	2	µg	LBK	SS316	0.885	0.369	14
7.1	3	2	µg	LBK	SS316	1.245	0.31	15
7.1	3	2	µg	LBK	SS316	0.86	0.251	16
7.1	3	2	µg	LBK	SS316	0.656	0.353	17
7.1	3	2	µg	LBK	SS316	0.859	0.326	18
7.1	3	2	µg	LBK	SS316	0.928	0.377	19
7.1	3	2	µg	LBK	SS316	1.075	0.392	20
7.1	3	2	µg	LBK	SS316	0.862	0.341	21
7.1	3	2	µg	LBK	SS316	1.049	0.381	22
7.1	3	2	µg	LBK	SS316	1.082	0.367	23
7.1	3	2	µg	LBK	SS316	0.912	0.339	24
7.1	3	2	µg	LBK	SS316	1.22	0.305	25
7.1	4	2	µg	LBK	SS316	1.023	0.348	1
7.1	4	2	µg	LBK	SS316	1.141	0.277	2
7.1	4	2	µg	LBK	SS316	0.83	0.362	3
7.1	4	2	µg	LBK	SS316	0.96	0.327	4
7.1	4	2	µg	LBK	SS316	0.64	0.344	5
7.1	4	2	µg	LBK	SS316	1.131	0.378	6
7.1	4	2	µg	LBK	SS316	0.949	0.334	7
7.1	4	2	µg	LBK	SS316	1.136	0.312	8
7.1	4	2	µg	LBK	SS316	0.898	0.354	9
7.1	4	2	µg	LBK	SS316	1.043	0.324	10
7.1	4	2	µg	LBK	SS316	0.849	0.377	11
7.1	4	2	µg	LBK	SS316	0.896	0.288	12
7.1	4	2	µg	LBK	SS316	0.843	0.321	13
7.1	4	2	µg	LBK	SS316	0.717	0.351	14
7.1	4	2	µg	LBK	SS316	1.103	0.317	15
7.1	4	2	µg	LBK	SS316	1.448	0.348	16
7.1	4	2	µg	LBK	SS316	0.945	0.318	17
7.1	4	2	µg	LBK	SS316	1.207	0.321	18
7.1	4	2	µg	LBK	SS316	0.821	0.329	19
7.1	4	2	µg	LBK	SS316	0.85	0.331	20
7.1	4	2	µg	LBK	SS316	1.201	0.302	21
7.1	4	2	µg	LBK	SS316	0.981	0.327	22

7.1	4	2	µg	LBK	SS316	1.36	0.308	23
7.1	4	2	µg	LBK	SS316	1.182	0.312	24
7.1	4	2	µg	LBK	SS316	1.263	0.317	25
7.5	1	2	µg	LBK	p-SS316	0.924	0.288	1
7.5	1	2	µg	LBK	p-SS316	1.01	0.32	2
7.5	1	2	µg	LBK	p-SS316	1.06	0.286	3
7.5	1	2	µg	LBK	p-SS316	1.003	0.317	4
7.5	1	2	µg	LBK	p-SS316	0.95	0.316	5
7.5	1	2	µg	LBK	p-SS316	1.021	0.252	6
7.5	1	2	µg	LBK	p-SS316	0.745	0.283	7
7.5	1	2	µg	LBK	p-SS316	0.725	0.254	8
7.5	1	2	µg	LBK	p-SS316	0.779	0.254	9
7.5	1	2	µg	LBK	p-SS316	0.901	0.293	10
7.5	1	2	µg	LBK	p-SS316	1.269	0.326	11
7.5	1	2	µg	LBK	p-SS316	0.73	0.295	12
7.5	1	2	µg	LBK	p-SS316	0.861	0.363	13
7.5	1	2	µg	LBK	p-SS316	0.876	0.356	14
7.5	1	2	µg	LBK	p-SS316	0.946	0.277	15
7.5	1	2	µg	LBK	p-SS316	0.822	0.235	16
7.5	1	2	µg	LBK	p-SS316	0.795	0.298	17
7.5	1	2	µg	LBK	p-SS316	0.967	0.245	18
7.5	1	2	µg	LBK	p-SS316	0.76	0.264	19
7.5	1	2	µg	LBK	p-SS316	1.18	0.31	20
7.5	1	2	µg	LBK	p-SS316	1.244	0.28	21
7.5	1	2	µg	LBK	p-SS316	0.762	0.302	22
7.5	1	2	µg	LBK	p-SS316	0.991	0.298	23
7.5	1	2	µg	LBK	p-SS316	1.151	0.31	24
7.5	1	2	µg	LBK	p-SS316	0.839	0.287	25
7.5	2	2	µg	LBK	p-SS316	0.811	0.284	1
7.5	2	2	µg	LBK	p-SS316	1.043	0.381	2
7.5	2	2	µg	LBK	p-SS316	0.794	0.272	3
7.5	2	2	µg	LBK	p-SS316	0.779	0.304	4
7.5	2	2	µg	LBK	p-SS316	1.169	0.34	5
7.5	2	2	µg	LBK	p-SS316	0.974	0.272	6
7.5	2	2	µg	LBK	p-SS316	0.943	0.362	7
7.5	2	2	µg	LBK	p-SS316	0.851	0.29	8
7.5	2	2	µg	LBK	p-SS316	1.196	0.256	9
7.5	2	2	µg	LBK	p-SS316	0.92	0.334	10
7.5	2	2	µg	LBK	p-SS316	0.895	0.318	11
7.5	2	2	µg	LBK	p-SS316	1.078	0.346	12
7.5	2	2	µg	LBK	p-SS316	0.878	0.304	13
7.5	2	2	µg	LBK	p-SS316	0.913	0.344	14
7.5	2	2	µg	LBK	p-SS316	1.115	0.332	15
7.5	2	2	µg	LBK	p-SS316	0.923	0.329	16
7.5	2	2	µg	LBK	p-SS316	0.965	0.314	17
7.5	2	2	µg	LBK	p-SS316	0.939	0.281	18
7.5	2	2	µg	LBK	p-SS316	0.751	0.391	19
7.5	2	2	µg	LBK	p-SS316	1.028	0.3	20
7.5	2	2	µg	LBK	p-SS316	0.777	0.321	21
7.5	2	2	µg	LBK	p-SS316	0.865	0.304	22

7.5	2	2	µg	LBK	p-SS316	0.707	0.344	23
7.5	2	2	µg	LBK	p-SS316	0.616	0.321	24
7.5	2	2	µg	LBK	p-SS316	0.825	0.298	25
7.5	3	2	µg	LBK	p-SS316	0.896	0.345	1
7.5	3	2	µg	LBK	p-SS316	0.728	0.343	2
7.5	3	2	µg	LBK	p-SS316	0.913	0.327	3
7.5	3	2	µg	LBK	p-SS316	0.86	0.319	4
7.5	3	2	µg	LBK	p-SS316	0.737	0.356	5
7.5	3	2	µg	LBK	p-SS316	0.821	0.283	6
7.5	3	2	µg	LBK	p-SS316	0.823	0.356	7
7.5	3	2	µg	LBK	p-SS316	0.912	0.308	8
7.5	3	2	µg	LBK	p-SS316	0.835	0.368	9
7.5	3	2	µg	LBK	p-SS316	0.81	0.348	10
7.5	3	2	µg	LBK	p-SS316	0.828	0.374	11
7.5	3	2	µg	LBK	p-SS316	0.966	0.371	12
7.5	3	2	µg	LBK	p-SS316	1.089	0.283	13
7.5	3	2	µg	LBK	p-SS316	0.823	0.35	14
7.5	3	2	µg	LBK	p-SS316	1.066	0.276	15
7.5	3	2	µg	LBK	p-SS316	0.981	0.267	16
7.5	3	2	µg	LBK	p-SS316	0.764	0.331	17
7.5	3	2	µg	LBK	p-SS316	0.846	0.333	18
7.5	3	2	µg	LBK	p-SS316	0.724	0.288	19
7.5	3	2	µg	LBK	p-SS316	0.995	0.319	20
7.5	3	2	µg	LBK	p-SS316	0.714	0.309	21
7.5	3	2	µg	LBK	p-SS316	0.761	0.26	22
7.5	3	2	µg	LBK	p-SS316	0.777	0.28	23
7.5	3	2	µg	LBK	p-SS316	0.764	0.315	24
7.5	3	2	µg	LBK	p-SS316	0.861	0.377	25
7.5	4	2	µg	LBK	p-SS316	0.925	0.333	1
7.5	4	2	µg	LBK	p-SS316	1.12	0.366	2
7.5	4	2	µg	LBK	p-SS316	1.07	0.352	3
7.5	4	2	µg	LBK	p-SS316	1.341	0.283	4
7.5	4	2	µg	LBK	p-SS316	0.954	0.298	5
7.5	4	2	µg	LBK	p-SS316	0.863	0.317	6
7.5	4	2	µg	LBK	p-SS316	1.204	0.344	7
7.5	4	2	µg	LBK	p-SS316	1.365	0.348	8
7.5	4	2	µg	LBK	p-SS316	0.888	0.343	9
7.5	4	2	µg	LBK	p-SS316	0.875	0.403	10
7.5	4	2	µg	LBK	p-SS316	1.176	0.29	11
7.5	4	2	µg	LBK	p-SS316	1.007	0.349	12
7.5	4	2	µg	LBK	p-SS316	0.948	0.336	13
7.5	4	2	µg	LBK	p-SS316	0.995	0.368	14
7.5	4	2	µg	LBK	p-SS316	1.187	0.309	15
7.5	4	2	µg	LBK	p-SS316	1.366	0.352	16
7.5	4	2	µg	LBK	p-SS316	0.863	0.349	17
7.5	4	2	µg	LBK	p-SS316	1.395	0.326	18
7.5	4	2	µg	LBK	p-SS316	1.233	0.349	19
7.5	4	2	µg	LBK	p-SS316	1.262	0.321	20
7.5	4	2	µg	LBK	p-SS316	1.162	0.33	21
7.5	4	2	µg	LBK	p-SS316	0.949	0.31	22

7.5	4	2	µg	LBK	p-SS316	0.773	0.338	23
7.5	4	2	µg	LBK	p-SS316	1.329	0.34	24
7.5	4	2	µg	LBK	p-SS316	0.784	0.338	25
8.1	1	2	µg	LBK	MIT-LIS	0.81	0.282	1
8.1	1	2	µg	LBK	MIT-LIS	0.839	0.287	2
8.1	1	2	µg	LBK	MIT-LIS	0.717	0.305	3
8.1	1	2	µg	LBK	MIT-LIS	0.876	0.232	4
8.1	1	2	µg	LBK	MIT-LIS	0.713	0.386	5
8.1	1	2	µg	LBK	MIT-LIS	0.71	0.287	6
8.1	1	2	µg	LBK	MIT-LIS	0.702	0.253	7
8.1	1	2	µg	LBK	MIT-LIS	0.937	0.255	8
8.1	1	2	µg	LBK	MIT-LIS	0.925	0.304	9
8.1	1	2	µg	LBK	MIT-LIS	0.965	0.333	10
8.1	1	2	µg	LBK	MIT-LIS	0.934	0.361	11
8.1	1	2	µg	LBK	MIT-LIS	0.96	0.305	12
8.1	1	2	µg	LBK	MIT-LIS	1.087	0.324	13
8.1	1	2	µg	LBK	MIT-LIS	1.046	0.275	14
8.1	1	2	µg	LBK	MIT-LIS	0.803	0.351	15
8.1	1	2	µg	LBK	MIT-LIS	0.974	0.375	16
8.1	1	2	µg	LBK	MIT-LIS	0.688	0.284	17
8.1	1	2	µg	LBK	MIT-LIS	0.779	0.315	18
8.1	1	2	µg	LBK	MIT-LIS	1.101	0.345	19
8.1	1	2	µg	LBK	MIT-LIS	0.76	0.315	20
8.1	1	2	µg	LBK	MIT-LIS	0.724	0.254	21
8.1	1	2	µg	LBK	MIT-LIS	0.672	0.332	22
8.1	1	2	µg	LBK	MIT-LIS	0.983	0.278	23
8.1	1	2	µg	LBK	MIT-LIS	0.79	0.297	24
8.1	1	2	µg	LBK	MIT-LIS	1.116	0.388	25
8.1	2	2	µg	LBK	MIT-LIS	1.046	0.39	1
8.1	2	2	µg	LBK	MIT-LIS	0.885	0.355	2
8.1	2	2	µg	LBK	MIT-LIS	0.811	0.292	3
8.1	2	2	µg	LBK	MIT-LIS	0.843	0.299	4
8.1	2	2	µg	LBK	MIT-LIS	0.681	0.313	5
8.1	2	2	µg	LBK	MIT-LIS	0.639	0.317	6
8.1	2	2	µg	LBK	MIT-LIS	0.83	0.335	7
8.1	2	2	µg	LBK	MIT-LIS	0.757	0.375	8
8.1	2	2	µg	LBK	MIT-LIS	0.882	0.305	9
8.1	2	2	µg	LBK	MIT-LIS	0.774	0.378	10
8.1	2	2	µg	LBK	MIT-LIS	1.205	0.323	11
8.1	2	2	µg	LBK	MIT-LIS	0.819	0.244	12
8.1	2	2	µg	LBK	MIT-LIS	0.779	0.299	13
8.1	2	2	µg	LBK	MIT-LIS	1.417	0.353	14
8.1	2	2	µg	LBK	MIT-LIS	0.95	0.276	15
8.1	2	2	µg	LBK	MIT-LIS	1.058	0.395	16
8.1	2	2	µg	LBK	MIT-LIS	0.946	0.305	17
8.1	2	2	µg	LBK	MIT-LIS	1.036	0.308	18
8.1	2	2	µg	LBK	MIT-LIS	0.727	0.393	19
8.1	2	2	µg	LBK	MIT-LIS	0.979	0.395	20
8.1	2	2	µg	LBK	MIT-LIS	0.976	0.265	21
8.1	2	2	µg	LBK	MIT-LIS	0.761	0.312	22

8.1	2	2	µg	LBK	MIT-LIS	0.733	0.361	23
8.1	2	2	µg	LBK	MIT-LIS	0.702	0.321	24
8.1	2	2	µg	LBK	MIT-LIS	1.141	0.39	25
8.1	3	2	µg	LBK	MIT-LIS	0.88	0.341	1
8.1	3	2	µg	LBK	MIT-LIS	0.653	0.356	2
8.1	3	2	µg	LBK	MIT-LIS	1.012	0.294	3
8.1	3	2	µg	LBK	MIT-LIS	0.776	0.401	4
8.1	3	2	µg	LBK	MIT-LIS	0.709	0.312	5
8.1	3	2	µg	LBK	MIT-LIS	0.722	0.348	6
8.1	3	2	µg	LBK	MIT-LIS	0.624	0.289	7
8.1	3	2	µg	LBK	MIT-LIS	0.604	0.288	8
8.1	3	2	µg	LBK	MIT-LIS	0.643	0.257	9
8.1	3	2	µg	LBK	MIT-LIS	0.876	0.357	10
8.1	3	2	µg	LBK	MIT-LIS	0.776	0.281	11
8.1	3	2	µg	LBK	MIT-LIS	0.897	0.294	12
8.1	3	2	µg	LBK	MIT-LIS	0.661	0.296	13
8.1	3	2	µg	LBK	MIT-LIS	0.894	0.339	14
8.1	3	2	µg	LBK	MIT-LIS	0.721	0.245	15
8.1	3	2	µg	LBK	MIT-LIS	0.806	0.365	16
8.1	3	2	µg	LBK	MIT-LIS	0.713	0.344	17
8.1	3	2	µg	LBK	MIT-LIS	0.788	0.411	18
8.1	3	2	µg	LBK	MIT-LIS	0.943	0.315	19
8.1	3	2	µg	LBK	MIT-LIS	0.677	0.334	20
8.1	3	2	µg	LBK	MIT-LIS	0.802	0.349	21
8.1	3	2	µg	LBK	MIT-LIS	0.762	0.341	22
8.1	3	2	µg	LBK	MIT-LIS	0.684	0.289	23
8.1	3	2	µg	LBK	MIT-LIS	0.817	0.341	24
8.1	3	2	µg	LBK	MIT-LIS	0.743	0.273	25
8.1	4	2	µg	LBK	MIT-LIS	0.796	0.279	1
8.1	4	2	µg	LBK	MIT-LIS	0.721	0.313	2
8.1	4	2	µg	LBK	MIT-LIS	0.596	0.25	3
8.1	4	2	µg	LBK	MIT-LIS	0.73	0.286	4
8.1	4	2	µg	LBK	MIT-LIS	0.929	0.274	5
8.1	4	2	µg	LBK	MIT-LIS	0.884	0.334	6
8.1	4	2	µg	LBK	MIT-LIS	0.687	0.32	7
8.1	4	2	µg	LBK	MIT-LIS	0.763	0.302	8
8.1	4	2	µg	LBK	MIT-LIS	0.884	0.335	9
8.1	4	2	µg	LBK	MIT-LIS	0.693	0.329	10
8.1	4	2	µg	LBK	MIT-LIS	0.841	0.309	11
8.1	4	2	µg	LBK	MIT-LIS	0.682	0.311	12
8.1	4	2	µg	LBK	MIT-LIS	0.797	0.312	13
8.1	4	2	µg	LBK	MIT-LIS	0.828	0.333	14
8.1	4	2	µg	LBK	MIT-LIS	0.757	0.287	15
8.1	4	2	µg	LBK	MIT-LIS	0.738	0.285	16
8.1	4	2	µg	LBK	MIT-LIS	0.816	0.373	17
8.1	4	2	µg	LBK	MIT-LIS	0.761	0.348	18
8.1	4	2	µg	LBK	MIT-LIS	0.822	0.403	19
8.1	4	2	µg	LBK	MIT-LIS	0.909	0.335	20
8.1	4	2	µg	LBK	MIT-LIS	0.837	0.369	21
8.1	4	2	µg	LBK	MIT-LIS	0.877	0.398	22

8.1	4	2	µg	LBK	MIT-LIS	0.856	0.267	23
8.1	4	2	µg	LBK	MIT-LIS	0.775	0.315	24
8.1	4	2	µg	LBK	MIT-LIS	1.009	0.367	25
13.1	1	3	µg	LBK	SS316	0.8371875	0.292125	1
13.1	1	3	µg	LBK	SS316	1.132875	0.306375	2
13.1	1	3	µg	LBK	SS316	1.5675	0.2386875	3
13.1	1	3	µg	LBK	SS316	0.8015625	0.2956875	4
13.1	1	3	µg	LBK	SS316	0.92625	0.2671875	5
13.1	1	3	µg	LBK	SS316	1.332375	0.220875	6
13.1	1	3	µg	LBK	SS316	1.047375	0.228	7
13.1	1	3	µg	LBK	SS316	1.2148125	0.3455625	8
13.1	1	3	µg	LBK	SS316	1.3003125	0.292125	9
13.1	1	3	µg	LBK	SS316	0.833625	0.2814375	10
13.1	1	3	µg	LBK	SS316	0.933375	0.3241875	11
13.1	1	3	µg	LBK	SS316	0.798	0.3241875	12
13.1	1	3	µg	LBK	SS316	1.118625	0.29925	13
13.1	1	3	µg	LBK	SS316	1.0865625	0.235125	14
13.1	1	3	µg	LBK	SS316	0.976125	0.3705	15
13.1	1	3	µg	LBK	SS316	0.904875	0.2814375	16
13.1	1	3	µg	LBK	SS316	0.89775	0.2814375	17
13.1	1	3	µg	LBK	SS316	0.9654375	0.3455625	18
13.1	1	3	µg	LBK	SS316	0.9654375	0.29925	19
13.1	1	3	µg	LBK	SS316	1.004625	0.3099375	20
13.1	1	3	µg	LBK	SS316	1.43925	0.29925	21
13.1	1	3	µg	LBK	SS316	1.2290625	0.32775	22
13.1	1	3	µg	LBK	SS316	1.49625	0.2743125	23
13.1	1	3	µg	LBK	SS316	1.4143125	0.32775	24
13.1	1	3	µg	LBK	SS316	1.353	0.324	25
13.1	2	3	µg	LBK	SS316	1.066	0.301	1
13.1	2	3	µg	LBK	SS316	1.022	0.239	2
13.1	2	3	µg	LBK	SS316	1.511	0.266	3
13.1	2	3	µg	LBK	SS316	0.954	0.39	4
13.1	2	3	µg	LBK	SS316	1.307	0.31	5
13.1	2	3	µg	LBK	SS316	1.285	0.322	6
13.1	2	3	µg	LBK	SS316	1.019	0.264	7
13.1	2	3	µg	LBK	SS316	1.002	0.311	8
13.1	2	3	µg	LBK	SS316	1.396	0.301	9
13.1	2	3	µg	LBK	SS316	0.838	0.391	10
13.1	2	3	µg	LBK	SS316	1.335	0.301	11
13.1	2	3	µg	LBK	SS316	1.378	0.257	12
13.1	2	3	µg	LBK	SS316	1.065	0.278	13
13.1	2	3	µg	LBK	SS316	0.965	0.294	14
13.1	2	3	µg	LBK	SS316	0.94	0.34	15
13.1	2	3	µg	LBK	SS316	1.21	0.284	16
13.1	2	3	µg	LBK	SS316	0.931	0.328	17
13.1	2	3	µg	LBK	SS316	1.056	0.342	18
13.1	2	3	µg	LBK	SS316	1.04	0.283	19
13.1	2	3	µg	LBK	SS316	1.423	0.273	20
13.1	2	3	µg	LBK	SS316	1.025	0.32	21
13.1	2	3	µg	LBK	SS316	0.811	0.31	22

13.1	2	3	µg	LBK	SS316	1.016	0.327	23
13.1	2	3	µg	LBK	SS316	1.133	0.265	24
13.1	2	3	µg	LBK	SS316	1.45	0.345	25
13.1	3	3	µg	LBK	SS316	0.777	0.295	1
13.1	3	3	µg	LBK	SS316	0.92	0.295	2
13.1	3	3	µg	LBK	SS316	1.118	0.414	3
13.1	3	3	µg	LBK	SS316	1.212	0.303	4
13.1	3	3	µg	LBK	SS316	1.268	0.342	5
13.1	3	3	µg	LBK	SS316	1.299	0.32	6
13.1	3	3	µg	LBK	SS316	1.202	0.274	7
13.1	3	3	µg	LBK	SS316	1.569	0.269	8
13.1	3	3	µg	LBK	SS316	1.109	0.294	9
13.1	3	3	µg	LBK	SS316	0.939	0.346	10
13.1	3	3	µg	LBK	SS316	0.948	0.274	11
13.1	3	3	µg	LBK	SS316	0.785	0.374	12
13.1	3	3	µg	LBK	SS316	1.001	0.336	13
13.1	3	3	µg	LBK	SS316	1.692	0.276	14
13.1	3	3	µg	LBK	SS316	0.716	0.266	15
13.1	3	3	µg	LBK	SS316	0.858	0.271	16
13.1	3	3	µg	LBK	SS316	1.527	0.233	17
13.1	3	3	µg	LBK	SS316	0.665	0.253	18
13.1	3	3	µg	LBK	SS316	0.758	0.31	19
13.1	3	3	µg	LBK	SS316	1.613	0.376	20
13.1	3	3	µg	LBK	SS316	0.828	0.373	21
13.1	3	3	µg	LBK	SS316	1.237	0.345	22
13.1	3	3	µg	LBK	SS316	1.152	0.325	23
13.1	3	3	µg	LBK	SS316	0.939	0.219	24
13.1	3	3	µg	LBK	SS316	1.423	0.328	25
13.1	4	3	µg	LBK	SS316	0.841	0.252	1
13.1	4	3	µg	LBK	SS316	1.206	0.319	2
13.1	4	3	µg	LBK	SS316	1.142	0.252	3
13.1	4	3	µg	LBK	SS316	0.802	0.303	4
13.1	4	3	µg	LBK	SS316	0.946	0.27	5
13.1	4	3	µg	LBK	SS316	1.043	0.278	6
13.1	4	3	µg	LBK	SS316	1.291	0.275	7
13.1	4	3	µg	LBK	SS316	1.356	0.295	8
13.1	4	3	µg	LBK	SS316	1.586	0.248	9
13.1	4	3	µg	LBK	SS316	1.069	0.231	10
13.1	4	3	µg	LBK	SS316	1.302	0.23	11
13.1	4	3	µg	LBK	SS316	1.05	0.246	12
13.1	4	3	µg	LBK	SS316	1.129	0.301	13
13.1	4	3	µg	LBK	SS316	0.882	0.256	14
13.1	4	3	µg	LBK	SS316	0.817	0.195	15
13.1	4	3	µg	LBK	SS316	0.965	0.254	16
13.1	4	3	µg	LBK	SS316	0.981	0.332	17
13.1	4	3	µg	LBK	SS316	0.919	0.289	18
13.1	4	3	µg	LBK	SS316	1.285	0.297	19
13.1	4	3	µg	LBK	SS316	0.984	0.316	20
13.1	4	3	µg	LBK	SS316	0.865	0.345	21
13.1	4	3	µg	LBK	SS316	0.973	0.238	22

13.1	4	3	µg	LBK	SS316	1.304	0.271	23
13.1	4	3	µg	LBK	SS316	0.791	0.291	24
13.1	4	3	µg	LBK	SS316	0.959	0.233	25
13.5	1	3	µg	LBK	p-SS316	1.145	0.326	1
13.5	1	3	µg	LBK	p-SS316	0.94	0.288	2
13.5	1	3	µg	LBK	p-SS316	0.921	0.279	3
13.5	1	3	µg	LBK	p-SS316	1.102	0.354	4
13.5	1	3	µg	LBK	p-SS316	1.199	0.286	5
13.5	1	3	µg	LBK	p-SS316	0.682	0.278	6
13.5	1	3	µg	LBK	p-SS316	1.035	0.258	7
13.5	1	3	µg	LBK	p-SS316	1.477	0.308	8
13.5	1	3	µg	LBK	p-SS316	0.791	0.335	9
13.5	1	3	µg	LBK	p-SS316	1	0.309	10
13.5	1	3	µg	LBK	p-SS316	1.081	0.329	11
13.5	1	3	µg	LBK	p-SS316	1.11	0.314	12
13.5	1	3	µg	LBK	p-SS316	0.973	0.302	13
13.5	1	3	µg	LBK	p-SS316	0.942	0.251	14
13.5	1	3	µg	LBK	p-SS316	1.308	0.337	15
13.5	1	3	µg	LBK	p-SS316	1.111	0.323	16
13.5	1	3	µg	LBK	p-SS316	0.655	0.349	17
13.5	1	3	µg	LBK	p-SS316	1.121	0.282	18
13.5	1	3	µg	LBK	p-SS316	1.189	0.334	19
13.5	1	3	µg	LBK	p-SS316	1.252	0.283	20
13.5	1	3	µg	LBK	p-SS316	0.899	0.357	21
13.5	1	3	µg	LBK	p-SS316	0.877	0.301	22
13.5	1	3	µg	LBK	p-SS316	1.119	0.282	23
13.5	1	3	µg	LBK	p-SS316	1.164	0.323	24
13.5	1	3	µg	LBK	p-SS316	1.074	0.375	25
13.5	2	3	µg	LBK	p-SS316	0.922	0.351	1
13.5	2	3	µg	LBK	p-SS316	0.946	0.359	2
13.5	2	3	µg	LBK	p-SS316	1.259	0.311	3
13.5	2	3	µg	LBK	p-SS316	0.885	0.245	4
13.5	2	3	µg	LBK	p-SS316	1.045	0.308	5
13.5	2	3	µg	LBK	p-SS316	0.894	0.328	6
13.5	2	3	µg	LBK	p-SS316	0.726	0.257	7
13.5	2	3	µg	LBK	p-SS316	0.881	0.317	8
13.5	2	3	µg	LBK	p-SS316	1.157	0.348	9
13.5	2	3	µg	LBK	p-SS316	1.223	0.301	10
13.5	2	3	µg	LBK	p-SS316	0.835	0.347	11
13.5	2	3	µg	LBK	p-SS316	1.053	0.381	12
13.5	2	3	µg	LBK	p-SS316	1.277	0.373	13
13.5	2	3	µg	LBK	p-SS316	1.203	0.286	14
13.5	2	3	µg	LBK	p-SS316	0.846	0.376	15
13.5	2	3	µg	LBK	p-SS316	0.77	0.262	16
13.5	2	3	µg	LBK	p-SS316	0.713	0.297	17
13.5	2	3	µg	LBK	p-SS316	0.959	0.358	18
13.5	2	3	µg	LBK	p-SS316	0.886	0.292	19
13.5	2	3	µg	LBK	p-SS316	0.756	0.383	20
13.5	2	3	µg	LBK	p-SS316	1.424	0.342	21
13.5	2	3	µg	LBK	p-SS316	0.996	0.274	22

13.5	2	3	µg	LBK	p-SS316	0.928	0.325	23
13.5	2	3	µg	LBK	p-SS316	0.872	0.291	24
13.5	2	3	µg	LBK	p-SS316	0.804	0.32	25
13.5	3	3	µg	LBK	p-SS316	1.175	0.335	1
13.5	3	3	µg	LBK	p-SS316	0.854	0.328	2
13.5	3	3	µg	LBK	p-SS316	0.848	0.348	3
13.5	3	3	µg	LBK	p-SS316	0.921	0.287	4
13.5	3	3	µg	LBK	p-SS316	0.969	0.321	5
13.5	3	3	µg	LBK	p-SS316	1.354	0.27	6
13.5	3	3	µg	LBK	p-SS316	0.914	0.295	7
13.5	3	3	µg	LBK	p-SS316	0.919	0.338	8
13.5	3	3	µg	LBK	p-SS316	1.161	0.261	9
13.5	3	3	µg	LBK	p-SS316	1.174	0.341	10
13.5	3	3	µg	LBK	p-SS316	1.14	0.341	11
13.5	3	3	µg	LBK	p-SS316	0.993	0.282	12
13.5	3	3	µg	LBK	p-SS316	0.983	0.278	13
13.5	3	3	µg	LBK	p-SS316	0.897	0.291	14
13.5	3	3	µg	LBK	p-SS316	1.097	0.277	15
13.5	3	3	µg	LBK	p-SS316	1.028	0.359	16
13.5	3	3	µg	LBK	p-SS316	1.34	0.333	17
13.5	3	3	µg	LBK	p-SS316	1.626	0.293	18
13.5	3	3	µg	LBK	p-SS316	0.77	0.321	19
13.5	3	3	µg	LBK	p-SS316	1.583	0.339	20
13.5	3	3	µg	LBK	p-SS316	1.166	0.357	21
13.5	3	3	µg	LBK	p-SS316	1.354	0.336	22
13.5	3	3	µg	LBK	p-SS316	1.165	0.333	23
13.5	3	3	µg	LBK	p-SS316	1.246	0.33	24
13.5	3	3	µg	LBK	p-SS316	1.34	0.311	25
13.5	4	3	µg	LBK	p-SS316	1.514	0.287	1
13.5	4	3	µg	LBK	p-SS316	1.228	0.336	2
13.5	4	3	µg	LBK	p-SS316	1.198	0.31	3
13.5	4	3	µg	LBK	p-SS316	1.73	0.349	4
13.5	4	3	µg	LBK	p-SS316	1.431	0.293	5
13.5	4	3	µg	LBK	p-SS316	1.083	0.34	6
13.5	4	3	µg	LBK	p-SS316	1.639	0.332	7
13.5	4	3	µg	LBK	p-SS316	0.785	0.261	8
13.5	4	3	µg	LBK	p-SS316	0.828	0.311	9
13.5	4	3	µg	LBK	p-SS316	1.745	0.307	10
13.5	4	3	µg	LBK	p-SS316	1.416	0.308	11
13.5	4	3	µg	LBK	p-SS316	1.205	0.29	12
13.5	4	3	µg	LBK	p-SS316	0.918	0.31	13
13.5	4	3	µg	LBK	p-SS316	1.063	0.339	14
13.5	4	3	µg	LBK	p-SS316	1.148	0.376	15
13.5	4	3	µg	LBK	p-SS316	1.058	0.349	16
13.5	4	3	µg	LBK	p-SS316	0.991	0.277	17
13.5	4	3	µg	LBK	p-SS316	1.366	0.357	18
13.5	4	3	µg	LBK	p-SS316	1.641	0.369	19
13.5	4	3	µg	LBK	p-SS316	1.264	0.397	20
13.5	4	3	µg	LBK	p-SS316	1.053	0.369	21
13.5	4	3	µg	LBK	p-SS316	1.189	0.407	22

13.5	4	3	µg	LBK	p-SS316	0.79	0.358	23
13.5	4	3	µg	LBK	p-SS316	0.747	0.323	24
13.5	4	3	µg	LBK	p-SS316	1.343	0.319	25
14.1	1	3	µg	LBK	MIT-LIS	1.346	0.335	1
14.1	1	3	µg	LBK	MIT-LIS	1.427	0.308	2
14.1	1	3	µg	LBK	MIT-LIS	0.756	0.292	3
14.1	1	3	µg	LBK	MIT-LIS	1.243	0.266	4
14.1	1	3	µg	LBK	MIT-LIS	1.339	0.298	5
14.1	1	3	µg	LBK	MIT-LIS	0.968	0.323	6
14.1	1	3	µg	LBK	MIT-LIS	1.103	0.323	7
14.1	1	3	µg	LBK	MIT-LIS	1.014	0.332	8
14.1	1	3	µg	LBK	MIT-LIS	0.881	0.332	9
14.1	1	3	µg	LBK	MIT-LIS	0.992	0.367	10
14.1	1	3	µg	LBK	MIT-LIS	0.854	0.269	11
14.1	1	3	µg	LBK	MIT-LIS	0.77	0.325	12
14.1	1	3	µg	LBK	MIT-LIS	0.62	0.298	13
14.1	1	3	µg	LBK	MIT-LIS	0.939	0.283	14
14.1	1	3	µg	LBK	MIT-LIS	0.836	0.295	15
14.1	1	3	µg	LBK	MIT-LIS	1.152	0.366	16
14.1	1	3	µg	LBK	MIT-LIS	0.814	0.307	17
14.1	1	3	µg	LBK	MIT-LIS	1.365	0.28	18
14.1	1	3	µg	LBK	MIT-LIS	1.052	0.343	19
14.1	1	3	µg	LBK	MIT-LIS	0.851	0.315	20
14.1	1	3	µg	LBK	MIT-LIS	1.028	0.361	21
14.1	1	3	µg	LBK	MIT-LIS	1.427	0.355	22
14.1	1	3	µg	LBK	MIT-LIS	1.156	0.355	23
14.1	1	3	µg	LBK	MIT-LIS	1.736	0.253	24
14.1	1	3	µg	LBK	MIT-LIS	1.243	0.308	25
14.1	2	3	µg	LBK	MIT-LIS	1.463	0.307	1
14.1	2	3	µg	LBK	MIT-LIS	1.035	0.333	2
14.1	2	3	µg	LBK	MIT-LIS	1.062	0.296	3
14.1	2	3	µg	LBK	MIT-LIS	1.123	0.313	4
14.1	2	3	µg	LBK	MIT-LIS	0.915	0.324	5
14.1	2	3	µg	LBK	MIT-LIS	1.556	0.317	6
14.1	2	3	µg	LBK	MIT-LIS	0.654	0.358	7
14.1	2	3	µg	LBK	MIT-LIS	1.008	0.315	8
14.1	2	3	µg	LBK	MIT-LIS	0.955	0.35	9
14.1	2	3	µg	LBK	MIT-LIS	1.024	0.303	10
14.1	2	3	µg	LBK	MIT-LIS	0.821	0.324	11
14.1	2	3	µg	LBK	MIT-LIS	1.829	0.325	12
14.1	2	3	µg	LBK	MIT-LIS	1.209	0.296	13
14.1	2	3	µg	LBK	MIT-LIS	1.245	0.327	14
14.1	2	3	µg	LBK	MIT-LIS	1.917	0.336	15
14.1	2	3	µg	LBK	MIT-LIS	0.988	0.27	16
14.1	2	3	µg	LBK	MIT-LIS	1.184	0.325	17
14.1	2	3	µg	LBK	MIT-LIS	1.415	0.342	18
14.1	2	3	µg	LBK	MIT-LIS	0.926	0.301	19
14.1	2	3	µg	LBK	MIT-LIS	1.277	0.278	20
14.1	2	3	µg	LBK	MIT-LIS	0.818	0.33	21
14.1	2	3	µg	LBK	MIT-LIS	0.833	0.26	22

14.1	2	3	µg	LBK	MIT-LIS	0.604	0.317	23
14.1	2	3	µg	LBK	MIT-LIS	1.16	0.29	24
14.1	2	3	µg	LBK	MIT-LIS	1.049	0.316	25
14.1	3	3	µg	LBK	MIT-LIS	0.781	0.309	1
14.1	3	3	µg	LBK	MIT-LIS	0.823	0.323	2
14.1	3	3	µg	LBK	MIT-LIS	1.042	0.327	3
14.1	3	3	µg	LBK	MIT-LIS	0.839	0.358	4
14.1	3	3	µg	LBK	MIT-LIS	0.592	0.377	5
14.1	3	3	µg	LBK	MIT-LIS	1.221	0.379	6
14.1	3	3	µg	LBK	MIT-LIS	0.904	0.309	7
14.1	3	3	µg	LBK	MIT-LIS	0.888	0.242	8
14.1	3	3	µg	LBK	MIT-LIS	0.639	0.301	9
14.1	3	3	µg	LBK	MIT-LIS	0.561	0.31	10
14.1	3	3	µg	LBK	MIT-LIS	1.335	0.304	11
14.1	3	3	µg	LBK	MIT-LIS	1.227	0.321	12
14.1	3	3	µg	LBK	MIT-LIS	1.622	0.363	13
14.1	3	3	µg	LBK	MIT-LIS	0.819	0.314	14
14.1	3	3	µg	LBK	MIT-LIS	1.06	0.336	15
14.1	3	3	µg	LBK	MIT-LIS	1.618	0.24	16
14.1	3	3	µg	LBK	MIT-LIS	1.124	0.309	17
14.1	3	3	µg	LBK	MIT-LIS	0.942	0.308	18
14.1	3	3	µg	LBK	MIT-LIS	1.144	0.323	19
14.1	3	3	µg	LBK	MIT-LIS	1.201	0.287	20
14.1	3	3	µg	LBK	MIT-LIS	1.078	0.304	21
14.1	3	3	µg	LBK	MIT-LIS	1.323	0.323	22
14.1	3	3	µg	LBK	MIT-LIS	1.332	0.417	23
14.1	3	3	µg	LBK	MIT-LIS	0.97	0.361	24
14.1	3	3	µg	LBK	MIT-LIS	0.885	0.373	25
14.1	4	3	µg	LBK	MIT-LIS	1.176	0.296	1
14.1	4	3	µg	LBK	MIT-LIS	1.141	0.269	2
14.1	4	3	µg	LBK	MIT-LIS	1.09	0.244	3
14.1	4	3	µg	LBK	MIT-LIS	0.827	0.24	4
14.1	4	3	µg	LBK	MIT-LIS	0.939	0.274	5
14.1	4	3	µg	LBK	MIT-LIS	1.015	0.244	6
14.1	4	3	µg	LBK	MIT-LIS	1.145	0.257	7
14.1	4	3	µg	LBK	MIT-LIS	1.274	0.241	8
14.1	4	3	µg	LBK	MIT-LIS	0.75	0.314	9
14.1	4	3	µg	LBK	MIT-LIS	1.152	0.338	10
14.1	4	3	µg	LBK	MIT-LIS	1.207	0.261	11
14.1	4	3	µg	LBK	MIT-LIS	0.828	0.317	12
14.1	4	3	µg	LBK	MIT-LIS	1.373	0.22	13
14.1	4	3	µg	LBK	MIT-LIS	0.961	0.235	14
14.1	4	3	µg	LBK	MIT-LIS	1.206	0.295	15
14.1	4	3	µg	LBK	MIT-LIS	0.85	0.289	16
14.1	4	3	µg	LBK	MIT-LIS	1.472	0.287	17
14.1	4	3	µg	LBK	MIT-LIS	0.799	0.285	18
14.1	4	3	µg	LBK	MIT-LIS	0.95	0.355	19
14.1	4	3	µg	LBK	MIT-LIS	1.384	0.336	20
14.1	4	3	µg	LBK	MIT-LIS	1.03	0.235	21
14.1	4	3	µg	LBK	MIT-LIS	0.934	0.325	22

14.1	4	3	µg	LBK	MIT-LIS	0.925	0.269	23
14.1	4	3	µg	LBK	MIT-LIS	0.622	0.26	24
14.1	4	3	µg	LBK	MIT-LIS	1.024	0.323	25
G2.5	1	1	1 g	mAUM	Cellulose Membrane	0.877	0.371	1
G2.5	1	1	1 g	mAUM	Cellulose Membrane	0.852	0.308	2
G2.5	1	1	1 g	mAUM	Cellulose Membrane	0.712	0.321	3
G2.5	1	1	1 g	mAUM	Cellulose Membrane	0.723	0.329	4
G2.5	1	1	1 g	mAUM	Cellulose Membrane	0.739	0.375	5
G2.5	1	1	1 g	mAUM	Cellulose Membrane	1.439	0.337	6
G2.5	1	1	1 g	mAUM	Cellulose Membrane	0.743	0.292	7
G2.5	1	1	1 g	mAUM	Cellulose Membrane	1.231	0.348	8
G2.5	1	1	1 g	mAUM	Cellulose Membrane	0.799	0.271	9
G2.5	1	1	1 g	mAUM	Cellulose Membrane	1.002	0.308	10
G2.5	1	1	1 g	mAUM	Cellulose Membrane	0.886	0.287	11
G2.5	1	1	1 g	mAUM	Cellulose Membrane	0.861	0.347	12
G2.5	1	1	1 g	mAUM	Cellulose Membrane	0.978	0.297	13
G2.5	1	1	1 g	mAUM	Cellulose Membrane	1.026	0.287	14
G2.5	1	1	1 g	mAUM	Cellulose Membrane	0.511	0.312	15
G2.5	1	1	1 g	mAUM	Cellulose Membrane	0.547	0.385	16
G2.5	1	1	1 g	mAUM	Cellulose Membrane	0.64	0.279	17
G2.5	1	1	1 g	mAUM	Cellulose Membrane	0.798	0.368	18
G2.5	1	1	1 g	mAUM	Cellulose Membrane	0.901	0.331	19
G2.5	1	1	1 g	mAUM	Cellulose Membrane	1.162	0.289	20
G2.5	1	1	1 g	mAUM	Cellulose Membrane	0.74	0.305	21
G2.5	1	1	1 g	mAUM	Cellulose Membrane	0.586	0.375	22
G2.5	1	1	1 g	mAUM	Cellulose Membrane	0.764	0.329	23

G2.5	1	1	1 g	mAUM	Cellulose Membrane	0.754	0.408	24
G2.5	1	1	1 g	mAUM	Cellulose Membrane	0.704	0.288	25
G2.6	2	1	1 g	mAUM	Cellulose Membrane	0.692	0.363	1
G2.6	2	1	1 g	mAUM	Cellulose Membrane	0.607	0.396	2
G2.6	2	1	1 g	mAUM	Cellulose Membrane	0.766	0.38	3
G2.6	2	1	1 g	mAUM	Cellulose Membrane	0.712	0.387	4
G2.6	2	1	1 g	mAUM	Cellulose Membrane	0.809	0.282	5
G2.6	2	1	1 g	mAUM	Cellulose Membrane	0.91	0.363	6
G2.6	2	1	1 g	mAUM	Cellulose Membrane	0.746	0.385	7
G2.6	2	1	1 g	mAUM	Cellulose Membrane	0.856	0.317	8
G2.6	2	1	1 g	mAUM	Cellulose Membrane	0.964	0.332	9
G2.6	2	1	1 g	mAUM	Cellulose Membrane	0.95	0.377	10
G2.6	2	1	1 g	mAUM	Cellulose Membrane	0.747	0.378	11
G2.6	2	1	1 g	mAUM	Cellulose Membrane	1.107	0.261	12
G2.6	2	1	1 g	mAUM	Cellulose Membrane	0.859	0.316	13
G2.6	2	1	1 g	mAUM	Cellulose Membrane	0.855	0.328	14
G2.6	2	1	1 g	mAUM	Cellulose Membrane	0.751	0.398	15
G2.6	2	1	1 g	mAUM	Cellulose Membrane	1.036	0.322	16
G2.6	2	1	1 g	mAUM	Cellulose Membrane	0.822	0.294	17
G2.6	2	1	1 g	mAUM	Cellulose Membrane	0.766	0.362	18
G2.6	2	1	1 g	mAUM	Cellulose Membrane	0.994	0.343	19
G2.6	2	1	1 g	mAUM	Cellulose Membrane	0.782	0.328	20
G2.6	2	1	1 g	mAUM	Cellulose Membrane	0.707	0.314	21
G2.6	2	1	1 g	mAUM	Cellulose Membrane	1.049	0.381	22
G2.6	2	1	1 g	mAUM	Cellulose Membrane	0.751	0.375	23

G2.6	2	1	1 g	mAUM	Cellulose Membrane	0.846	0.353	24
G2.6	2	1	1 g	mAUM	Cellulose Membrane	1.102	0.336	25
G2.7	3	1	1 g	mAUM	Cellulose Membrane	0.871	0.372	1
G2.7	3	1	1 g	mAUM	Cellulose Membrane	0.78	0.353	2
G2.7	3	1	1 g	mAUM	Cellulose Membrane	0.903	0.318	3
G2.7	3	1	1 g	mAUM	Cellulose Membrane	0.945	0.283	4
G2.7	3	1	1 g	mAUM	Cellulose Membrane	0.935	0.309	5
G2.7	3	1	1 g	mAUM	Cellulose Membrane	0.979	0.396	6
G2.7	3	1	1 g	mAUM	Cellulose Membrane	0.788	0.365	7
G2.7	3	1	1 g	mAUM	Cellulose Membrane	0.89	0.317	8
G2.7	3	1	1 g	mAUM	Cellulose Membrane	0.827	0.361	9
G2.7	3	1	1 g	mAUM	Cellulose Membrane	0.748	0.342	10
G2.7	3	1	1 g	mAUM	Cellulose Membrane	0.77	0.288	11
G2.7	3	1	1 g	mAUM	Cellulose Membrane	0.88	0.321	12
G2.7	3	1	1 g	mAUM	Cellulose Membrane	0.65	0.292	13
G2.7	3	1	1 g	mAUM	Cellulose Membrane	0.963	0.233	14
G2.7	3	1	1 g	mAUM	Cellulose Membrane	0.766	0.362	15
G2.7	3	1	1 g	mAUM	Cellulose Membrane	0.788	0.274	16
G2.7	3	1	1 g	mAUM	Cellulose Membrane	0.934	0.3	17
G2.7	3	1	1 g	mAUM	Cellulose Membrane	0.935	0.353	18
G2.7	3	1	1 g	mAUM	Cellulose Membrane	0.958	0.369	19
G2.7	3	1	1 g	mAUM	Cellulose Membrane	0.922	0.378	20
G2.7	3	1	1 g	mAUM	Cellulose Membrane	0.812	0.314	21
G2.7	3	1	1 g	mAUM	Cellulose Membrane	0.969	0.351	22
G2.7	3	1	1 g	mAUM	Cellulose Membrane	1.008	0.276	23

G2.7	3	1	1 g	mAUM	Cellulose Membrane	0.781	0.292	24
G2.7	3	1	1 g	mAUM	Cellulose Membrane	0.773	0.286	25
G2.8	4	1	1 g	mAUM	Cellulose Membrane	0.8	0.311	1
G2.8	4	1	1 g	mAUM	Cellulose Membrane	0.909	0.376	2
G2.8	4	1	1 g	mAUM	Cellulose Membrane	0.742	0.368	3
G2.8	4	1	1 g	mAUM	Cellulose Membrane	1.12	0.302	4
G2.8	4	1	1 g	mAUM	Cellulose Membrane	0.774	0.342	5
G2.8	4	1	1 g	mAUM	Cellulose Membrane	1.016	0.384	6
G2.8	4	1	1 g	mAUM	Cellulose Membrane	0.922	0.287	7
G2.8	4	1	1 g	mAUM	Cellulose Membrane	0.791	0.308	8
G2.8	4	1	1 g	mAUM	Cellulose Membrane	0.99	0.346	9
G2.8	4	1	1 g	mAUM	Cellulose Membrane	0.833	0.339	10
G2.8	4	1	1 g	mAUM	Cellulose Membrane	1.022	0.302	11
G2.8	4	1	1 g	mAUM	Cellulose Membrane	0.92	0.351	12
G2.8	4	1	1 g	mAUM	Cellulose Membrane	0.941	0.315	13
G2.8	4	1	1 g	mAUM	Cellulose Membrane	0.903	0.325	14
G2.8	4	1	1 g	mAUM	Cellulose Membrane	0.875	0.282	15
G2.8	4	1	1 g	mAUM	Cellulose Membrane	0.946	0.255	16
G2.8	4	1	1 g	mAUM	Cellulose Membrane	0.669	0.372	17
G2.8	4	1	1 g	mAUM	Cellulose Membrane	0.773	0.292	18
G2.8	4	1	1 g	mAUM	Cellulose Membrane	0.998	0.252	19
G2.8	4	1	1 g	mAUM	Cellulose Membrane	0.714	0.289	20
G2.8	4	1	1 g	mAUM	Cellulose Membrane	0.937	0.39	21
G2.8	4	1	1 g	mAUM	Cellulose Membrane	0.855	0.427	22
G2.8	4	1	1 g	mAUM	Cellulose Membrane	0.788	0.355	23

G2.8	4	1	1 g	mAUM	Cellulose Membrane	0.688	0.308	24
G2.8	4	1	1 g	mAUM	Cellulose Membrane	0.869	0.329	25
G3.1	1	1	1 g	mAUM	Silicone	1.189	0.625	1
G3.1	1	1	1 g	mAUM	Silicone	1.05	0.488	2
G3.1	1	1	1 g	mAUM	Silicone	1.065	0.5	3
G3.1	1	1	1 g	mAUM	Silicone	1.24	0.52	4
G3.1	1	1	1 g	mAUM	Silicone	1.434	0.574	5
G3.1	1	1	1 g	mAUM	Silicone	1.363	0.481	6
G3.1	1	1	1 g	mAUM	Silicone	1.181	0.481	7
G3.1	1	1	1 g	mAUM	Silicone	1.222	0.487	8
G3.1	1	1	1 g	mAUM	Silicone	1.104	0.526	9
G3.1	1	1	1 g	mAUM	Silicone	1.125	0.573	10
G3.1	1	1	1 g	mAUM	Silicone	1.414	0.561	11
G3.1	1	1	1 g	mAUM	Silicone	1.37	0.566	12
G3.1	1	1	1 g	mAUM	Silicone	1	0.453	13
G3.1	1	1	1 g	mAUM	Silicone	1.265	0.518	14
G3.1	1	1	1 g	mAUM	Silicone	1.903	0.496	15
G3.1	1	1	1 g	mAUM	Silicone	1.622	0.434	16
G3.1	1	1	1 g	mAUM	Silicone	1.684	0.566	17
G3.1	1	1	1 g	mAUM	Silicone	1.88	0.434	18
G3.1	1	1	1 g	mAUM	Silicone	1.276	0.459	19
G3.1	1	1	1 g	mAUM	Silicone	1.501	0.55	20
G3.1	1	1	1 g	mAUM	Silicone	0.902	0.555	21
G3.1	1	1	1 g	mAUM	Silicone	1.241	0.481	22
G3.1	1	1	1 g	mAUM	Silicone	1.281	0.544	23
G3.1	1	1	1 g	mAUM	Silicone	1.339	0.55	24
G3.1	1	1	1 g	mAUM	Silicone	1.158	0.503	25
G3.2	2	1	1 g	mAUM	Silicone	1.165	0.544	1
G3.2	2	1	1 g	mAUM	Silicone	0.951	0.412	2
G3.2	2	1	1 g	mAUM	Silicone	1.414	0.48	3
G3.2	2	1	1 g	mAUM	Silicone	1.512	0.466	4
G3.2	2	1	1 g	mAUM	Silicone	1.349	0.482	5
G3.2	2	1	1 g	mAUM	Silicone	1.362	0.456	6
G3.2	2	1	1 g	mAUM	Silicone	1.2	0.454	7
G3.2	2	1	1 g	mAUM	Silicone	1.686	0.483	8
G3.2	2	1	1 g	mAUM	Silicone	1.325	0.369	9
G3.2	2	1	1 g	mAUM	Silicone	1.432	0.509	10
G3.2	2	1	1 g	mAUM	Silicone	0.967	0.412	11
G3.2	2	1	1 g	mAUM	Silicone	1.6	0.427	12
G3.2	2	1	1 g	mAUM	Silicone	1.642	0.52	13
G3.2	2	1	1 g	mAUM	Silicone	1.05	0.509	14
G3.2	2	1	1 g	mAUM	Silicone	1.616	0.4	15
G3.2	2	1	1 g	mAUM	Silicone	1.216	0.396	16
G3.2	2	1	1 g	mAUM	Silicone	1.291	0.434	17
G3.2	2	1	1 g	mAUM	Silicone	0.933	0.482	18
G3.2	2	1	1 g	mAUM	Silicone	1.287	0.573	19
G3.2	2	1	1 g	mAUM	Silicone	1.34	0.492	20
G3.2	2	1	1 g	mAUM	Silicone	1.444	0.453	21

G3.2	2	1	1 g	mAUM	Silicone	1.024	0.453	22
G3.2	2	1	1 g	mAUM	Silicone	1.528	0.566	23
G3.2	2	1	1 g	mAUM	Silicone	1.185	0.44	24
G3.2	2	1	1 g	mAUM	Silicone	1.182	0.501	25
G3.3	3	1	1 g	mAUM	Silicone	1.819	0.522	1
G3.3	3	1	1 g	mAUM	Silicone	1.342	0.447	2
G3.3	3	1	1 g	mAUM	Silicone	1.043	0.4	3
G3.3	3	1	1 g	mAUM	Silicone	1.565	0.506	4
G3.3	3	1	1 g	mAUM	Silicone	0.963	0.442	5
G3.3	3	1	1 g	mAUM	Silicone	0.967	0.495	6
G3.3	3	1	1 g	mAUM	Silicone	1.211	0.442	7
G3.3	3	1	1 g	mAUM	Silicone	1.039	0.422	8
G3.3	3	1	1 g	mAUM	Silicone	1.358	0.414	9
G3.3	3	1	1 g	mAUM	Silicone	1.154	0.441	10
G3.3	3	1	1 g	mAUM	Silicone	1.11	0.555	11
G3.3	3	1	1 g	mAUM	Silicone	1.191	0.529	12
G3.3	3	1	1 g	mAUM	Silicone	1.05	0.513	13
G3.3	3	1	1 g	mAUM	Silicone	1.117	0.339	14
G3.3	3	1	1 g	mAUM	Silicone	1.705	0.453	15
G3.3	3	1	1 g	mAUM	Silicone	1.733	0.472	16
G3.3	3	1	1 g	mAUM	Silicone	1.51	0.472	17
G3.3	3	1	1 g	mAUM	Silicone	0.849	0.434	18
G3.3	3	1	1 g	mAUM	Silicone	0.938	0.55	19
G3.3	3	1	1 g	mAUM	Silicone	1.847	0.46	20
G3.3	3	1	1 g	mAUM	Silicone	1.568	0.555	21
G3.3	3	1	1 g	mAUM	Silicone	0.841	0.484	22
G3.3	3	1	1 g	mAUM	Silicone	1.145	0.537	23
G3.3	3	1	1 g	mAUM	Silicone	1.102	0.504	24
G3.3	3	1	1 g	mAUM	Silicone	1.274	0.573	25
G3.4	4	1	1 g	mAUM	Silicone	1.028	0.321	1
G3.4	4	1	1 g	mAUM	Silicone	1.052	0.39	2
G3.4	4	1	1 g	mAUM	Silicone	1.337	0.388	3
G3.4	4	1	1 g	mAUM	Silicone	0.986	0.388	4
G3.4	4	1	1 g	mAUM	Silicone	0.994	0.379	5
G3.4	4	1	1 g	mAUM	Silicone	1.001	0.361	6
G3.4	4	1	1 g	mAUM	Silicone	1.146	0.303	7
G3.4	4	1	1 g	mAUM	Silicone	1.036	0.363	8
G3.4	4	1	1 g	mAUM	Silicone	1.177	0.312	9
G3.4	4	1	1 g	mAUM	Silicone	1.041	0.351	10
G3.4	4	1	1 g	mAUM	Silicone	1.104	0.281	11
G3.4	4	1	1 g	mAUM	Silicone	0.857	0.322	12
G3.4	4	1	1 g	mAUM	Silicone	0.908	0.35	13
G3.4	4	1	1 g	mAUM	Silicone	1.03	0.277	14
G3.4	4	1	1 g	mAUM	Silicone	0.855	0.258	15
G3.4	4	1	1 g	mAUM	Silicone	1.078	0.312	16
G3.4	4	1	1 g	mAUM	Silicone	0.986	0.354	17
G3.4	4	1	1 g	mAUM	Silicone	1.21	0.331	18
G3.4	4	1	1 g	mAUM	Silicone	1.041	0.313	19
G3.4	4	1	1 g	mAUM	Silicone	0.944	0.342	20
G3.4	4	1	1 g	mAUM	Silicone	1.079	0.241	21

G3.4	4	1	1 g	mAUM	Silicone	0.863	0.321	22
G3.4	4	1	1 g	mAUM	Silicone	0.949	0.327	23
G3.4	4	1	1 g	mAUM	Silicone	1.191	0.379	24
G3.4	4	1	1 g	mAUM	Silicone	1.116	0.282	25
G3.5	1	1	1 g	mAUM	Silicone-DLIP	1.338	0.526	1
G3.5	1	1	1 g	mAUM	Silicone-DLIP	0.998	0.453	2
G3.5	1	1	1 g	mAUM	Silicone-DLIP	1.504	0.503	3
G3.5	1	1	1 g	mAUM	Silicone-DLIP	1.195	0.503	4
G3.5	1	1	1 g	mAUM	Silicone-DLIP	1.158	0.549	5
G3.5	1	1	1 g	mAUM	Silicone-DLIP	1.506	0.528	6
G3.5	1	1	1 g	mAUM	Silicone-DLIP	1.337	0.512	7
G3.5	1	1	1 g	mAUM	Silicone-DLIP	1.137	0.564	8
G3.5	1	1	1 g	mAUM	Silicone-DLIP	1.029	0.402	9
G3.5	1	1	1 g	mAUM	Silicone-DLIP	1.408	0.483	10
G3.5	1	1	1 g	mAUM	Silicone-DLIP	0.918	0.54	11
G3.5	1	1	1 g	mAUM	Silicone-DLIP	1.159	0.481	12
G3.5	1	1	1 g	mAUM	Silicone-DLIP	1.245	0.538	13
G3.5	1	1	1 g	mAUM	Silicone-DLIP	1.482	0.488	14
G3.5	1	1	1 g	mAUM	Silicone-DLIP	1.288	0.509	15
G3.5	1	1	1 g	mAUM	Silicone-DLIP	1.243	0.469	16
G3.5	1	1	1 g	mAUM	Silicone-DLIP	1.206	0.48	17
G3.5	1	1	1 g	mAUM	Silicone-DLIP	1.203	0.484	18
G3.5	1	1	1 g	mAUM	Silicone-DLIP	1.221	0.495	19
G3.5	1	1	1 g	mAUM	Silicone-DLIP	1.261	0.469	20
G3.5	1	1	1 g	mAUM	Silicone-DLIP	1.248	0.53	21
G3.5	1	1	1 g	mAUM	Silicone-DLIP	1.308	0.473	22
G3.5	1	1	1 g	mAUM	Silicone-DLIP	0.938	0.55	23
G3.5	1	1	1 g	mAUM	Silicone-DLIP	0.978	0.49	24
G3.5	1	1	1 g	mAUM	Silicone-DLIP	1.158	0.481	25
G3.6	2	1	1 g	mAUM	Silicone-DLIP	1.417	0.456	1
G3.6	2	1	1 g	mAUM	Silicone-DLIP	1.707	0.569	2
G3.6	2	1	1 g	mAUM	Silicone-DLIP	1.597	0.526	3
G3.6	2	1	1 g	mAUM	Silicone-DLIP	0.896	0.608	4
G3.6	2	1	1 g	mAUM	Silicone-DLIP	1.185	0.418	5
G3.6	2	1	1 g	mAUM	Silicone-DLIP	1.298	0.528	6
G3.6	2	1	1 g	mAUM	Silicone-DLIP	1.427	0.584	7
G3.6	2	1	1 g	mAUM	Silicone-DLIP	1.467	0.5	8
G3.6	2	1	1 g	mAUM	Silicone-DLIP	1.5	0.44	9
G3.6	2	1	1 g	mAUM	Silicone-DLIP	1.642	0.563	10
G3.6	2	1	1 g	mAUM	Silicone-DLIP	1.165	0.518	11
G3.6	2	1	1 g	mAUM	Silicone-DLIP	1.36	0.427	12
G3.6	2	1	1 g	mAUM	Silicone-DLIP	1.558	0.487	13
G3.6	2	1	1 g	mAUM	Silicone-DLIP	1.613	0.552	14
G3.6	2	1	1 g	mAUM	Silicone-DLIP	1.768	0.456	15
G3.6	2	1	1 g	mAUM	Silicone-DLIP	1.146	0.497	16
G3.6	2	1	1 g	mAUM	Silicone-DLIP	1.189	0.497	17
G3.6	2	1	1 g	mAUM	Silicone-DLIP	1.642	0.502	18
G3.6	2	1	1 g	mAUM	Silicone-DLIP	1.467	0.507	19
G3.6	2	1	1 g	mAUM	Silicone-DLIP	1.471	0.485	20
G3.6	2	1	1 g	mAUM	Silicone-DLIP	1.161	0.522	21

G3.6	2	1	1 g	mAUM	Silicone-DLIP	1.288	0.497	22
G3.6	2	1	1 g	mAUM	Silicone-DLIP	1.178	0.467	23
G3.6	2	1	1 g	mAUM	Silicone-DLIP	1.131	0.552	24
G3.6	2	1	1 g	mAUM	Silicone-DLIP	1.274	0.548	25
G3.7	3	1	1 g	mAUM	Silicone-DLIP	1.657	0.422	1
G3.7	3	1	1 g	mAUM	Silicone-DLIP	1.335	0.44	2
G3.7	3	1	1 g	mAUM	Silicone-DLIP	1.164	0.4	3
G3.7	3	1	1 g	mAUM	Silicone-DLIP	1.487	0.432	4
G3.7	3	1	1 g	mAUM	Silicone-DLIP	1.427	0.449	5
G3.7	3	1	1 g	mAUM	Silicone-DLIP	1.154	0.484	6
G3.7	3	1	1 g	mAUM	Silicone-DLIP	1.23	0.439	7
G3.7	3	1	1 g	mAUM	Silicone-DLIP	1.344	0.5	8
G3.7	3	1	1 g	mAUM	Silicone-DLIP	1.244	0.512	9
G3.7	3	1	1 g	mAUM	Silicone-DLIP	1.088	0.414	10
G3.7	3	1	1 g	mAUM	Silicone-DLIP	0.96	0.518	11
G3.7	3	1	1 g	mAUM	Silicone-DLIP	1.154	0.456	12
G3.7	3	1	1 g	mAUM	Silicone-DLIP	0.861	0.543	13
G3.7	3	1	1 g	mAUM	Silicone-DLIP	0.849	0.386	14
G3.7	3	1	1 g	mAUM	Silicone-DLIP	0.914	0.402	15
G3.7	3	1	1 g	mAUM	Silicone-DLIP	1.035	0.51	16
G3.7	3	1	1 g	mAUM	Silicone-DLIP	1.497	0.487	17
G3.7	3	1	1 g	mAUM	Silicone-DLIP	1.256	0.447	18
G3.7	3	1	1 g	mAUM	Silicone-DLIP	1.164	0.511	19
G3.7	3	1	1 g	mAUM	Silicone-DLIP	1.331	0.46	20
G3.7	3	1	1 g	mAUM	Silicone-DLIP	1.109	0.441	21
G3.7	3	1	1 g	mAUM	Silicone-DLIP	1.521	0.42	22
G3.7	3	1	1 g	mAUM	Silicone-DLIP	0.913	0.464	23
G3.7	3	1	1 g	mAUM	Silicone-DLIP	1.422	0.46	24
G3.7	3	1	1 g	mAUM	Silicone-DLIP	1.288	0.457	25
G3.8	4	1	1 g	mAUM	Silicone-DLIP	1.418	0.441	1
G3.8	4	1	1 g	mAUM	Silicone-DLIP	1.05	0.573	2
G3.8	4	1	1 g	mAUM	Silicone-DLIP	1.065	0.544	3
G3.8	4	1	1 g	mAUM	Silicone-DLIP	0.948	0.456	4
G3.8	4	1	1 g	mAUM	Silicone-DLIP	1.161	0.44	5
G3.8	4	1	1 g	mAUM	Silicone-DLIP	0.956	0.53	6
G3.8	4	1	1 g	mAUM	Silicone-DLIP	1.456	0.5	7
G3.8	4	1	1 g	mAUM	Silicone-DLIP	1.34	0.381	8
G3.8	4	1	1 g	mAUM	Silicone-DLIP	0.74	0.51	9
G3.8	4	1	1 g	mAUM	Silicone-DLIP	1.16	0.497	10
G3.8	4	1	1 g	mAUM	Silicone-DLIP	1.218	0.608	11
G3.8	4	1	1 g	mAUM	Silicone-DLIP	1.344	0.425	12
G3.8	4	1	1 g	mAUM	Silicone-DLIP	1.082	0.513	13
G3.8	4	1	1 g	mAUM	Silicone-DLIP	0.84	0.497	14
G3.8	4	1	1 g	mAUM	Silicone-DLIP	1.116	0.467	15
G3.8	4	1	1 g	mAUM	Silicone-DLIP	1.081	0.453	16
G3.8	4	1	1 g	mAUM	Silicone-DLIP	0.751	0.477	17
G3.8	4	1	1 g	mAUM	Silicone-DLIP	1.467	0.459	18
G3.8	4	1	1 g	mAUM	Silicone-DLIP	0.882	0.533	19
G3.8	4	1	1 g	mAUM	Silicone-DLIP	0.987	0.498	20
G3.8	4	1	1 g	mAUM	Silicone-DLIP	1.016	0.544	21

G3.8	4	1	1 g	mAUM	Silicone-DLIP	1.095	0.417	22
G3.8	4	1	1 g	mAUM	Silicone-DLIP	1.093	0.512	23
G3.8	4	1	1 g	mAUM	Silicone-DLIP	0.999	0.495	24
G3.8	4	1	1 g	mAUM	Silicone-DLIP	1.175	0.4	25
G8.5	1	2	1 g	mAUM	Cellulose Membrane	1.039	0.359	1
G8.5	1	2	1 g	mAUM	Cellulose Membrane	0.502	0.384	2
G8.5	1	2	1 g	mAUM	Cellulose Membrane	0.789	0.321	3
G8.5	1	2	1 g	mAUM	Cellulose Membrane	0.585	0.321	4
G8.5	1	2	1 g	mAUM	Cellulose Membrane	0.619	0.321	5
G8.5	1	2	1 g	mAUM	Cellulose Membrane	0.942	0.352	6
G8.5	1	2	1 g	mAUM	Cellulose Membrane	1.245	0.378	7
G8.5	1	2	1 g	mAUM	Cellulose Membrane	0.815	0.347	8
G8.5	1	2	1 g	mAUM	Cellulose Membrane	1.03	0.308	9
G8.5	1	2	1 g	mAUM	Cellulose Membrane	0.679	0.302	10
G8.5	1	2	1 g	mAUM	Cellulose Membrane	1.166	0.326	11
G8.5	1	2	1 g	mAUM	Cellulose Membrane	0.986	0.361	12
G8.5	1	2	1 g	mAUM	Cellulose Membrane	0.991	0.322	13
G8.5	1	2	1 g	mAUM	Cellulose Membrane	0.635	0.367	14
G8.5	1	2	1 g	mAUM	Cellulose Membrane	0.719	0.322	15
G8.5	1	2	1 g	mAUM	Cellulose Membrane	0.593	0.329	16
G8.5	1	2	1 g	mAUM	Cellulose Membrane	0.741	0.349	17
G8.5	1	2	1 g	mAUM	Cellulose Membrane	0.853	0.317	18
G8.5	1	2	1 g	mAUM	Cellulose Membrane	0.753	0.326	19
G8.5	1	2	1 g	mAUM	Cellulose Membrane	0.796	0.332	20
G8.5	1	2	1 g	mAUM	Cellulose Membrane	0.762	0.281	21
G8.5	1	2	1 g	mAUM	Cellulose Membrane	0.898	0.332	22
G8.5	1	2	1 g	mAUM	Cellulose Membrane	0.508	0.331	23

G8.5	1	2	1 g	mAUM	Cellulose Membrane	0.676	0.311	24
G8.5	1	2	1 g	mAUM	Cellulose Membrane	0.622	0.391	25
G8.6	2	2	1 g	mAUM	Cellulose Membrane	0.789	0.381	1
G8.6	2	2	1 g	mAUM	Cellulose Membrane	0.752	0.292	2
G8.6	2	2	1 g	mAUM	Cellulose Membrane	0.986	0.346	3
G8.6	2	2	1 g	mAUM	Cellulose Membrane	0.897	0.343	4
G8.6	2	2	1 g	mAUM	Cellulose Membrane	0.927	0.297	5
G8.6	2	2	1 g	mAUM	Cellulose Membrane	0.656	0.341	6
G8.6	2	2	1 g	mAUM	Cellulose Membrane	0.896	0.328	7
G8.6	2	2	1 g	mAUM	Cellulose Membrane	0.754	0.333	8
G8.6	2	2	1 g	mAUM	Cellulose Membrane	0.731	0.282	9
G8.6	2	2	1 g	mAUM	Cellulose Membrane	0.693	0.353	10
G8.6	2	2	1 g	mAUM	Cellulose Membrane	0.67	0.361	11
G8.6	2	2	1 g	mAUM	Cellulose Membrane	0.643	0.242	12
G8.6	2	2	1 g	mAUM	Cellulose Membrane	0.625	0.285	13
G8.6	2	2	1 g	mAUM	Cellulose Membrane	0.791	0.303	14
G8.6	2	2	1 g	mAUM	Cellulose Membrane	0.751	0.316	15
G8.6	2	2	1 g	mAUM	Cellulose Membrane	0.648	0.376	16
G8.6	2	2	1 g	mAUM	Cellulose Membrane	0.705	0.36	17
G8.6	2	2	1 g	mAUM	Cellulose Membrane	0.731	0.446	18
G8.6	2	2	1 g	mAUM	Cellulose Membrane	0.663	0.29	19
G8.6	2	2	1 g	mAUM	Cellulose Membrane	0.895	0.324	20
G8.6	2	2	1 g	mAUM	Cellulose Membrane	0.925	0.324	21
G8.6	2	2	1 g	mAUM	Cellulose Membrane	0.887	0.323	22
G8.6	2	2	1 g	mAUM	Cellulose Membrane	0.801	0.329	23

G8.6	2	2	1 g	mAUM	Cellulose Membrane	0.699	0.314	24
G8.6	2	2	1 g	mAUM	Cellulose Membrane	0.869	0.301	25
G8.7	3	2	1 g	mAUM	Cellulose Membrane	0.708	0.288	1
G8.7	3	2	1 g	mAUM	Cellulose Membrane	0.865	0.315	2
G8.7	3	2	1 g	mAUM	Cellulose Membrane	0.834	0.35	3
G8.7	3	2	1 g	mAUM	Cellulose Membrane	0.866	0.301	4
G8.7	3	2	1 g	mAUM	Cellulose Membrane	1.04	0.302	5
G8.7	3	2	1 g	mAUM	Cellulose Membrane	0.747	0.363	6
G8.7	3	2	1 g	mAUM	Cellulose Membrane	0.819	0.289	7
G8.7	3	2	1 g	mAUM	Cellulose Membrane	0.899	0.301	8
G8.7	3	2	1 g	mAUM	Cellulose Membrane	0.843	0.318	9
G8.7	3	2	1 g	mAUM	Cellulose Membrane	0.966	0.315	10
G8.7	3	2	1 g	mAUM	Cellulose Membrane	0.857	0.313	11
G8.7	3	2	1 g	mAUM	Cellulose Membrane	0.898	0.337	12
G8.7	3	2	1 g	mAUM	Cellulose Membrane	0.78	0.323	13
G8.7	3	2	1 g	mAUM	Cellulose Membrane	0.879	0.318	14
G8.7	3	2	1 g	mAUM	Cellulose Membrane	0.879	0.341	15
G8.7	3	2	1 g	mAUM	Cellulose Membrane	0.949	0.341	16
G8.7	3	2	1 g	mAUM	Cellulose Membrane	0.868	0.31	17
G8.7	3	2	1 g	mAUM	Cellulose Membrane	1.144	0.392	18
G8.7	3	2	1 g	mAUM	Cellulose Membrane	0.92	0.327	19
G8.7	3	2	1 g	mAUM	Cellulose Membrane	0.793	0.363	20
G8.7	3	2	1 g	mAUM	Cellulose Membrane	1.111	0.35	21
G8.7	3	2	1 g	mAUM	Cellulose Membrane	0.975	0.344	22
G8.7	3	2	1 g	mAUM	Cellulose Membrane	0.965	0.36	23

G8.7	3	2	1 g	mAUM	Cellulose Membrane	0.996	0.305	24
G8.7	3	2	1 g	mAUM	Cellulose Membrane	0.71	0.267	25
G8.8	4	2	1 g	mAUM	Cellulose Membrane	0.973	0.376	1
G8.8	4	2	1 g	mAUM	Cellulose Membrane	0.853	0.27	2
G8.8	4	2	1 g	mAUM	Cellulose Membrane	0.818	0.248	3
G8.8	4	2	1 g	mAUM	Cellulose Membrane	0.796	0.267	4
G8.8	4	2	1 g	mAUM	Cellulose Membrane	0.504	0.278	5
G8.8	4	2	1 g	mAUM	Cellulose Membrane	0.874	0.29	6
G8.8	4	2	1 g	mAUM	Cellulose Membrane	1.036	0.255	7
G8.8	4	2	1 g	mAUM	Cellulose Membrane	0.781	0.299	8
G8.8	4	2	1 g	mAUM	Cellulose Membrane	0.583	0.246	9
G8.8	4	2	1 g	mAUM	Cellulose Membrane	0.703	0.273	10
G8.8	4	2	1 g	mAUM	Cellulose Membrane	0.733	0.312	11
G8.8	4	2	1 g	mAUM	Cellulose Membrane	0.835	0.366	12
G8.8	4	2	1 g	mAUM	Cellulose Membrane	0.885	0.351	13
G8.8	4	2	1 g	mAUM	Cellulose Membrane	0.751	0.305	14
G8.8	4	2	1 g	mAUM	Cellulose Membrane	0.872	0.252	15
G8.8	4	2	1 g	mAUM	Cellulose Membrane	0.782	0.327	16
G8.8	4	2	1 g	mAUM	Cellulose Membrane	0.668	0.292	17
G8.8	4	2	1 g	mAUM	Cellulose Membrane	0.697	0.265	18
G8.8	4	2	1 g	mAUM	Cellulose Membrane	0.997	0.303	19
G8.8	4	2	1 g	mAUM	Cellulose Membrane	0.598	0.323	20
G8.8	4	2	1 g	mAUM	Cellulose Membrane	0.566	0.295	21
G8.8	4	2	1 g	mAUM	Cellulose Membrane	0.592	0.239	22
G8.8	4	2	1 g	mAUM	Cellulose Membrane	0.838	0.358	23

G8.8	4	2	1 g	mAUM	Cellulose Membrane	0.761	0.311	24
G8.8	4	2	1 g	mAUM	Cellulose Membrane	0.568	0.342	25
G9.1	1	2	1 g	mAUM	Silicone	0.962	0.306	1
G9.1	1	2	1 g	mAUM	Silicone	1.033	0.313	2
G9.1	1	2	1 g	mAUM	Silicone	0.705	0.399	3
G9.1	1	2	1 g	mAUM	Silicone	0.645	0.336	4
G9.1	1	2	1 g	mAUM	Silicone	0.837	0.362	5
G9.1	1	2	1 g	mAUM	Silicone	0.697	0.353	6
G9.1	1	2	1 g	mAUM	Silicone	0.735	0.284	7
G9.1	1	2	1 g	mAUM	Silicone	0.775	0.315	8
G9.1	1	2	1 g	mAUM	Silicone	1.099	0.355	9
G9.1	1	2	1 g	mAUM	Silicone	0.939	0.313	10
G9.1	1	2	1 g	mAUM	Silicone	0.754	0.35	11
G9.1	1	2	1 g	mAUM	Silicone	0.734	0.334	12
G9.1	1	2	1 g	mAUM	Silicone	0.621	0.314	13
G9.1	1	2	1 g	mAUM	Silicone	0.731	0.301	14
G9.1	1	2	1 g	mAUM	Silicone	0.829	0.228	15
G9.1	1	2	1 g	mAUM	Silicone	0.8	0.269	16
G9.1	1	2	1 g	mAUM	Silicone	0.812	0.404	17
G9.1	1	2	1 g	mAUM	Silicone	0.9	0.329	18
G9.1	1	2	1 g	mAUM	Silicone	0.801	0.305	19
G9.1	1	2	1 g	mAUM	Silicone	0.699	0.377	20
G9.1	1	2	1 g	mAUM	Silicone	1.054	0.39	21
G9.1	1	2	1 g	mAUM	Silicone	0.818	0.288	22
G9.1	1	2	1 g	mAUM	Silicone	0.778	0.285	23
G9.1	1	2	1 g	mAUM	Silicone	0.79	0.382	24
G9.1	1	2	1 g	mAUM	Silicone	0.955	0.295	25
G9.2	2	2	1 g	mAUM	Silicone	0.806	0.301	1
G9.2	2	2	1 g	mAUM	Silicone	0.894	0.366	2
G9.2	2	2	1 g	mAUM	Silicone	0.71	0.412	3
G9.2	2	2	1 g	mAUM	Silicone	0.777	0.288	4
G9.2	2	2	1 g	mAUM	Silicone	0.867	0.303	5
G9.2	2	2	1 g	mAUM	Silicone	0.753	0.35	6
G9.2	2	2	1 g	mAUM	Silicone	1.036	0.282	7
G9.2	2	2	1 g	mAUM	Silicone	0.907	0.298	8
G9.2	2	2	1 g	mAUM	Silicone	1.096	0.372	9
G9.2	2	2	1 g	mAUM	Silicone	0.96	0.319	10
G9.2	2	2	1 g	mAUM	Silicone	1.141	0.365	11
G9.2	2	2	1 g	mAUM	Silicone	0.718	0.296	12
G9.2	2	2	1 g	mAUM	Silicone	0.677	0.298	13
G9.2	2	2	1 g	mAUM	Silicone	0.791	0.409	14
G9.2	2	2	1 g	mAUM	Silicone	1.265	0.419	15
G9.2	2	2	1 g	mAUM	Silicone	0.815	0.294	16
G9.2	2	2	1 g	mAUM	Silicone	1.135	0.323	17
G9.2	2	2	1 g	mAUM	Silicone	1.25	0.258	18
G9.2	2	2	1 g	mAUM	Silicone	1.089	0.244	19
G9.2	2	2	1 g	mAUM	Silicone	0.996	0.319	20
G9.2	2	2	1 g	mAUM	Silicone	1.004	0.317	21

G9.2	2	2	1 g	mAUM	Silicone	1.319	0.249	22
G9.2	2	2	1 g	mAUM	Silicone	0.842	0.329	23
G9.2	2	2	1 g	mAUM	Silicone	0.634	0.308	24
G9.2	2	2	1 g	mAUM	Silicone	0.885	0.384	25
G9.3	3	2	1 g	mAUM	Silicone	0.839	0.296	1
G9.3	3	2	1 g	mAUM	Silicone	0.978	0.294	2
G9.3	3	2	1 g	mAUM	Silicone	0.753	0.303	3
G9.3	3	2	1 g	mAUM	Silicone	0.799	0.314	4
G9.3	3	2	1 g	mAUM	Silicone	1.136	0.284	5
G9.3	3	2	1 g	mAUM	Silicone	0.704	0.314	6
G9.3	3	2	1 g	mAUM	Silicone	0.905	0.302	7
G9.3	3	2	1 g	mAUM	Silicone	1.024	0.304	8
G9.3	3	2	1 g	mAUM	Silicone	0.856	0.276	9
G9.3	3	2	1 g	mAUM	Silicone	0.647	0.315	10
G9.3	3	2	1 g	mAUM	Silicone	0.95	0.298	11
G9.3	3	2	1 g	mAUM	Silicone	0.646	0.312	12
G9.3	3	2	1 g	mAUM	Silicone	0.854	0.336	13
G9.3	3	2	1 g	mAUM	Silicone	0.998	0.296	14
G9.3	3	2	1 g	mAUM	Silicone	0.637	0.298	15
G9.3	3	2	1 g	mAUM	Silicone	0.932	0.295	16
G9.3	3	2	1 g	mAUM	Silicone	0.647	0.315	17
G9.3	3	2	1 g	mAUM	Silicone	0.95	0.298	18
G9.3	3	2	1 g	mAUM	Silicone	0.646	0.312	19
G9.3	3	2	1 g	mAUM	Silicone	0.854	0.336	20
G9.3	3	2	1 g	mAUM	Silicone	0.998	0.296	21
G9.3	3	2	1 g	mAUM	Silicone	0.637	0.298	22
G9.3	3	2	1 g	mAUM	Silicone	0.932	0.295	23
G9.3	3	2	1 g	mAUM	Silicone	0.936	0.263	24
G9.3	3	2	1 g	mAUM	Silicone	0.875	0.281	25
G9.4	4	2	1 g	mAUM	Silicone	0.855	0.283	1
G9.4	4	2	1 g	mAUM	Silicone	0.976	0.304	2
G9.4	4	2	1 g	mAUM	Silicone	1.131	0.358	3
G9.4	4	2	1 g	mAUM	Silicone	0.88	0.287	4
G9.4	4	2	1 g	mAUM	Silicone	0.771	0.348	5
G9.4	4	2	1 g	mAUM	Silicone	0.844	0.266	6
G9.4	4	2	1 g	mAUM	Silicone	0.867	0.253	7
G9.4	4	2	1 g	mAUM	Silicone	0.981	0.234	8
G9.4	4	2	1 g	mAUM	Silicone	1.101	0.321	9
G9.4	4	2	1 g	mAUM	Silicone	0.796	0.255	10
G9.4	4	2	1 g	mAUM	Silicone	0.71	0.241	11
G9.4	4	2	1 g	mAUM	Silicone	0.72	0.233	12
G9.4	4	2	1 g	mAUM	Silicone	0.739	0.307	13
G9.4	4	2	1 g	mAUM	Silicone	0.723	0.298	14
G9.4	4	2	1 g	mAUM	Silicone	0.641	0.272	15
G9.4	4	2	1 g	mAUM	Silicone	0.966	0.319	16
G9.4	4	2	1 g	mAUM	Silicone	0.742	0.339	17
G9.4	4	2	1 g	mAUM	Silicone	0.779	0.304	18
G9.4	4	2	1 g	mAUM	Silicone	0.639	0.317	19
G9.4	4	2	1 g	mAUM	Silicone	1.151	0.272	20
G9.4	4	2	1 g	mAUM	Silicone	0.614	0.343	21

G9.4	4	2	1 g	mAUM	Silicone	0.927	0.309	22
G9.4	4	2	1 g	mAUM	Silicone	0.675	0.34	23
G9.4	4	2	1 g	mAUM	Silicone	0.631	0.358	24
G9.4	4	2	1 g	mAUM	Silicone	1.067	0.303	25
G9.5	1	2	1 g	mAUM	Silicone-DLIP	1.102	0.403	1
G9.5	1	2	1 g	mAUM	Silicone-DLIP	0.765	0.345	2
G9.5	1	2	1 g	mAUM	Silicone-DLIP	0.717	0.301	3
G9.5	1	2	1 g	mAUM	Silicone-DLIP	0.687	0.328	4
G9.5	1	2	1 g	mAUM	Silicone-DLIP	0.97	0.337	5
G9.5	1	2	1 g	mAUM	Silicone-DLIP	0.86	0.285	6
G9.5	1	2	1 g	mAUM	Silicone-DLIP	0.702	0.25	7
G9.5	1	2	1 g	mAUM	Silicone-DLIP	0.6	0.404	8
G9.5	1	2	1 g	mAUM	Silicone-DLIP	0.914	0.358	9
G9.5	1	2	1 g	mAUM	Silicone-DLIP	0.894	0.346	10
G9.5	1	2	1 g	mAUM	Silicone-DLIP	0.617	0.369	11
G9.5	1	2	1 g	mAUM	Silicone-DLIP	0.884	0.366	12
G9.5	1	2	1 g	mAUM	Silicone-DLIP	0.945	0.38	13
G9.5	1	2	1 g	mAUM	Silicone-DLIP	0.793	0.381	14
G9.5	1	2	1 g	mAUM	Silicone-DLIP	0.729	0.317	15
G9.5	1	2	1 g	mAUM	Silicone-DLIP	1.031	0.381	16
G9.5	1	2	1 g	mAUM	Silicone-DLIP	0.885	0.307	17
G9.5	1	2	1 g	mAUM	Silicone-DLIP	0.752	0.296	18
G9.5	1	2	1 g	mAUM	Silicone-DLIP	0.728	0.307	19
G9.5	1	2	1 g	mAUM	Silicone-DLIP	0.634	0.4	20
G9.5	1	2	1 g	mAUM	Silicone-DLIP	0.898	0.367	21
G9.5	1	2	1 g	mAUM	Silicone-DLIP	0.886	0.433	22
G9.5	1	2	1 g	mAUM	Silicone-DLIP	0.77	0.283	23
G9.5	1	2	1 g	mAUM	Silicone-DLIP	0.688	0.286	24
G9.5	1	2	1 g	mAUM	Silicone-DLIP	1.089	0.358	25
G9.6	2	2	1 g	mAUM	Silicone-DLIP	1.034	0.369	1
G9.6	2	2	1 g	mAUM	Silicone-DLIP	1.006	0.38	2
G9.6	2	2	1 g	mAUM	Silicone-DLIP	0.909	0.333	3
G9.6	2	2	1 g	mAUM	Silicone-DLIP	0.74	0.385	4
G9.6	2	2	1 g	mAUM	Silicone-DLIP	0.902	0.309	5
G9.6	2	2	1 g	mAUM	Silicone-DLIP	0.765	0.315	6
G9.6	2	2	1 g	mAUM	Silicone-DLIP	0.9	0.293	7
G9.6	2	2	1 g	mAUM	Silicone-DLIP	1.231	0.361	8
G9.6	2	2	1 g	mAUM	Silicone-DLIP	0.699	0.334	9
G9.6	2	2	1 g	mAUM	Silicone-DLIP	0.94	0.306	10
G9.6	2	2	1 g	mAUM	Silicone-DLIP	0.97	0.343	11
G9.6	2	2	1 g	mAUM	Silicone-DLIP	0.834	0.324	12
G9.6	2	2	1 g	mAUM	Silicone-DLIP	0.978	0.321	13
G9.6	2	2	1 g	mAUM	Silicone-DLIP	0.816	0.289	14
G9.6	2	2	1 g	mAUM	Silicone-DLIP	0.8	0.296	15
G9.6	2	2	1 g	mAUM	Silicone-DLIP	0.833	0.269	16
G9.6	2	2	1 g	mAUM	Silicone-DLIP	0.936	0.306	17
G9.6	2	2	1 g	mAUM	Silicone-DLIP	0.688	0.315	18
G9.6	2	2	1 g	mAUM	Silicone-DLIP	0.895	0.258	19
G9.6	2	2	1 g	mAUM	Silicone-DLIP	0.772	0.361	20
G9.6	2	2	1 g	mAUM	Silicone-DLIP	1.056	0.349	21

G9.6	2	2	1 g	mAUM	Silicone-DLIP	0.922	0.362	22
G9.6	2	2	1 g	mAUM	Silicone-DLIP	1.041	0.349	23
G9.6	2	2	1 g	mAUM	Silicone-DLIP	1.067	0.293	24
G9.6	2	2	1 g	mAUM	Silicone-DLIP	0.766	0.404	25
G9.7	3	2	1 g	mAUM	Silicone-DLIP	0.76	0.337	1
G9.7	3	2	1 g	mAUM	Silicone-DLIP	0.771	0.332	2
G9.7	3	2	1 g	mAUM	Silicone-DLIP	0.833	0.336	3
G9.7	3	2	1 g	mAUM	Silicone-DLIP	0.829	0.325	4
G9.7	3	2	1 g	mAUM	Silicone-DLIP	0.749	0.329	5
G9.7	3	2	1 g	mAUM	Silicone-DLIP	0.826	0.407	6
G9.7	3	2	1 g	mAUM	Silicone-DLIP	0.895	0.325	7
G9.7	3	2	1 g	mAUM	Silicone-DLIP	0.988	0.289	8
G9.7	3	2	1 g	mAUM	Silicone-DLIP	0.867	0.28	9
G9.7	3	2	1 g	mAUM	Silicone-DLIP	0.728	0.298	10
G9.7	3	2	1 g	mAUM	Silicone-DLIP	0.735	0.367	11
G9.7	3	2	1 g	mAUM	Silicone-DLIP	0.903	0.358	12
G9.7	3	2	1 g	mAUM	Silicone-DLIP	0.779	0.33	13
G9.7	3	2	1 g	mAUM	Silicone-DLIP	0.514	0.333	14
G9.7	3	2	1 g	mAUM	Silicone-DLIP	0.828	0.316	15
G9.7	3	2	1 g	mAUM	Silicone-DLIP	0.735	0.332	16
G9.7	3	2	1 g	mAUM	Silicone-DLIP	0.89	0.247	17
G9.7	3	2	1 g	mAUM	Silicone-DLIP	0.713	0.323	18
G9.7	3	2	1 g	mAUM	Silicone-DLIP	0.755	0.303	19
G9.7	3	2	1 g	mAUM	Silicone-DLIP	0.892	0.319	20
G9.7	3	2	1 g	mAUM	Silicone-DLIP	0.772	0.33	21
G9.7	3	2	1 g	mAUM	Silicone-DLIP	0.931	0.36	22
G9.7	3	2	1 g	mAUM	Silicone-DLIP	0.895	0.325	23
G9.7	3	2	1 g	mAUM	Silicone-DLIP	0.856	0.288	24
G9.7	3	2	1 g	mAUM	Silicone-DLIP	0.794	0.38	25
G9.8	4	2	1 g	mAUM	Silicone-DLIP	1.041	0.315	1
G9.8	4	2	1 g	mAUM	Silicone-DLIP	0.681	0.301	2
G9.8	4	2	1 g	mAUM	Silicone-DLIP	0.894	0.325	3
G9.8	4	2	1 g	mAUM	Silicone-DLIP	0.756	0.323	4
G9.8	4	2	1 g	mAUM	Silicone-DLIP	0.746	0.325	5
G9.8	4	2	1 g	mAUM	Silicone-DLIP	0.753	0.319	6
G9.8	4	2	1 g	mAUM	Silicone-DLIP	1.21	0.321	7
G9.8	4	2	1 g	mAUM	Silicone-DLIP	0.633	0.311	8
G9.8	4	2	1 g	mAUM	Silicone-DLIP	0.577	0.332	9
G9.8	4	2	1 g	mAUM	Silicone-DLIP	0.663	0.314	10
G9.8	4	2	1 g	mAUM	Silicone-DLIP	0.897	0.343	11
G9.8	4	2	1 g	mAUM	Silicone-DLIP	1.012	0.312	12
G9.8	4	2	1 g	mAUM	Silicone-DLIP	0.953	0.325	13
G9.8	4	2	1 g	mAUM	Silicone-DLIP	0.925	0.289	14
G9.8	4	2	1 g	mAUM	Silicone-DLIP	0.971	0.296	15
G9.8	4	2	1 g	mAUM	Silicone-DLIP	1.063	0.321	16
G9.8	4	2	1 g	mAUM	Silicone-DLIP	1.042	0.313	17
G9.8	4	2	1 g	mAUM	Silicone-DLIP	1.128	0.295	18
G9.8	4	2	1 g	mAUM	Silicone-DLIP	0.721	0.286	19
G9.8	4	2	1 g	mAUM	Silicone-DLIP	0.621	0.308	20
G9.8	4	2	1 g	mAUM	Silicone-DLIP	0.831	0.264	21

G9.8	4	2	1 g	mAUM	Silicone-DLIP	0.763	0.274	22
G9.8	4	2	1 g	mAUM	Silicone-DLIP	0.931	0.322	23
G9.8	4	2	1 g	mAUM	Silicone-DLIP	0.793	0.308	24
G9.8	4	2	1 g	mAUM	Silicone-DLIP	0.747	0.279	25
G14.5	1	3	1 g	mAUM	Cellulose Membrane	0.767	0.296	1
G14.5	1	3	1 g	mAUM	Cellulose Membrane	0.627	0.284	2
G14.5	1	3	1 g	mAUM	Cellulose Membrane	0.842	0.254	3
G14.5	1	3	1 g	mAUM	Cellulose Membrane	0.686	0.349	4
G14.5	1	3	1 g	mAUM	Cellulose Membrane	0.952	0.349	5
G14.5	1	3	1 g	mAUM	Cellulose Membrane	0.728	0.259	6
G14.5	1	3	1 g	mAUM	Cellulose Membrane	0.72	0.373	7
G14.5	1	3	1 g	mAUM	Cellulose Membrane	0.916	0.296	8
G14.5	1	3	1 g	mAUM	Cellulose Membrane	0.701	0.283	9
G14.5	1	3	1 g	mAUM	Cellulose Membrane	0.637	0.324	10
G14.5	1	3	1 g	mAUM	Cellulose Membrane	0.828	0.283	11
G14.5	1	3	1 g	mAUM	Cellulose Membrane	0.992	0.288	12
G14.5	1	3	1 g	mAUM	Cellulose Membrane	0.885	0.313	13
G14.5	1	3	1 g	mAUM	Cellulose Membrane	0.771	0.313	14
G14.5	1	3	1 g	mAUM	Cellulose Membrane	0.719	0.358	15
G14.5	1	3	1 g	mAUM	Cellulose Membrane	0.742	0.265	16
G14.5	1	3	1 g	mAUM	Cellulose Membrane	0.678	0.248	17
G14.5	1	3	1 g	mAUM	Cellulose Membrane	0.664	0.321	18
G14.5	1	3	1 g	mAUM	Cellulose Membrane	0.888	0.324	19
G14.5	1	3	1 g	mAUM	Cellulose Membrane	0.745	0.383	20
G14.5	1	3	1 g	mAUM	Cellulose Membrane	0.904	0.321	21
G14.5	1	3	1 g	mAUM	Cellulose Membrane	0.703	0.318	22
G14.5	1	3	1 g	mAUM	Cellulose Membrane	0.728	0.286	23

G14.5	1	3	1 g	mAUM	Cellulose Membrane	0.699	0.28	24
G14.5	1	3	1 g	mAUM	Cellulose Membrane	0.839	0.281	25
G14.6	2	3	1 g	mAUM	Cellulose Membrane	0.769	0.29	1
G14.6	2	3	1 g	mAUM	Cellulose Membrane	0.744	0.295	2
G14.6	2	3	1 g	mAUM	Cellulose Membrane	0.732	0.309	3
G14.6	2	3	1 g	mAUM	Cellulose Membrane	0.667	0.284	4
G14.6	2	3	1 g	mAUM	Cellulose Membrane	0.748	0.272	5
G14.6	2	3	1 g	mAUM	Cellulose Membrane	0.85	0.281	6
G14.6	2	3	1 g	mAUM	Cellulose Membrane	0.65	0.298	7
G14.6	2	3	1 g	mAUM	Cellulose Membrane	0.649	0.289	8
G14.6	2	3	1 g	mAUM	Cellulose Membrane	0.855	0.296	9
G14.6	2	3	1 g	mAUM	Cellulose Membrane	0.689	0.269	10
G14.6	2	3	1 g	mAUM	Cellulose Membrane	0.775	0.277	11
G14.6	2	3	1 g	mAUM	Cellulose Membrane	0.952	0.319	12
G14.6	2	3	1 g	mAUM	Cellulose Membrane	0.761	0.31	13
G14.6	2	3	1 g	mAUM	Cellulose Membrane	0.781	0.269	14
G14.6	2	3	1 g	mAUM	Cellulose Membrane	0.793	0.323	15
G14.6	2	3	1 g	mAUM	Cellulose Membrane	0.879	0.285	16
G14.6	2	3	1 g	mAUM	Cellulose Membrane	0.672	0.27	17
G14.6	2	3	1 g	mAUM	Cellulose Membrane	0.605	0.291	18
G14.6	2	3	1 g	mAUM	Cellulose Membrane	0.978	0.29	19
G14.6	2	3	1 g	mAUM	Cellulose Membrane	0.879	0.274	20
G14.6	2	3	1 g	mAUM	Cellulose Membrane	0.728	0.325	21
G14.6	2	3	1 g	mAUM	Cellulose Membrane	0.923	0.331	22
G14.6	2	3	1 g	mAUM	Cellulose Membrane	0.829	0.36	23

G14.6	2	3	1 g	mAUM	Cellulose Membrane	0.805	0.263	24
G14.6	2	3	1 g	mAUM	Cellulose Membrane	0.804	0.312	25
G14.7	3	3	1 g	mAUM	Cellulose Membrane	0.791	0.319	1
G14.7	3	3	1 g	mAUM	Cellulose Membrane	0.682	0.267	2
G14.7	3	3	1 g	mAUM	Cellulose Membrane	0.88	0.379	3
G14.7	3	3	1 g	mAUM	Cellulose Membrane	0.512	0.319	4
G14.7	3	3	1 g	mAUM	Cellulose Membrane	0.657	0.253	5
G14.7	3	3	1 g	mAUM	Cellulose Membrane	0.788	0.321	6
G14.7	3	3	1 g	mAUM	Cellulose Membrane	0.613	0.269	7
G14.7	3	3	1 g	mAUM	Cellulose Membrane	0.902	0.275	8
G14.7	3	3	1 g	mAUM	Cellulose Membrane	0.617	0.311	9
G14.7	3	3	1 g	mAUM	Cellulose Membrane	0.667	0.319	10
G14.7	3	3	1 g	mAUM	Cellulose Membrane	0.64	0.257	11
G14.7	3	3	1 g	mAUM	Cellulose Membrane	0.681	0.296	12
G14.7	3	3	1 g	mAUM	Cellulose Membrane	0.961	0.298	13
G14.7	3	3	1 g	mAUM	Cellulose Membrane	0.711	0.299	14
G14.7	3	3	1 g	mAUM	Cellulose Membrane	0.905	0.254	15
G14.7	3	3	1 g	mAUM	Cellulose Membrane	0.893	0.267	16
G14.7	3	3	1 g	mAUM	Cellulose Membrane	0.873	0.273	17
G14.7	3	3	1 g	mAUM	Cellulose Membrane	0.851	0.297	18
G14.7	3	3	1 g	mAUM	Cellulose Membrane	0.952	0.301	19
G14.7	3	3	1 g	mAUM	Cellulose Membrane	0.819	0.287	20
G14.7	3	3	1 g	mAUM	Cellulose Membrane	0.894	0.313	21
G14.7	3	3	1 g	mAUM	Cellulose Membrane	0.755	0.316	22
G14.7	3	3	1 g	mAUM	Cellulose Membrane	0.744	0.278	23

G14.7	3	3	1 g	mAUM	Cellulose Membrane	0.937	0.334	24
G14.7	3	3	1 g	mAUM	Cellulose Membrane	0.864	0.278	25
G14.8	4	3	1 g	mAUM	Cellulose Membrane	0.757	0.329	1
G14.8	4	3	1 g	mAUM	Cellulose Membrane	0.84	0.333	2
G14.8	4	3	1 g	mAUM	Cellulose Membrane	0.608	0.284	3
G14.8	4	3	1 g	mAUM	Cellulose Membrane	0.766	0.243	4
G14.8	4	3	1 g	mAUM	Cellulose Membrane	0.82	0.282	5
G14.8	4	3	1 g	mAUM	Cellulose Membrane	0.675	0.315	6
G14.8	4	3	1 g	mAUM	Cellulose Membrane	0.766	0.362	7
G14.8	4	3	1 g	mAUM	Cellulose Membrane	0.867	0.369	8
G14.8	4	3	1 g	mAUM	Cellulose Membrane	0.802	0.265	9
G14.8	4	3	1 g	mAUM	Cellulose Membrane	0.528	0.295	10
G14.8	4	3	1 g	mAUM	Cellulose Membrane	0.666	0.266	11
G14.8	4	3	1 g	mAUM	Cellulose Membrane	0.632	0.312	12
G14.8	4	3	1 g	mAUM	Cellulose Membrane	0.906	0.298	13
G14.8	4	3	1 g	mAUM	Cellulose Membrane	0.847	0.312	14
G14.8	4	3	1 g	mAUM	Cellulose Membrane	0.646	0.301	15
G14.8	4	3	1 g	mAUM	Cellulose Membrane	0.564	0.368	16
G14.8	4	3	1 g	mAUM	Cellulose Membrane	0.76	0.301	17
G14.8	4	3	1 g	mAUM	Cellulose Membrane	0.894	0.273	18
G14.8	4	3	1 g	mAUM	Cellulose Membrane	0.774	0.294	19
G14.8	4	3	1 g	mAUM	Cellulose Membrane	0.904	0.29	20
G14.8	4	3	1 g	mAUM	Cellulose Membrane	0.89	0.265	21
G14.8	4	3	1 g	mAUM	Cellulose Membrane	0.914	0.33	22
G14.8	4	3	1 g	mAUM	Cellulose Membrane	1.203	0.252	23

G14.8	4	3	1 g	mAUM	Cellulose Membrane	0.777	0.248	24
G14.8	4	3	1 g	mAUM	Cellulose Membrane	0.653	0.294	25
G15.1	1	3	1 g	mAUM	Silicone	1.13	0.399	1
G15.1	1	3	1 g	mAUM	Silicone	0.839	0.312	2
G15.1	1	3	1 g	mAUM	Silicone	0.803	0.268	3
G15.1	1	3	1 g	mAUM	Silicone	0.856	0.324	4
G15.1	1	3	1 g	mAUM	Silicone	0.685	0.315	5
G15.1	1	3	1 g	mAUM	Silicone	0.893	0.294	6
G15.1	1	3	1 g	mAUM	Silicone	0.791	0.284	7
G15.1	1	3	1 g	mAUM	Silicone	0.784	0.347	8
G15.1	1	3	1 g	mAUM	Silicone	1.047	0.335	9
G15.1	1	3	1 g	mAUM	Silicone	0.955	0.347	10
G15.1	1	3	1 g	mAUM	Silicone	1.13	0.348	11
G15.1	1	3	1 g	mAUM	Silicone	1.1	0.342	12
G15.1	1	3	1 g	mAUM	Silicone	1.166	0.321	13
G15.1	1	3	1 g	mAUM	Silicone	1.004	0.347	14
G15.1	1	3	1 g	mAUM	Silicone	1.232	0.339	15
G15.1	1	3	1 g	mAUM	Silicone	0.727	0.339	16
G15.1	1	3	1 g	mAUM	Silicone	0.882	0.336	17
G15.1	1	3	1 g	mAUM	Silicone	0.758	0.362	18
G15.1	1	3	1 g	mAUM	Silicone	0.997	0.249	19
G15.1	1	3	1 g	mAUM	Silicone	0.821	0.286	20
G15.1	1	3	1 g	mAUM	Silicone	0.728	0.32	21
G15.1	1	3	1 g	mAUM	Silicone	0.75	0.36	22
G15.1	1	3	1 g	mAUM	Silicone	0.871	0.313	23
G15.1	1	3	1 g	mAUM	Silicone	0.731	0.332	24
G15.1	1	3	1 g	mAUM	Silicone	0.91	0.319	25
G15.2	2	3	1 g	mAUM	Silicone	0.751	0.335	1
G15.2	2	3	1 g	mAUM	Silicone	0.71	0.297	2
G15.2	2	3	1 g	mAUM	Silicone	0.707	0.325	3
G15.2	2	3	1 g	mAUM	Silicone	0.884	0.348	4
G15.2	2	3	1 g	mAUM	Silicone	0.616	0.307	5
G15.2	2	3	1 g	mAUM	Silicone	0.922	0.322	6
G15.2	2	3	1 g	mAUM	Silicone	0.723	0.368	7
G15.2	2	3	1 g	mAUM	Silicone	0.839	0.316	8
G15.2	2	3	1 g	mAUM	Silicone	1.055	0.335	9
G15.2	2	3	1 g	mAUM	Silicone	0.955	0.338	10
G15.2	2	3	1 g	mAUM	Silicone	0.973	0.343	11
G15.2	2	3	1 g	mAUM	Silicone	0.773	0.318	12
G15.2	2	3	1 g	mAUM	Silicone	1.157	0.378	13
G15.2	2	3	1 g	mAUM	Silicone	0.881	0.335	14
G15.2	2	3	1 g	mAUM	Silicone	0.811	0.336	15
G15.2	2	3	1 g	mAUM	Silicone	0.806	0.348	16
G15.2	2	3	1 g	mAUM	Silicone	0.79	0.415	17
G15.2	2	3	1 g	mAUM	Silicone	0.76	0.33	18
G15.2	2	3	1 g	mAUM	Silicone	0.637	0.284	19
G15.2	2	3	1 g	mAUM	Silicone	0.83	0.321	20
G15.2	2	3	1 g	mAUM	Silicone	0.659	0.354	21

G15.2	2	3	1 g	mAUM	Silicone	0.967	0.322	22
G15.2	2	3	1 g	mAUM	Silicone	1.011	0.373	23
G15.2	2	3	1 g	mAUM	Silicone	0.961	0.328	24
G15.2	2	3	1 g	mAUM	Silicone	1.017	0.335	25
G15.3	3	3	1 g	mAUM	Silicone	0.722	0.308	1
G15.3	3	3	1 g	mAUM	Silicone	0.682	0.332	2
G15.3	3	3	1 g	mAUM	Silicone	0.69	0.35	3
G15.3	3	3	1 g	mAUM	Silicone	0.889	0.329	4
G15.3	3	3	1 g	mAUM	Silicone	0.949	0.384	5
G15.3	3	3	1 g	mAUM	Silicone	0.845	0.309	6
G15.3	3	3	1 g	mAUM	Silicone	0.749	0.328	7
G15.3	3	3	1 g	mAUM	Silicone	0.8	0.354	8
G15.3	3	3	1 g	mAUM	Silicone	0.752	0.302	9
G15.3	3	3	1 g	mAUM	Silicone	1.054	0.379	10
G15.3	3	3	1 g	mAUM	Silicone	1.064	0.317	11
G15.3	3	3	1 g	mAUM	Silicone	0.899	0.342	12
G15.3	3	3	1 g	mAUM	Silicone	0.685	0.332	13
G15.3	3	3	1 g	mAUM	Silicone	0.99	0.34	14
G15.3	3	3	1 g	mAUM	Silicone	0.85	0.312	15
G15.3	3	3	1 g	mAUM	Silicone	0.873	0.353	16
G15.3	3	3	1 g	mAUM	Silicone	0.707	0.311	17
G15.3	3	3	1 g	mAUM	Silicone	0.926	0.337	18
G15.3	3	3	1 g	mAUM	Silicone	0.564	0.305	19
G15.3	3	3	1 g	mAUM	Silicone	0.841	0.35	20
G15.3	3	3	1 g	mAUM	Silicone	0.839	0.375	21
G15.3	3	3	1 g	mAUM	Silicone	0.804	0.283	22
G15.3	3	3	1 g	mAUM	Silicone	0.66	0.4	23
G15.3	3	3	1 g	mAUM	Silicone	0.863	0.405	24
G15.3	3	3	1 g	mAUM	Silicone	0.921	0.305	25
G15.4	4	3	1 g	mAUM	Silicone	0.821	0.301	1
G15.4	4	3	1 g	mAUM	Silicone	0.718	0.305	2
G15.4	4	3	1 g	mAUM	Silicone	0.682	0.365	3
G15.4	4	3	1 g	mAUM	Silicone	0.688	0.38	4
G15.4	4	3	1 g	mAUM	Silicone	0.735	0.313	5
G15.4	4	3	1 g	mAUM	Silicone	0.738	0.269	6
G15.4	4	3	1 g	mAUM	Silicone	0.864	0.315	7
G15.4	4	3	1 g	mAUM	Silicone	0.746	0.335	8
G15.4	4	3	1 g	mAUM	Silicone	0.724	0.319	9
G15.4	4	3	1 g	mAUM	Silicone	0.726	0.314	10
G15.4	4	3	1 g	mAUM	Silicone	0.699	0.331	11
G15.4	4	3	1 g	mAUM	Silicone	0.753	0.277	12
G15.4	4	3	1 g	mAUM	Silicone	0.819	0.341	13
G15.4	4	3	1 g	mAUM	Silicone	0.632	0.332	14
G15.4	4	3	1 g	mAUM	Silicone	0.706	0.377	15
G15.4	4	3	1 g	mAUM	Silicone	0.692	0.339	16
G15.4	4	3	1 g	mAUM	Silicone	0.814	0.369	17
G15.4	4	3	1 g	mAUM	Silicone	0.888	0.356	18
G15.4	4	3	1 g	mAUM	Silicone	0.897	0.318	19
G15.4	4	3	1 g	mAUM	Silicone	0.675	0.303	20
G15.4	4	3	1 g	mAUM	Silicone	0.741	0.348	21

G15.4	4	3	1 g	mAUM	Silicone	0.611	0.328	22
G15.4	4	3	1 g	mAUM	Silicone	0.748	0.336	23
G15.4	4	3	1 g	mAUM	Silicone	0.704	0.368	24
G15.4	4	3	1 g	mAUM	Silicone	0.747	0.367	25
G15.5	1	3	1 g	mAUM	Silicone-DLIP	1.146	0.439	1
G15.5	1	3	1 g	mAUM	Silicone-DLIP	0.833	0.519	2
G15.5	1	3	1 g	mAUM	Silicone-DLIP	0.981	0.531	3
G15.5	1	3	1 g	mAUM	Silicone-DLIP	0.861	0.48	4
G15.5	1	3	1 g	mAUM	Silicone-DLIP	1.531	0.539	5
G15.5	1	3	1 g	mAUM	Silicone-DLIP	1.179	0.473	6
G15.5	1	3	1 g	mAUM	Silicone-DLIP	1.62	0.495	7
G15.5	1	3	1 g	mAUM	Silicone-DLIP	1.341	0.472	8
G15.5	1	3	1 g	mAUM	Silicone-DLIP	1.122	0.5	9
G15.5	1	3	1 g	mAUM	Silicone-DLIP	1.181	0.513	10
G15.5	1	3	1 g	mAUM	Silicone-DLIP	0.785	0.507	11
G15.5	1	3	1 g	mAUM	Silicone-DLIP	1.368	0.519	12
G15.5	1	3	1 g	mAUM	Silicone-DLIP	1.242	0.483	13
G15.5	1	3	1 g	mAUM	Silicone-DLIP	0.939	0.482	14
G15.5	1	3	1 g	mAUM	Silicone-DLIP	1.252	0.545	15
G15.5	1	3	1 g	mAUM	Silicone-DLIP	1.283	0.56	16
G15.5	1	3	1 g	mAUM	Silicone-DLIP	1.091	0.478	17
G15.5	1	3	1 g	mAUM	Silicone-DLIP	1.36	0.442	18
G15.5	1	3	1 g	mAUM	Silicone-DLIP	1.174	0.467	19
G15.5	1	3	1 g	mAUM	Silicone-DLIP	0.879	0.577	20
G15.5	1	3	1 g	mAUM	Silicone-DLIP	1.352	0.472	21
G15.5	1	3	1 g	mAUM	Silicone-DLIP	1.513	0.528	22
G15.5	1	3	1 g	mAUM	Silicone-DLIP	1.261	0.427	23
G15.5	1	3	1 g	mAUM	Silicone-DLIP	1.505	0.429	24
G15.5	1	3	1 g	mAUM	Silicone-DLIP	1.34	0.417	25
G15.6	2	3	1 g	mAUM	Silicone-DLIP	0.929	0.55	1
G15.6	2	3	1 g	mAUM	Silicone-DLIP	1.024	0.585	2
G15.6	2	3	1 g	mAUM	Silicone-DLIP	1.019	0.453	3
G15.6	2	3	1 g	mAUM	Silicone-DLIP	1.122	0.49	4
G15.6	2	3	1 g	mAUM	Silicone-DLIP	1.03	0.358	5
G15.6	2	3	1 g	mAUM	Silicone-DLIP	1.432	0.441	6
G15.6	2	3	1 g	mAUM	Silicone-DLIP	1.586	0.406	7
G15.6	2	3	1 g	mAUM	Silicone-DLIP	1.339	0.359	8
G15.6	2	3	1 g	mAUM	Silicone-DLIP	1.184	0.506	9
G15.6	2	3	1 g	mAUM	Silicone-DLIP	1.23	0.481	10
G15.6	2	3	1 g	mAUM	Silicone-DLIP	1.411	0.501	11
G15.6	2	3	1 g	mAUM	Silicone-DLIP	1.198	0.473	12
G15.6	2	3	1 g	mAUM	Silicone-DLIP	0.828	0.507	13
G15.6	2	3	1 g	mAUM	Silicone-DLIP	1.012	0.473	14
G15.6	2	3	1 g	mAUM	Silicone-DLIP	1.139	0.408	15
G15.6	2	3	1 g	mAUM	Silicone-DLIP	1.139	0.404	16
G15.6	2	3	1 g	mAUM	Silicone-DLIP	1.19	0.441	17
G15.6	2	3	1 g	mAUM	Silicone-DLIP	1.242	0.447	18
G15.6	2	3	1 g	mAUM	Silicone-DLIP	1.334	0.44	19
G15.6	2	3	1 g	mAUM	Silicone-DLIP	0.8	0.483	20
G15.6	2	3	1 g	mAUM	Silicone-DLIP	1.067	0.546	21

G15.6	2	3	1 g	mAUM	Silicone-DLIP	1.143	0.552	22
G15.6	2	3	1 g	mAUM	Silicone-DLIP	0.905	0.5	23
G15.6	2	3	1 g	mAUM	Silicone-DLIP	1.059	0.467	24
G15.6	2	3	1 g	mAUM	Silicone-DLIP	0.921	0.54	25
G15.7	3	3	1 g	mAUM	Silicone-DLIP	1.075	0.512	1
G15.7	3	3	1 g	mAUM	Silicone-DLIP	0.863	0.488	2
G15.7	3	3	1 g	mAUM	Silicone-DLIP	1.404	0.495	3
G15.7	3	3	1 g	mAUM	Silicone-DLIP	1.315	0.564	4
G15.7	3	3	1 g	mAUM	Silicone-DLIP	1.075	0.449	5
G15.7	3	3	1 g	mAUM	Silicone-DLIP	1.156	0.51	6
G15.7	3	3	1 g	mAUM	Silicone-DLIP	0.985	0.5	7
G15.7	3	3	1 g	mAUM	Silicone-DLIP	1.047	0.433	8
G15.7	3	3	1 g	mAUM	Silicone-DLIP	1.085	0.483	9
G15.7	3	3	1 g	mAUM	Silicone-DLIP	1.07	0.474	10
G15.7	3	3	1 g	mAUM	Silicone-DLIP	0.995	0.428	11
G15.7	3	3	1 g	mAUM	Silicone-DLIP	1.051	0.508	12
G15.7	3	3	1 g	mAUM	Silicone-DLIP	0.874	0.456	13
G15.7	3	3	1 g	mAUM	Silicone-DLIP	1.04	0.573	14
G15.7	3	3	1 g	mAUM	Silicone-DLIP	1.01	0.532	15
G15.7	3	3	1 g	mAUM	Silicone-DLIP	0.948	0.588	16
G15.7	3	3	1 g	mAUM	Silicone-DLIP	1.095	0.523	17
G15.7	3	3	1 g	mAUM	Silicone-DLIP	1.106	0.443	18
G15.7	3	3	1 g	mAUM	Silicone-DLIP	1.149	0.457	19
G15.7	3	3	1 g	mAUM	Silicone-DLIP	1.08	0.585	20
G15.7	3	3	1 g	mAUM	Silicone-DLIP	1.112	0.49	21
G15.7	3	3	1 g	mAUM	Silicone-DLIP	1.221	0.467	22
G15.7	3	3	1 g	mAUM	Silicone-DLIP	1.201	0.54	23
G15.7	3	3	1 g	mAUM	Silicone-DLIP	0.982	0.5	24
G15.7	3	3	1 g	mAUM	Silicone-DLIP	1	0.5	25
G15.8	4	3	1 g	mAUM	Silicone-DLIP	1.36	0.4	1
G15.8	4	3	1 g	mAUM	Silicone-DLIP	1.06	0.433	2
G15.8	4	3	1 g	mAUM	Silicone-DLIP	0.959	0.492	3
G15.8	4	3	1 g	mAUM	Silicone-DLIP	0.933	0.416	4
G15.8	4	3	1 g	mAUM	Silicone-DLIP	0.856	0.577	5
G15.8	4	3	1 g	mAUM	Silicone-DLIP	0.868	0.453	6
G15.8	4	3	1 g	mAUM	Silicone-DLIP	0.891	0.492	7
G15.8	4	3	1 g	mAUM	Silicone-DLIP	0.948	0.534	8
G15.8	4	3	1 g	mAUM	Silicone-DLIP	1.032	0.534	9
G15.8	4	3	1 g	mAUM	Silicone-DLIP	1.107	0.48	10
G15.8	4	3	1 g	mAUM	Silicone-DLIP	0.973	0.508	11
G15.8	4	3	1 g	mAUM	Silicone-DLIP	1.248	0.537	12
G15.8	4	3	1 g	mAUM	Silicone-DLIP	1.294	0.489	13
G15.8	4	3	1 g	mAUM	Silicone-DLIP	1.144	0.496	14
G15.8	4	3	1 g	mAUM	Silicone-DLIP	1.065	0.521	15
G15.8	4	3	1 g	mAUM	Silicone-DLIP	1.086	0.538	16
G15.8	4	3	1 g	mAUM	Silicone-DLIP	1.266	0.488	17
G15.8	4	3	1 g	mAUM	Silicone-DLIP	0.984	0.379	18
G15.8	4	3	1 g	mAUM	Silicone-DLIP	1.171	0.406	19
G15.8	4	3	1 g	mAUM	Silicone-DLIP	1.021	0.553	20
G15.8	4	3	1 g	mAUM	Silicone-DLIP	1.376	0.514	21

G15.8	4	3	1 g	mAUM	Silicone-DLIP	1.348	0.564	22
G15.8	4	3	1 g	mAUM	Silicone-DLIP	1.021	0.528	23
G15.8	4	3	1 g	mAUM	Silicone-DLIP	1.097	0.374	24
G15.8	4	3	1 g	mAUM	Silicone-DLIP	0.928	0.392	25
2.5	1	1	µg	mAUM	Cellulose Membrane	1.187	0.467	1
2.5	1	1	µg	mAUM	Cellulose Membrane	1.159	0.481	2
2.5	1	1	µg	mAUM	Cellulose Membrane	1.101	0.457	3
2.5	1	1	µg	mAUM	Cellulose Membrane	1.149	0.416	4
2.5	1	1	µg	mAUM	Cellulose Membrane	1.101	0.531	5
2.5	1	1	µg	mAUM	Cellulose Membrane	1.244	0.485	6
2.5	1	1	µg	mAUM	Cellulose Membrane	1.143	0.475	7
2.5	1	1	µg	mAUM	Cellulose Membrane	0.979	0.609	8
2.5	1	1	µg	mAUM	Cellulose Membrane	1.195	0.512	9
2.5	1	1	µg	mAUM	Cellulose Membrane	1.205	0.402	10
2.5	1	1	µg	mAUM	Cellulose Membrane	0.985	0.425	11
2.5	1	1	µg	mAUM	Cellulose Membrane	1.187	0.542	12
2.5	1	1	µg	mAUM	Cellulose Membrane	1.03	0.551	13
2.5	1	1	µg	mAUM	Cellulose Membrane	0.976	0.506	14
2.5	1	1	µg	mAUM	Cellulose Membrane	1.14	0.488	15
2.5	1	1	µg	mAUM	Cellulose Membrane	1.201	0.432	16
2.5	1	1	µg	mAUM	Cellulose Membrane	1.134	0.502	17
2.5	1	1	µg	mAUM	Cellulose Membrane	1.024	0.469	18
2.5	1	1	µg	mAUM	Cellulose Membrane	1.161	0.287	19
2.5	1	1	µg	mAUM	Cellulose Membrane	0.938	0.342	20
2.5	1	1	µg	mAUM	Cellulose Membrane	1.262	0.327	21
2.5	1	1	µg	mAUM	Cellulose Membrane	0.781	0.298	22
2.5	1	1	µg	mAUM	Cellulose Membrane	0.855	0.319	23

2.5	1	1	µg	mAUM	Cellulose Membrane	1.096	0.334	24
2.5	1	1	µg	mAUM	Cellulose Membrane	0.976	0.315	25
2.6	2	1	µg	mAUM	Cellulose Membrane	0.831	0.218	1
2.6	2	1	µg	mAUM	Cellulose Membrane	0.861	0.319	2
2.6	2	1	µg	mAUM	Cellulose Membrane	0.852	0.25	3
2.6	2	1	µg	mAUM	Cellulose Membrane	0.611	0.29	4
2.6	2	1	µg	mAUM	Cellulose Membrane	0.873	0.225	5
2.6	2	1	µg	mAUM	Cellulose Membrane	0.749	0.299	6
2.6	2	1	µg	mAUM	Cellulose Membrane	1.123	0.31	7
2.6	2	1	µg	mAUM	Cellulose Membrane	1.074	0.265	8
2.6	2	1	µg	mAUM	Cellulose Membrane	0.635	0.265	9
2.6	2	1	µg	mAUM	Cellulose Membrane	0.668	0.281	10
2.6	2	1	µg	mAUM	Cellulose Membrane	0.627	0.249	11
2.6	2	1	µg	mAUM	Cellulose Membrane	0.392	0.311	12
2.6	2	1	µg	mAUM	Cellulose Membrane	0.694	0.285	13
2.6	2	1	µg	mAUM	Cellulose Membrane	0.6	0.262	14
2.6	2	1	µg	mAUM	Cellulose Membrane	0.663	0.237	15
2.6	2	1	µg	mAUM	Cellulose Membrane	0.823	0.262	16
2.6	2	1	µg	mAUM	Cellulose Membrane	1.085	0.161	17
2.6	2	1	µg	mAUM	Cellulose Membrane	0.661	0.339	18
2.6	2	1	µg	mAUM	Cellulose Membrane	0.433	0.257	19
2.6	2	1	µg	mAUM	Cellulose Membrane	0.72	0.281	20
2.6	2	1	µg	mAUM	Cellulose Membrane	0.819	0.217	21
2.6	2	1	µg	mAUM	Cellulose Membrane	0.592	0.266	22
2.6	2	1	µg	mAUM	Cellulose Membrane	0.601	0.254	23

2.6	2	1	µg	mAUM	Cellulose Membrane	0.687	0.257	24
2.6	2	1	µg	mAUM	Cellulose Membrane	0.532	0.237	25
2.7	3	1	µg	mAUM	Cellulose Membrane	0.492	0.299	1
2.7	3	1	µg	mAUM	Cellulose Membrane	0.879	0.309	2
2.7	3	1	µg	mAUM	Cellulose Membrane	0.508	0.299	3
2.7	3	1	µg	mAUM	Cellulose Membrane	0.889	0.276	4
2.7	3	1	µg	mAUM	Cellulose Membrane	0.592	0.305	5
2.7	3	1	µg	mAUM	Cellulose Membrane	0.868	0.346	6
2.7	3	1	µg	mAUM	Cellulose Membrane	0.926	0.197	7
2.7	3	1	µg	mAUM	Cellulose Membrane	0.672	0.231	8
2.7	3	1	µg	mAUM	Cellulose Membrane	0.732	0.302	9
2.7	3	1	µg	mAUM	Cellulose Membrane	0.596	0.225	10
2.7	3	1	µg	mAUM	Cellulose Membrane	0.44	0.348	11
2.7	3	1	µg	mAUM	Cellulose Membrane	0.804	0.261	12
2.7	3	1	µg	mAUM	Cellulose Membrane	0.587	0.322	13
2.7	3	1	µg	mAUM	Cellulose Membrane	0.558	0.352	14
2.7	3	1	µg	mAUM	Cellulose Membrane	0.62	0.321	15
2.7	3	1	µg	mAUM	Cellulose Membrane	0.787	0.328	16
2.7	3	1	µg	mAUM	Cellulose Membrane	0.551	0.247	17
2.7	3	1	µg	mAUM	Cellulose Membrane	0.521	0.339	18
2.7	3	1	µg	mAUM	Cellulose Membrane	0.462	0.24	19
2.7	3	1	µg	mAUM	Cellulose Membrane	0.457	0.226	20
2.7	3	1	µg	mAUM	Cellulose Membrane	0.749	0.36	21
2.7	3	1	µg	mAUM	Cellulose Membrane	0.495	0.334	22
2.7	3	1	µg	mAUM	Cellulose Membrane	0.688	0.331	23

2.7	3	1	µg	mAUM	Cellulose Membrane	0.76	0.372	24
2.7	3	1	µg	mAUM	Cellulose Membrane	0.685	0.26	25
2.8	4	1	µg	mAUM	Cellulose Membrane	0.776	0.262	1
2.8	4	1	µg	mAUM	Cellulose Membrane	0.725	0.28	2
2.8	4	1	µg	mAUM	Cellulose Membrane	0.765	0.234	3
2.8	4	1	µg	mAUM	Cellulose Membrane	0.681	0.277	4
2.8	4	1	µg	mAUM	Cellulose Membrane	0.692	0.249	5
2.8	4	1	µg	mAUM	Cellulose Membrane	0.782	0.291	6
2.8	4	1	µg	mAUM	Cellulose Membrane	0.757	0.25	7
2.8	4	1	µg	mAUM	Cellulose Membrane	0.722	0.271	8
2.8	4	1	µg	mAUM	Cellulose Membrane	0.668	0.273	9
2.8	4	1	µg	mAUM	Cellulose Membrane	0.782	0.268	10
2.8	4	1	µg	mAUM	Cellulose Membrane	0.729	0.244	11
2.8	4	1	µg	mAUM	Cellulose Membrane	0.752	0.297	12
2.8	4	1	µg	mAUM	Cellulose Membrane	0.699	0.25	13
2.8	4	1	µg	mAUM	Cellulose Membrane	0.774	0.311	14
2.8	4	1	µg	mAUM	Cellulose Membrane	0.822	0.305	15
2.8	4	1	µg	mAUM	Cellulose Membrane	0.776	0.343	16
2.8	4	1	µg	mAUM	Cellulose Membrane	0.876	0.327	17
2.8	4	1	µg	mAUM	Cellulose Membrane	0.726	0.309	18
2.8	4	1	µg	mAUM	Cellulose Membrane	0.58	0.265	19
2.8	4	1	µg	mAUM	Cellulose Membrane	0.985	0.237	20
2.8	4	1	µg	mAUM	Cellulose Membrane	0.841	0.244	21
2.8	4	1	µg	mAUM	Cellulose Membrane	0.752	0.306	22
2.8	4	1	µg	mAUM	Cellulose Membrane	0.923	0.347	23

2.8	4	1	µg	mAUM	Cellulose Membrane	0.98	0.304	24
2.8	4	1	µg	mAUM	Cellulose Membrane	0.824	0.378	25
3.1	1	1	µg	mAUM	Silicone	0.628	0.366	1
3.1	1	1	µg	mAUM	Silicone	0.699	0.315	2
3.1	1	1	µg	mAUM	Silicone	0.556	0.388	3
3.1	1	1	µg	mAUM	Silicone	0.744	0.314	4
3.1	1	1	µg	mAUM	Silicone	0.95	0.331	5
3.1	1	1	µg	mAUM	Silicone	0.878	0.38	6
3.1	1	1	µg	mAUM	Silicone	1.018	0.343	7
3.1	1	1	µg	mAUM	Silicone	0.793	0.336	8
3.1	1	1	µg	mAUM	Silicone	0.95	0.329	9
3.1	1	1	µg	mAUM	Silicone	0.661	0.369	10
3.1	1	1	µg	mAUM	Silicone	0.834	0.381	11
3.1	1	1	µg	mAUM	Silicone	0.806	0.429	12
3.1	1	1	µg	mAUM	Silicone	0.698	0.408	13
3.1	1	1	µg	mAUM	Silicone	0.814	0.382	14
3.1	1	1	µg	mAUM	Silicone	1.032	0.341	15
3.1	1	1	µg	mAUM	Silicone	0.988	0.332	16
3.1	1	1	µg	mAUM	Silicone	0.806	0.367	17
3.1	1	1	µg	mAUM	Silicone	1.019	0.421	18
3.1	1	1	µg	mAUM	Silicone	1.096	0.354	19
3.1	1	1	µg	mAUM	Silicone	0.788	0.312	20
3.1	1	1	µg	mAUM	Silicone	1.049	0.403	21
3.1	1	1	µg	mAUM	Silicone	0.718	0.357	22
3.1	1	1	µg	mAUM	Silicone	0.911	0.368	23
3.1	1	1	µg	mAUM	Silicone	0.838	0.373	24
3.1	1	1	µg	mAUM	Silicone	0.697	0.361	25
3.2	2	1	µg	mAUM	Silicone	0.755	0.252	1
3.2	2	1	µg	mAUM	Silicone	0.958	0.269	2
3.2	2	1	µg	mAUM	Silicone	0.659	0.309	3
3.2	2	1	µg	mAUM	Silicone	0.697	0.318	4
3.2	2	1	µg	mAUM	Silicone	1.169	0.303	5
3.2	2	1	µg	mAUM	Silicone	0.903	0.336	6
3.2	2	1	µg	mAUM	Silicone	1.058	0.361	7
3.2	2	1	µg	mAUM	Silicone	0.605	0.364	8
3.2	2	1	µg	mAUM	Silicone	0.546	0.363	9
3.2	2	1	µg	mAUM	Silicone	0.898	0.323	10
3.2	2	1	µg	mAUM	Silicone	1.21	0.323	11
3.2	2	1	µg	mAUM	Silicone	0.84	0.325	12
3.2	2	1	µg	mAUM	Silicone	0.762	0.333	13
3.2	2	1	µg	mAUM	Silicone	0.97	0.345	14
3.2	2	1	µg	mAUM	Silicone	1.293	0.36	15
3.2	2	1	µg	mAUM	Silicone	0.979	0.305	16
3.2	2	1	µg	mAUM	Silicone	1.067	0.395	17
3.2	2	1	µg	mAUM	Silicone	0.984	0.315	18
3.2	2	1	µg	mAUM	Silicone	1.356	0.29	19
3.2	2	1	µg	mAUM	Silicone	0.872	0.343	20
3.2	2	1	µg	mAUM	Silicone	0.907	0.277	21

3.2	2	1	µg	mAUM	Silicone	0.874	0.364	22
3.2	2	1	µg	mAUM	Silicone	1.154	0.351	23
3.2	2	1	µg	mAUM	Silicone	0.869	0.298	24
3.2	2	1	µg	mAUM	Silicone	0.894	0.333	25
3.3	3	1	µg	mAUM	Silicone	1.188	0.504	1
3.3	3	1	µg	mAUM	Silicone	1.055	0.453	2
3.3	3	1	µg	mAUM	Silicone	1.117	0.377	3
3.3	3	1	µg	mAUM	Silicone	1.063	0.456	4
3.3	3	1	µg	mAUM	Silicone	0.976	0.464	5
3.3	3	1	µg	mAUM	Silicone	1.089	0.499	6
3.3	3	1	µg	mAUM	Silicone	1.305	0.439	7
3.3	3	1	µg	mAUM	Silicone	1.478	0.53	8
3.3	3	1	µg	mAUM	Silicone	0.968	0.373	9
3.3	3	1	µg	mAUM	Silicone	1.037	0.497	10
3.3	3	1	µg	mAUM	Silicone	0.988	0.433	11
3.3	3	1	µg	mAUM	Silicone	0.787	0.53	12
3.3	3	1	µg	mAUM	Silicone	1.094	0.532	13
3.3	3	1	µg	mAUM	Silicone	1.485	0.549	14
3.3	3	1	µg	mAUM	Silicone	1.372	0.502	15
3.3	3	1	µg	mAUM	Silicone	1.32	0.388	16
3.3	3	1	µg	mAUM	Silicone	1.184	0.499	17
3.3	3	1	µg	mAUM	Silicone	0.957	0.445	18
3.3	3	1	µg	mAUM	Silicone	0.646	0.443	19
3.3	3	1	µg	mAUM	Silicone	0.714	0.477	20
3.3	3	1	µg	mAUM	Silicone	1.174	0.463	21
3.3	3	1	µg	mAUM	Silicone	0.799	0.466	22
3.3	3	1	µg	mAUM	Silicone	0.775	0.414	23
3.3	3	1	µg	mAUM	Silicone	0.835	0.554	24
3.3	3	1	µg	mAUM	Silicone	1.029	0.54	25
3.4	4	1	µg	mAUM	Silicone	0.82	0.329	1
3.4	4	1	µg	mAUM	Silicone	0.707	0.391	2
3.4	4	1	µg	mAUM	Silicone	0.894	0.344	3
3.4	4	1	µg	mAUM	Silicone	1.134	0.357	4
3.4	4	1	µg	mAUM	Silicone	0.738	0.398	5
3.4	4	1	µg	mAUM	Silicone	0.775	0.441	6
3.4	4	1	µg	mAUM	Silicone	0.696	0.361	7
3.4	4	1	µg	mAUM	Silicone	0.76	0.362	8
3.4	4	1	µg	mAUM	Silicone	0.696	0.391	9
3.4	4	1	µg	mAUM	Silicone	0.893	0.394	10
3.4	4	1	µg	mAUM	Silicone	0.958	0.346	11
3.4	4	1	µg	mAUM	Silicone	0.717	0.387	12
3.4	4	1	µg	mAUM	Silicone	0.914	0.35	13
3.4	4	1	µg	mAUM	Silicone	0.64	0.411	14
3.4	4	1	µg	mAUM	Silicone	1.106	0.349	15
3.4	4	1	µg	mAUM	Silicone	0.85	0.317	16
3.4	4	1	µg	mAUM	Silicone	0.709	0.377	17
3.4	4	1	µg	mAUM	Silicone	0.997	0.353	18
3.4	4	1	µg	mAUM	Silicone	0.875	0.326	19
3.4	4	1	µg	mAUM	Silicone	0.957	0.343	20
3.4	4	1	µg	mAUM	Silicone	0.864	0.337	21

3.4	4	1	µg	mAUM	Silicone	1.336	0.328	22
3.4	4	1	µg	mAUM	Silicone	0.609	0.409	23
3.4	4	1	µg	mAUM	Silicone	0.953	0.319	24
3.4	4	1	µg	mAUM	Silicone	0.987	0.359	25
3.5	1	1	µg	mAUM	Silicone-DLIP	0.807	0.297	1
3.5	1	1	µg	mAUM	Silicone-DLIP	0.917	0.305	2
3.5	1	1	µg	mAUM	Silicone-DLIP	0.639	0.327	3
3.5	1	1	µg	mAUM	Silicone-DLIP	0.941	0.323	4
3.5	1	1	µg	mAUM	Silicone-DLIP	0.749	0.289	5
3.5	1	1	µg	mAUM	Silicone-DLIP	0.951	0.305	6
3.5	1	1	µg	mAUM	Silicone-DLIP	0.976	0.355	7
3.5	1	1	µg	mAUM	Silicone-DLIP	0.805	0.392	8
3.5	1	1	µg	mAUM	Silicone-DLIP	1.022	0.38	9
3.5	1	1	µg	mAUM	Silicone-DLIP	0.755	0.393	10
3.5	1	1	µg	mAUM	Silicone-DLIP	0.547	0.382	11
3.5	1	1	µg	mAUM	Silicone-DLIP	0.796	0.353	12
3.5	1	1	µg	mAUM	Silicone-DLIP	0.714	0.387	13
3.5	1	1	µg	mAUM	Silicone-DLIP	0.811	0.307	14
3.5	1	1	µg	mAUM	Silicone-DLIP	0.8	0.376	15
3.5	1	1	µg	mAUM	Silicone-DLIP	0.738	0.35	16
3.5	1	1	µg	mAUM	Silicone-DLIP	0.677	0.332	17
3.5	1	1	µg	mAUM	Silicone-DLIP	0.961	0.327	18
3.5	1	1	µg	mAUM	Silicone-DLIP	0.63	0.362	19
3.5	1	1	µg	mAUM	Silicone-DLIP	0.762	0.301	20
3.5	1	1	µg	mAUM	Silicone-DLIP	0.62	0.274	21
3.5	1	1	µg	mAUM	Silicone-DLIP	0.567	0.301	22
3.5	1	1	µg	mAUM	Silicone-DLIP	0.68	0.313	23
3.5	1	1	µg	mAUM	Silicone-DLIP	0.918	0.317	24
3.5	1	1	µg	mAUM	Silicone-DLIP	0.737	0.317	25
3.6	2	1	µg	mAUM	Silicone-DLIP	0.959	0.339	1
3.6	2	1	µg	mAUM	Silicone-DLIP	0.687	0.339	2
3.6	2	1	µg	mAUM	Silicone-DLIP	0.765	0.366	3
3.6	2	1	µg	mAUM	Silicone-DLIP	0.466	0.385	4
3.6	2	1	µg	mAUM	Silicone-DLIP	0.467	0.233	5
3.6	2	1	µg	mAUM	Silicone-DLIP	0.775	0.358	6
3.6	2	1	µg	mAUM	Silicone-DLIP	1.034	0.33	7
3.6	2	1	µg	mAUM	Silicone-DLIP	1.164	0.336	8
3.6	2	1	µg	mAUM	Silicone-DLIP	0.89	0.295	9
3.6	2	1	µg	mAUM	Silicone-DLIP	1.318	0.385	10
3.6	2	1	µg	mAUM	Silicone-DLIP	1.016	0.357	11
3.6	2	1	µg	mAUM	Silicone-DLIP	1.571	0.306	12
3.6	2	1	µg	mAUM	Silicone-DLIP	0.754	0.395	13
3.6	2	1	µg	mAUM	Silicone-DLIP	0.928	0.323	14
3.6	2	1	µg	mAUM	Silicone-DLIP	0.877	0.332	15
3.6	2	1	µg	mAUM	Silicone-DLIP	0.334	0.334	16
3.6	2	1	µg	mAUM	Silicone-DLIP	0.332	0.332	17
3.6	2	1	µg	mAUM	Silicone-DLIP	0.391	0.391	18
3.6	2	1	µg	mAUM	Silicone-DLIP	0.379	0.379	19
3.6	2	1	µg	mAUM	Silicone-DLIP	0.313	0.313	20
3.6	2	1	µg	mAUM	Silicone-DLIP	0.329	0.329	21

3.6	2	1	µg	mAUM	Silicone-DLIP	0.632	0.298	22
3.6	2	1	µg	mAUM	Silicone-DLIP	0.761	0.372	23
3.6	2	1	µg	mAUM	Silicone-DLIP	0.586	0.362	24
3.6	2	1	µg	mAUM	Silicone-DLIP	0.714	0.296	25
3.7	3	1	µg	mAUM	Silicone-DLIP	0.952	0.292	1
3.7	3	1	µg	mAUM	Silicone-DLIP	0.554	0.258	2
3.7	3	1	µg	mAUM	Silicone-DLIP	0.641	0.344	3
3.7	3	1	µg	mAUM	Silicone-DLIP	0.714	0.277	4
3.7	3	1	µg	mAUM	Silicone-DLIP	0.626	0.341	5
3.7	3	1	µg	mAUM	Silicone-DLIP	0.88	0.38	6
3.7	3	1	µg	mAUM	Silicone-DLIP	0.538	0.323	7
3.7	3	1	µg	mAUM	Silicone-DLIP	0.449	0.41	8
3.7	3	1	µg	mAUM	Silicone-DLIP	0.433	0.257	9
3.7	3	1	µg	mAUM	Silicone-DLIP	0.679	0.4	10
3.7	3	1	µg	mAUM	Silicone-DLIP	0.762	0.323	11
3.7	3	1	µg	mAUM	Silicone-DLIP	0.747	0.319	12
3.7	3	1	µg	mAUM	Silicone-DLIP	1.022	0.407	13
3.7	3	1	µg	mAUM	Silicone-DLIP	0.966	0.362	14
3.7	3	1	µg	mAUM	Silicone-DLIP	0.749	0.298	15
3.7	3	1	µg	mAUM	Silicone-DLIP	0.624	0.381	16
3.7	3	1	µg	mAUM	Silicone-DLIP	0.725	0.513	17
3.7	3	1	µg	mAUM	Silicone-DLIP	0.761	0.281	18
3.7	3	1	µg	mAUM	Silicone-DLIP	0.685	0.28	19
3.7	3	1	µg	mAUM	Silicone-DLIP	0.678	0.352	20
3.7	3	1	µg	mAUM	Silicone-DLIP	1.067	0.35	21
3.7	3	1	µg	mAUM	Silicone-DLIP	0.639	0.285	22
3.7	3	1	µg	mAUM	Silicone-DLIP	0.707	0.316	23
3.7	3	1	µg	mAUM	Silicone-DLIP	0.592	0.368	24
3.7	3	1	µg	mAUM	Silicone-DLIP	0.533	0.269	25
3.8	4	1	µg	mAUM	Silicone-DLIP	0.728	0.317	1
3.8	4	1	µg	mAUM	Silicone-DLIP	0.694	0.246	2
3.8	4	1	µg	mAUM	Silicone-DLIP	0.523	0.269	3
3.8	4	1	µg	mAUM	Silicone-DLIP	0.458	0.25	4
3.8	4	1	µg	mAUM	Silicone-DLIP	0.593	0.26	5
3.8	4	1	µg	mAUM	Silicone-DLIP	0.933	0.338	6
3.8	4	1	µg	mAUM	Silicone-DLIP	0.683	0.254	7
3.8	4	1	µg	mAUM	Silicone-DLIP	0.569	0.36	8
3.8	4	1	µg	mAUM	Silicone-DLIP	0.569	0.417	9
3.8	4	1	µg	mAUM	Silicone-DLIP	0.498	0.268	10
3.8	4	1	µg	mAUM	Silicone-DLIP	0.482	0.286	11
3.8	4	1	µg	mAUM	Silicone-DLIP	0.675	0.305	12
3.8	4	1	µg	mAUM	Silicone-DLIP	0.629	0.336	13
3.8	4	1	µg	mAUM	Silicone-DLIP	0.787	0.256	14
3.8	4	1	µg	mAUM	Silicone-DLIP	0.539	0.316	15
3.8	4	1	µg	mAUM	Silicone-DLIP	0.523	0.273	16
3.8	4	1	µg	mAUM	Silicone-DLIP	0.431	0.375	17
3.8	4	1	µg	mAUM	Silicone-DLIP	0.694	0.313	18
3.8	4	1	µg	mAUM	Silicone-DLIP	0.533	0.323	19
3.8	4	1	µg	mAUM	Silicone-DLIP	0.857	0.372	20
3.8	4	1	µg	mAUM	Silicone-DLIP	0.594	0.419	21

3.8	4	1	µg	mAUM	Silicone-DLIP	0.907	0.335	22
3.8	4	1	µg	mAUM	Silicone-DLIP	0.647	0.445	23
3.8	4	1	µg	mAUM	Silicone-DLIP	0.646	0.315	24
3.8	4	1	µg	mAUM	Silicone-DLIP	0.606	0.295	25
8.5	1	2	µg	mAUM	Cellulose Membrane	0.713	0.33	1
8.5	1	2	µg	mAUM	Cellulose Membrane	0.66	0.342	2
8.5	1	2	µg	mAUM	Cellulose Membrane	0.563	0.321	3
8.5	1	2	µg	mAUM	Cellulose Membrane	0.933	0.342	4
8.5	1	2	µg	mAUM	Cellulose Membrane	0.768	0.313	5
8.5	1	2	µg	mAUM	Cellulose Membrane	0.829	0.284	6
8.5	1	2	µg	mAUM	Cellulose Membrane	0.733	0.276	7
8.5	1	2	µg	mAUM	Cellulose Membrane	0.837	0.334	8
8.5	1	2	µg	mAUM	Cellulose Membrane	0.733	0.269	9
8.5	1	2	µg	mAUM	Cellulose Membrane	0.694	0.307	10
8.5	1	2	µg	mAUM	Cellulose Membrane	0.822	0.338	11
8.5	1	2	µg	mAUM	Cellulose Membrane	0.748	0.284	12
8.5	1	2	µg	mAUM	Cellulose Membrane	0.741	0.33	13
8.5	1	2	µg	mAUM	Cellulose Membrane	0.688	0.297	14
8.5	1	2	µg	mAUM	Cellulose Membrane	0.92	0.321	15
8.5	1	2	µg	mAUM	Cellulose Membrane	0.751	0.321	16
8.5	1	2	µg	mAUM	Cellulose Membrane	0.644	0.294	17
8.5	1	2	µg	mAUM	Cellulose Membrane	0.817	0.33	18
8.5	1	2	µg	mAUM	Cellulose Membrane	0.761	0.361	19
8.5	1	2	µg	mAUM	Cellulose Membrane	0.934	0.381	20
8.5	1	2	µg	mAUM	Cellulose Membrane	0.697	0.365	21
8.5	1	2	µg	mAUM	Cellulose Membrane	0.73	0.365	22
8.5	1	2	µg	mAUM	Cellulose Membrane	0.72	0.386	23

8.5	1	2	µg	mAUM	Cellulose Membrane	0.727	0.298	24
8.5	1	2	µg	mAUM	Cellulose Membrane	0.704	0.295	25
8.6	2	2	µg	mAUM	Cellulose Membrane	0.649	0.303	1
8.6	2	2	µg	mAUM	Cellulose Membrane	0.685	0.305	2
8.6	2	2	µg	mAUM	Cellulose Membrane	0.958	0.276	3
8.6	2	2	µg	mAUM	Cellulose Membrane	0.741	0.309	4
8.6	2	2	µg	mAUM	Cellulose Membrane	0.963	0.348	5
8.6	2	2	µg	mAUM	Cellulose Membrane	0.653	0.308	6
8.6	2	2	µg	mAUM	Cellulose Membrane	0.628	0.348	7
8.6	2	2	µg	mAUM	Cellulose Membrane	0.814	0.365	8
8.6	2	2	µg	mAUM	Cellulose Membrane	0.719	0.322	9
8.6	2	2	µg	mAUM	Cellulose Membrane	0.841	0.305	10
8.6	2	2	µg	mAUM	Cellulose Membrane	0.77	0.407	11
8.6	2	2	µg	mAUM	Cellulose Membrane	0.913	0.307	12
8.6	2	2	µg	mAUM	Cellulose Membrane	0.945	0.432	13
8.6	2	2	µg	mAUM	Cellulose Membrane	0.898	0.371	14
8.6	2	2	µg	mAUM	Cellulose Membrane	0.949	0.356	15
8.6	2	2	µg	mAUM	Cellulose Membrane	0.636	0.315	16
8.6	2	2	µg	mAUM	Cellulose Membrane	0.728	0.284	17
8.6	2	2	µg	mAUM	Cellulose Membrane	0.961	0.311	18
8.6	2	2	µg	mAUM	Cellulose Membrane	0.69	0.341	19
8.6	2	2	µg	mAUM	Cellulose Membrane	0.876	0.312	20
8.6	2	2	µg	mAUM	Cellulose Membrane	0.845	0.335	21
8.6	2	2	µg	mAUM	Cellulose Membrane	0.809	0.328	22
8.6	2	2	µg	mAUM	Cellulose Membrane	0.779	0.362	23

8.6	2	2	µg	mAUM	Cellulose Membrane	0.768	0.288	24
8.6	2	2	µg	mAUM	Cellulose Membrane	0.704	0.276	25
8.7	3	2	µg	mAUM	Cellulose Membrane	1.101	0.297	1
8.7	3	2	µg	mAUM	Cellulose Membrane	0.638	0.332	2
8.7	3	2	µg	mAUM	Cellulose Membrane	0.594	0.367	3
8.7	3	2	µg	mAUM	Cellulose Membrane	0.71	0.252	4
8.7	3	2	µg	mAUM	Cellulose Membrane	0.583	0.336	5
8.7	3	2	µg	mAUM	Cellulose Membrane	0.585	0.302	6
8.7	3	2	µg	mAUM	Cellulose Membrane	0.768	0.36	7
8.7	3	2	µg	mAUM	Cellulose Membrane	1.053	0.299	8
8.7	3	2	µg	mAUM	Cellulose Membrane	1.008	0.271	9
8.7	3	2	µg	mAUM	Cellulose Membrane	0.623	0.321	10
8.7	3	2	µg	mAUM	Cellulose Membrane	0.647	0.348	11
8.7	3	2	µg	mAUM	Cellulose Membrane	1.015	0.399	12
8.7	3	2	µg	mAUM	Cellulose Membrane	0.9	0.41	13
8.7	3	2	µg	mAUM	Cellulose Membrane	0.679	0.321	14
8.7	3	2	µg	mAUM	Cellulose Membrane	0.847	0.329	15
8.7	3	2	µg	mAUM	Cellulose Membrane	0.774	0.305	16
8.7	3	2	µg	mAUM	Cellulose Membrane	0.999	0.3	17
8.7	3	2	µg	mAUM	Cellulose Membrane	0.93	0.331	18
8.7	3	2	µg	mAUM	Cellulose Membrane	0.628	0.319	19
8.7	3	2	µg	mAUM	Cellulose Membrane	0.702	0.276	20
8.7	3	2	µg	mAUM	Cellulose Membrane	0.911	0.281	21
8.7	3	2	µg	mAUM	Cellulose Membrane	0.554	0.296	22
8.7	3	2	µg	mAUM	Cellulose Membrane	0.701	0.323	23

8.7	3	2	µg	mAUM	Cellulose Membrane	1.202	0.323	24
8.7	3	2	µg	mAUM	Cellulose Membrane	0.963	0.334	25
8.8	4	2	µg	mAUM	Cellulose Membrane	0.738	0.361	1
8.8	4	2	µg	mAUM	Cellulose Membrane	0.831	0.308	2
8.8	4	2	µg	mAUM	Cellulose Membrane	0.521	0.369	3
8.8	4	2	µg	mAUM	Cellulose Membrane	0.801	0.303	4
8.8	4	2	µg	mAUM	Cellulose Membrane	0.728	0.303	5
8.8	4	2	µg	mAUM	Cellulose Membrane	0.681	0.331	6
8.8	4	2	µg	mAUM	Cellulose Membrane	1.035	0.334	7
8.8	4	2	µg	mAUM	Cellulose Membrane	0.768	0.269	8
8.8	4	2	µg	mAUM	Cellulose Membrane	0.668	0.267	9
8.8	4	2	µg	mAUM	Cellulose Membrane	0.771	0.278	10
8.8	4	2	µg	mAUM	Cellulose Membrane	0.668	0.266	11
8.8	4	2	µg	mAUM	Cellulose Membrane	0.541	0.26	12
8.8	4	2	µg	mAUM	Cellulose Membrane	0.99	0.245	13
8.8	4	2	µg	mAUM	Cellulose Membrane	0.627	0.328	14
8.8	4	2	µg	mAUM	Cellulose Membrane	0.715	0.323	15
8.8	4	2	µg	mAUM	Cellulose Membrane	0.781	0.305	16
8.8	4	2	µg	mAUM	Cellulose Membrane	0.746	0.346	17
8.8	4	2	µg	mAUM	Cellulose Membrane	0.733	0.343	18
8.8	4	2	µg	mAUM	Cellulose Membrane	0.647	0.355	19
8.8	4	2	µg	mAUM	Cellulose Membrane	0.821	0.29	20
8.8	4	2	µg	mAUM	Cellulose Membrane	0.653	0.341	21
8.8	4	2	µg	mAUM	Cellulose Membrane	0.754	0.364	22
8.8	4	2	µg	mAUM	Cellulose Membrane	0.921	0.408	23

8.8	4	2	µg	mAUM	Cellulose Membrane	0.88	0.366	24
8.8	4	2	µg	mAUM	Cellulose Membrane	0.716	0.328	25
9.1	1	2	µg	mAUM	Silicone	0.973	0.401	1
9.1	1	2	µg	mAUM	Silicone	0.776	0.283	2
9.1	1	2	µg	mAUM	Silicone	1.125	0.257	3
9.1	1	2	µg	mAUM	Silicone	0.892	0.347	4
9.1	1	2	µg	mAUM	Silicone	0.775	0.279	5
9.1	1	2	µg	mAUM	Silicone	0.938	0.361	6
9.1	1	2	µg	mAUM	Silicone	0.718	0.272	7
9.1	1	2	µg	mAUM	Silicone	0.878	0.339	8
9.1	1	2	µg	mAUM	Silicone	0.483	0.305	9
9.1	1	2	µg	mAUM	Silicone	1.158	0.305	10
9.1	1	2	µg	mAUM	Silicone	0.758	0.371	11
9.1	1	2	µg	mAUM	Silicone	0.759	0.273	12
9.1	1	2	µg	mAUM	Silicone	1.262	0.311	13
9.1	1	2	µg	mAUM	Silicone	0.608	0.332	14
9.1	1	2	µg	mAUM	Silicone	0.599	0.378	15
9.1	1	2	µg	mAUM	Silicone	0.963	0.311	16
9.1	1	2	µg	mAUM	Silicone	0.676	0.321	17
9.1	1	2	µg	mAUM	Silicone	0.808	0.376	18
9.1	1	2	µg	mAUM	Silicone	0.661	0.316	19
9.1	1	2	µg	mAUM	Silicone	0.742	0.332	20
9.1	1	2	µg	mAUM	Silicone	0.726	0.332	21
9.1	1	2	µg	mAUM	Silicone	0.757	0.238	22
9.1	1	2	µg	mAUM	Silicone	0.958	0.315	23
9.1	1	2	µg	mAUM	Silicone	0.973	0.413	24
9.1	1	2	µg	mAUM	Silicone	1.201	0.391	25
9.2	2	2	µg	mAUM	Silicone	0.791	0.303	1
9.2	2	2	µg	mAUM	Silicone	0.735	0.352	2
9.2	2	2	µg	mAUM	Silicone	0.774	0.279	3
9.2	2	2	µg	mAUM	Silicone	0.783	0.356	4
9.2	2	2	µg	mAUM	Silicone	0.613	0.304	5
9.2	2	2	µg	mAUM	Silicone	0.674	0.354	6
9.2	2	2	µg	mAUM	Silicone	0.623	0.255	7
9.2	2	2	µg	mAUM	Silicone	0.957	0.279	8
9.2	2	2	µg	mAUM	Silicone	0.774	0.327	9
9.2	2	2	µg	mAUM	Silicone	0.91	0.295	10
9.2	2	2	µg	mAUM	Silicone	0.752	0.26	11
9.2	2	2	µg	mAUM	Silicone	0.653	0.346	12
9.2	2	2	µg	mAUM	Silicone	0.66	0.292	13
9.2	2	2	µg	mAUM	Silicone	0.777	0.282	14
9.2	2	2	µg	mAUM	Silicone	0.925	0.333	15
9.2	2	2	µg	mAUM	Silicone	0.615	0.308	16
9.2	2	2	µg	mAUM	Silicone	1.016	0.294	17
9.2	2	2	µg	mAUM	Silicone	0.599	0.275	18
9.2	2	2	µg	mAUM	Silicone	0.804	0.372	19
9.2	2	2	µg	mAUM	Silicone	0.818	0.321	20
9.2	2	2	µg	mAUM	Silicone	0.88	0.257	21

9.2	2	2	µg	mAUM	Silicone	0.706	0.33	22
9.2	2	2	µg	mAUM	Silicone	0.796	0.309	23
9.2	2	2	µg	mAUM	Silicone	0.872	0.325	24
9.2	2	2	µg	mAUM	Silicone	0.828	0.277	25
9.3	3	2	µg	mAUM	Silicone	0.71	0.334	1
9.3	3	2	µg	mAUM	Silicone	0.796	0.33	2
9.3	3	2	µg	mAUM	Silicone	0.821	0.376	3
9.3	3	2	µg	mAUM	Silicone	0.815	0.321	4
9.3	3	2	µg	mAUM	Silicone	0.948	0.383	5
9.3	3	2	µg	mAUM	Silicone	0.756	0.375	6
9.3	3	2	µg	mAUM	Silicone	0.941	0.327	7
9.3	3	2	µg	mAUM	Silicone	0.662	0.359	8
9.3	3	2	µg	mAUM	Silicone	0.726	0.371	9
9.3	3	2	µg	mAUM	Silicone	0.785	0.371	10
9.3	3	2	µg	mAUM	Silicone	0.764	0.323	11
9.3	3	2	µg	mAUM	Silicone	0.864	0.344	12
9.3	3	2	µg	mAUM	Silicone	0.653	0.387	13
9.3	3	2	µg	mAUM	Silicone	0.864	0.358	14
9.3	3	2	µg	mAUM	Silicone	0.836	0.387	15
9.3	3	2	µg	mAUM	Silicone	0.865	0.346	16
9.3	3	2	µg	mAUM	Silicone	0.713	0.361	17
9.3	3	2	µg	mAUM	Silicone	0.851	0.331	18
9.3	3	2	µg	mAUM	Silicone	0.578	0.378	19
9.3	3	2	µg	mAUM	Silicone	0.778	0.315	20
9.3	3	2	µg	mAUM	Silicone	0.743	0.341	21
9.3	3	2	µg	mAUM	Silicone	0.698	0.406	22
9.3	3	2	µg	mAUM	Silicone	0.746	0.338	23
9.3	3	2	µg	mAUM	Silicone	1.143	0.348	24
9.3	3	2	µg	mAUM	Silicone	0.952	0.365	25
9.4	4	2	µg	mAUM	Silicone	0.682	0.334	1
9.4	4	2	µg	mAUM	Silicone	0.823	0.342	2
9.4	4	2	µg	mAUM	Silicone	0.582	0.323	3
9.4	4	2	µg	mAUM	Silicone	0.813	0.315	4
9.4	4	2	µg	mAUM	Silicone	0.853	0.264	5
9.4	4	2	µg	mAUM	Silicone	0.804	0.321	6
9.4	4	2	µg	mAUM	Silicone	0.801	0.305	7
9.4	4	2	µg	mAUM	Silicone	1.091	0.282	8
9.4	4	2	µg	mAUM	Silicone	0.643	0.351	9
9.4	4	2	µg	mAUM	Silicone	0.799	0.283	10
9.4	4	2	µg	mAUM	Silicone	0.712	0.415	11
9.4	4	2	µg	mAUM	Silicone	0.85	0.258	12
9.4	4	2	µg	mAUM	Silicone	0.97	0.395	13
9.4	4	2	µg	mAUM	Silicone	1.175	0.39	14
9.4	4	2	µg	mAUM	Silicone	0.861	0.343	15
9.4	4	2	µg	mAUM	Silicone	0.547	0.332	16
9.4	4	2	µg	mAUM	Silicone	0.99	0.357	17
9.4	4	2	µg	mAUM	Silicone	1.071	0.276	18
9.4	4	2	µg	mAUM	Silicone	1.098	0.297	19
9.4	4	2	µg	mAUM	Silicone	1.208	0.324	20
9.4	4	2	µg	mAUM	Silicone	0.873	0.292	21

9.4	4	2	µg	mAUM	Silicone	0.902	0.339	22
9.4	4	2	µg	mAUM	Silicone	0.752	0.317	23
9.4	4	2	µg	mAUM	Silicone	0.968	0.265	24
9.4	4	2	µg	mAUM	Silicone	1.101	0.351	25
9.5	1	2	µg	mAUM	Silicone-DLIP	0.836	0.321	1
9.5	1	2	µg	mAUM	Silicone-DLIP	0.905	0.353	2
9.5	1	2	µg	mAUM	Silicone-DLIP	0.674	0.387	3
9.5	1	2	µg	mAUM	Silicone-DLIP	0.737	0.299	4
9.5	1	2	µg	mAUM	Silicone-DLIP	0.738	0.338	5
9.5	1	2	µg	mAUM	Silicone-DLIP	0.806	0.347	6
9.5	1	2	µg	mAUM	Silicone-DLIP	1.019	0.331	7
9.5	1	2	µg	mAUM	Silicone-DLIP	0.682	0.379	8
9.5	1	2	µg	mAUM	Silicone-DLIP	0.712	0.314	9
9.5	1	2	µg	mAUM	Silicone-DLIP	1.23	0.298	10
9.5	1	2	µg	mAUM	Silicone-DLIP	0.825	0.384	11
9.5	1	2	µg	mAUM	Silicone-DLIP	1.139	0.367	12
9.5	1	2	µg	mAUM	Silicone-DLIP	0.718	0.367	13
9.5	1	2	µg	mAUM	Silicone-DLIP	0.586	0.337	14
9.5	1	2	µg	mAUM	Silicone-DLIP	0.916	0.341	15
9.5	1	2	µg	mAUM	Silicone-DLIP	0.839	0.355	16
9.5	1	2	µg	mAUM	Silicone-DLIP	1.009	0.33	17
9.5	1	2	µg	mAUM	Silicone-DLIP	0.567	0.346	18
9.5	1	2	µg	mAUM	Silicone-DLIP	0.581	0.346	19
9.5	1	2	µg	mAUM	Silicone-DLIP	0.915	0.309	20
9.5	1	2	µg	mAUM	Silicone-DLIP	0.912	0.346	21
9.5	1	2	µg	mAUM	Silicone-DLIP	0.781	0.344	22
9.5	1	2	µg	mAUM	Silicone-DLIP	0.754	0.379	23
9.5	1	2	µg	mAUM	Silicone-DLIP	0.866	0.322	24
9.5	1	2	µg	mAUM	Silicone-DLIP	0.964	0.352	25
9.6	2	2	µg	mAUM	Silicone-DLIP	0.863	0.311	1
9.6	2	2	µg	mAUM	Silicone-DLIP	1.088	0.319	2
9.6	2	2	µg	mAUM	Silicone-DLIP	0.958	0.399	3
9.6	2	2	µg	mAUM	Silicone-DLIP	1.242	0.36	4
9.6	2	2	µg	mAUM	Silicone-DLIP	0.931	0.368	5
9.6	2	2	µg	mAUM	Silicone-DLIP	0.695	0.315	6
9.6	2	2	µg	mAUM	Silicone-DLIP	1.253	0.323	7
9.6	2	2	µg	mAUM	Silicone-DLIP	0.713	0.305	8
9.6	2	2	µg	mAUM	Silicone-DLIP	1.037	0.396	9
9.6	2	2	µg	mAUM	Silicone-DLIP	1.031	0.318	10
9.6	2	2	µg	mAUM	Silicone-DLIP	0.928	0.305	11
9.6	2	2	µg	mAUM	Silicone-DLIP	0.747	0.316	12
9.6	2	2	µg	mAUM	Silicone-DLIP	0.741	0.362	13
9.6	2	2	µg	mAUM	Silicone-DLIP	1.007	0.449	14
9.6	2	2	µg	mAUM	Silicone-DLIP	0.726	0.268	15
9.6	2	2	µg	mAUM	Silicone-DLIP	0.908	0.329	16
9.6	2	2	µg	mAUM	Silicone-DLIP	0.732	0.267	17
9.6	2	2	µg	mAUM	Silicone-DLIP	0.91	0.306	18
9.6	2	2	µg	mAUM	Silicone-DLIP	1.235	0.283	19
9.6	2	2	µg	mAUM	Silicone-DLIP	1.357	0.193	20
9.6	2	2	µg	mAUM	Silicone-DLIP	1.053	0.322	21

9.6	2	2	µg	mAUM	Silicone-DLIP	1.019	0.347	22
9.6	2	2	µg	mAUM	Silicone-DLIP	0.745	0.26	23
9.6	2	2	µg	mAUM	Silicone-DLIP	1.083	0.335	24
9.6	2	2	µg	mAUM	Silicone-DLIP	0.918	0.326	25
9.7	3	2	µg	mAUM	Silicone-DLIP	0.915	0.365	1
9.7	3	2	µg	mAUM	Silicone-DLIP	0.797	0.269	2
9.7	3	2	µg	mAUM	Silicone-DLIP	0.698	0.38	3
9.7	3	2	µg	mAUM	Silicone-DLIP	0.771	0.383	4
9.7	3	2	µg	mAUM	Silicone-DLIP	0.65	0.307	5
9.7	3	2	µg	mAUM	Silicone-DLIP	0.9	0.396	6
9.7	3	2	µg	mAUM	Silicone-DLIP	0.704	0.332	7
9.7	3	2	µg	mAUM	Silicone-DLIP	0.942	0.407	8
9.7	3	2	µg	mAUM	Silicone-DLIP	0.948	0.364	9
9.7	3	2	µg	mAUM	Silicone-DLIP	1.149	0.375	10
9.7	3	2	µg	mAUM	Silicone-DLIP	0.73	0.332	11
9.7	3	2	µg	mAUM	Silicone-DLIP	0.727	0.337	12
9.7	3	2	µg	mAUM	Silicone-DLIP	0.682	0.334	13
9.7	3	2	µg	mAUM	Silicone-DLIP	0.979	0.34	14
9.7	3	2	µg	mAUM	Silicone-DLIP	0.69	0.367	15
9.7	3	2	µg	mAUM	Silicone-DLIP	0.894	0.311	16
9.7	3	2	µg	mAUM	Silicone-DLIP	0.811	0.293	17
9.7	3	2	µg	mAUM	Silicone-DLIP	0.792	0.415	18
9.7	3	2	µg	mAUM	Silicone-DLIP	0.838	0.328	19
9.7	3	2	µg	mAUM	Silicone-DLIP	0.945	0.342	20
9.7	3	2	µg	mAUM	Silicone-DLIP	0.885	0.381	21
9.7	3	2	µg	mAUM	Silicone-DLIP	1.004	0.323	22
9.7	3	2	µg	mAUM	Silicone-DLIP	0.927	0.367	23
9.7	3	2	µg	mAUM	Silicone-DLIP	0.813	0.346	24
9.7	3	2	µg	mAUM	Silicone-DLIP	1.03	0.313	25
9.8	4	2	µg	mAUM	Silicone-DLIP	1.059	0.315	1
9.8	4	2	µg	mAUM	Silicone-DLIP	0.833	0.353	2
9.8	4	2	µg	mAUM	Silicone-DLIP	0.971	0.328	3
9.8	4	2	µg	mAUM	Silicone-DLIP	0.883	0.252	4
9.8	4	2	µg	mAUM	Silicone-DLIP	1.257	0.341	5
9.8	4	2	µg	mAUM	Silicone-DLIP	1.029	0.295	6
9.8	4	2	µg	mAUM	Silicone-DLIP	0.955	0.35	7
9.8	4	2	µg	mAUM	Silicone-DLIP	1.087	0.228	8
9.8	4	2	µg	mAUM	Silicone-DLIP	0.869	0.297	9
9.8	4	2	µg	mAUM	Silicone-DLIP	0.791	0.342	10
9.8	4	2	µg	mAUM	Silicone-DLIP	0.861	0.327	11
9.8	4	2	µg	mAUM	Silicone-DLIP	0.87	0.31	12
9.8	4	2	µg	mAUM	Silicone-DLIP	0.839	0.339	13
9.8	4	2	µg	mAUM	Silicone-DLIP	0.802	0.378	14
9.8	4	2	µg	mAUM	Silicone-DLIP	0.683	0.379	15
9.8	4	2	µg	mAUM	Silicone-DLIP	0.62	0.369	16
9.8	4	2	µg	mAUM	Silicone-DLIP	0.875	0.282	17
9.8	4	2	µg	mAUM	Silicone-DLIP	0.52	0.317	18
9.8	4	2	µg	mAUM	Silicone-DLIP	0.672	0.327	19
9.8	4	2	µg	mAUM	Silicone-DLIP	1.034	0.321	20
9.8	4	2	µg	mAUM	Silicone-DLIP	0.952	0.375	21

9.8	4	2	µg	mAUM	Silicone-DLIP	0.727	0.271	22
9.8	4	2	µg	mAUM	Silicone-DLIP	0.656	0.286	23
9.8	4	2	µg	mAUM	Silicone-DLIP	1.148	0.365	24
9.8	4	2	µg	mAUM	Silicone-DLIP	0.851	0.234	25
14.5	1	3	µg	mAUM	Cellulose Membrane	0.807	0.252	1
14.5	1	3	µg	mAUM	Cellulose Membrane	0.687	0.305	2
14.5	1	3	µg	mAUM	Cellulose Membrane	1.01	0.257	3
14.5	1	3	µg	mAUM	Cellulose Membrane	1.097	0.33	4
14.5	1	3	µg	mAUM	Cellulose Membrane	1.115	0.329	5
14.5	1	3	µg	mAUM	Cellulose Membrane	0.618	0.233	6
14.5	1	3	µg	mAUM	Cellulose Membrane	0.786	0.269	7
14.5	1	3	µg	mAUM	Cellulose Membrane	0.795	0.306	8
14.5	1	3	µg	mAUM	Cellulose Membrane	0.95	0.323	9
14.5	1	3	µg	mAUM	Cellulose Membrane	1.107	0.24	10
14.5	1	3	µg	mAUM	Cellulose Membrane	0.601	0.345	11
14.5	1	3	µg	mAUM	Cellulose Membrane	0.625	0.306	12
14.5	1	3	µg	mAUM	Cellulose Membrane	0.642	0.306	13
14.5	1	3	µg	mAUM	Cellulose Membrane	0.737	0.286	14
14.5	1	3	µg	mAUM	Cellulose Membrane	0.861	0.332	15
14.5	1	3	µg	mAUM	Cellulose Membrane	0.779	0.387	16
14.5	1	3	µg	mAUM	Cellulose Membrane	1.106	0.319	17
14.5	1	3	µg	mAUM	Cellulose Membrane	0.914	0.327	18
14.5	1	3	µg	mAUM	Cellulose Membrane	0.923	0.343	19
14.5	1	3	µg	mAUM	Cellulose Membrane	0.982	0.323	20
14.5	1	3	µg	mAUM	Cellulose Membrane	0.92	0.294	21
14.5	1	3	µg	mAUM	Cellulose Membrane	0.968	0.325	22
14.5	1	3	µg	mAUM	Cellulose Membrane	0.86	0.325	23

14.5	1	3	µg	mAUM	Cellulose Membrane	0.803	0.299	24
14.5	1	3	µg	mAUM	Cellulose Membrane	0.935	0.32	25
14.6	2	3	µg	mAUM	Cellulose Membrane	0.807	0.252	1
14.6	2	3	µg	mAUM	Cellulose Membrane	0.687	0.305	2
14.6	2	3	µg	mAUM	Cellulose Membrane	1.01	0.257	3
14.6	2	3	µg	mAUM	Cellulose Membrane	1.097	0.33	4
14.6	2	3	µg	mAUM	Cellulose Membrane	1.115	0.329	5
14.6	2	3	µg	mAUM	Cellulose Membrane	0.618	0.233	6
14.6	2	3	µg	mAUM	Cellulose Membrane	0.786	0.269	7
14.6	2	3	µg	mAUM	Cellulose Membrane	0.795	0.306	8
14.6	2	3	µg	mAUM	Cellulose Membrane	0.95	0.323	9
14.6	2	3	µg	mAUM	Cellulose Membrane	1.107	0.24	10
14.6	2	3	µg	mAUM	Cellulose Membrane	0.601	0.345	11
14.6	2	3	µg	mAUM	Cellulose Membrane	1.213	0.27	12
14.6	2	3	µg	mAUM	Cellulose Membrane	0.625	0.306	13
14.6	2	3	µg	mAUM	Cellulose Membrane	0.642	0.306	14
14.6	2	3	µg	mAUM	Cellulose Membrane	0.737	0.286	15
14.6	2	3	µg	mAUM	Cellulose Membrane	0.861	0.332	16
14.6	2	3	µg	mAUM	Cellulose Membrane	0.779	0.387	17
14.6	2	3	µg	mAUM	Cellulose Membrane	1.106	0.319	18
14.6	2	3	µg	mAUM	Cellulose Membrane	0.914	0.327	19
14.6	2	3	µg	mAUM	Cellulose Membrane	0.923	0.343	20
14.6	2	3	µg	mAUM	Cellulose Membrane	0.982	0.323	21
14.6	2	3	µg	mAUM	Cellulose Membrane	0.92	0.294	22
14.6	2	3	µg	mAUM	Cellulose Membrane	0.968	0.325	23

14.6	2	3	µg	mAUM	Cellulose Membrane	1.248	0.264	24
14.6	2	3	µg	mAUM	Cellulose Membrane	0.86	0.325	25
14.7	3	3	µg	mAUM	Cellulose Membrane	1.062	0.323	1
14.7	3	3	µg	mAUM	Cellulose Membrane	0.819	0.339	2
14.7	3	3	µg	mAUM	Cellulose Membrane	0.973	0.319	3
14.7	3	3	µg	mAUM	Cellulose Membrane	0.755	0.325	4
14.7	3	3	µg	mAUM	Cellulose Membrane	0.764	0.33	5
14.7	3	3	µg	mAUM	Cellulose Membrane	0.997	0.388	6
14.7	3	3	µg	mAUM	Cellulose Membrane	0.798	0.384	7
14.7	3	3	µg	mAUM	Cellulose Membrane	0.777	0.362	8
14.7	3	3	µg	mAUM	Cellulose Membrane	1.054	0.327	9
14.7	3	3	µg	mAUM	Cellulose Membrane	0.774	0.294	10
14.7	3	3	µg	mAUM	Cellulose Membrane	0.855	0.31	11
14.7	3	3	µg	mAUM	Cellulose Membrane	0.692	0.295	12
14.7	3	3	µg	mAUM	Cellulose Membrane	0.872	0.302	13
14.7	3	3	µg	mAUM	Cellulose Membrane	0.834	0.337	14
14.7	3	3	µg	mAUM	Cellulose Membrane	0.783	0.355	15
14.7	3	3	µg	mAUM	Cellulose Membrane	0.685	0.343	16
14.7	3	3	µg	mAUM	Cellulose Membrane	1.251	0.349	17
14.7	3	3	µg	mAUM	Cellulose Membrane	1.191	0.397	18
14.7	3	3	µg	mAUM	Cellulose Membrane	1.213	0.333	19
14.7	3	3	µg	mAUM	Cellulose Membrane	1.095	0.313	20
14.7	3	3	µg	mAUM	Cellulose Membrane	1.016	0.343	21
14.7	3	3	µg	mAUM	Cellulose Membrane	1.004	0.344	22
14.7	3	3	µg	mAUM	Cellulose Membrane	0.85	0.297	23

14.7	3	3	µg	mAUM	Cellulose Membrane	1.089	0.329	24
14.7	3	3	µg	mAUM	Cellulose Membrane	0.717	0.289	25
14.8	4	3	µg	mAUM	Cellulose Membrane	0.736	0.33	1
14.8	4	3	µg	mAUM	Cellulose Membrane	1.003	0.318	2
14.8	4	3	µg	mAUM	Cellulose Membrane	0.749	0.293	3
14.8	4	3	µg	mAUM	Cellulose Membrane	0.83	0.306	4
14.8	4	3	µg	mAUM	Cellulose Membrane	0.858	0.305	5
14.8	4	3	µg	mAUM	Cellulose Membrane	0.816	0.278	6
14.8	4	3	µg	mAUM	Cellulose Membrane	0.902	0.339	7
14.8	4	3	µg	mAUM	Cellulose Membrane	0.817	0.344	8
14.8	4	3	µg	mAUM	Cellulose Membrane	0.785	0.338	9
14.8	4	3	µg	mAUM	Cellulose Membrane	0.86	0.326	10
14.8	4	3	µg	mAUM	Cellulose Membrane	0.722	0.329	11
14.8	4	3	µg	mAUM	Cellulose Membrane	0.853	0.306	12
14.8	4	3	µg	mAUM	Cellulose Membrane	0.661	0.301	13
14.8	4	3	µg	mAUM	Cellulose Membrane	1.008	0.404	14
14.8	4	3	µg	mAUM	Cellulose Membrane	0.763	0.37	15
14.8	4	3	µg	mAUM	Cellulose Membrane	0.748	0.344	16
14.8	4	3	µg	mAUM	Cellulose Membrane	1.067	0.288	17
14.8	4	3	µg	mAUM	Cellulose Membrane	0.77	0.293	18
14.8	4	3	µg	mAUM	Cellulose Membrane	0.887	0.315	19
14.8	4	3	µg	mAUM	Cellulose Membrane	0.777	0.278	20
14.8	4	3	µg	mAUM	Cellulose Membrane	1.017	0.291	21
14.8	4	3	µg	mAUM	Cellulose Membrane	0.628	0.296	22
14.8	4	3	µg	mAUM	Cellulose Membrane	0.935	0.359	23

14.8	4	3	µg	mAUM	Cellulose Membrane	0.708	0.274	24
14.8	4	3	µg	mAUM	Cellulose Membrane	0.942	0.33	25
15.1	1	3	µg	mAUM	Silicone	0.809	0.333	1
15.1	1	3	µg	mAUM	Silicone	1.026	0.273	2
15.1	1	3	µg	mAUM	Silicone	0.954	0.321	3
15.1	1	3	µg	mAUM	Silicone	0.717	0.345	4
15.1	1	3	µg	mAUM	Silicone	1.13	0.269	5
15.1	1	3	µg	mAUM	Silicone	0.76	0.309	6
15.1	1	3	µg	mAUM	Silicone	0.596	0.313	7
15.1	1	3	µg	mAUM	Silicone	0.907	0.369	8
15.1	1	3	µg	mAUM	Silicone	1.395	0.337	9
15.1	1	3	µg	mAUM	Silicone	0.867	0.348	10
15.1	1	3	µg	mAUM	Silicone	1.331	0.333	11
15.1	1	3	µg	mAUM	Silicone	0.747	0.339	12
15.1	1	3	µg	mAUM	Silicone	0.89	0.319	13
15.1	1	3	µg	mAUM	Silicone	1.248	0.289	14
15.1	1	3	µg	mAUM	Silicone	0.941	0.297	15
15.1	1	3	µg	mAUM	Silicone	0.626	0.313	16
15.1	1	3	µg	mAUM	Silicone	0.743	0.296	17
15.1	1	3	µg	mAUM	Silicone	0.868	0.342	18
15.1	1	3	µg	mAUM	Silicone	1.199	0.348	19
15.1	1	3	µg	mAUM	Silicone	0.925	0.321	20
15.1	1	3	µg	mAUM	Silicone	0.85	0.363	21
15.1	1	3	µg	mAUM	Silicone	1.096	0.345	22
15.1	1	3	µg	mAUM	Silicone	0.822	0.329	23
15.1	1	3	µg	mAUM	Silicone	0.759	0.313	24
15.1	1	3	µg	mAUM	Silicone	0.826	0.304	25
15.2	2	3	µg	mAUM	Silicone	0.995	0.28	1
15.2	2	3	µg	mAUM	Silicone	0.837	0.323	2
15.2	2	3	µg	mAUM	Silicone	1.094	0.324	3
15.2	2	3	µg	mAUM	Silicone	0.899	0.33	4
15.2	2	3	µg	mAUM	Silicone	0.746	0.325	5
15.2	2	3	µg	mAUM	Silicone	0.941	0.398	6
15.2	2	3	µg	mAUM	Silicone	0.738	0.325	7
15.2	2	3	µg	mAUM	Silicone	0.675	0.327	8
15.2	2	3	µg	mAUM	Silicone	0.699	0.367	9
15.2	2	3	µg	mAUM	Silicone	0.781	0.31	10
15.2	2	3	µg	mAUM	Silicone	0.699	0.29	11
15.2	2	3	µg	mAUM	Silicone	0.625	0.268	12
15.2	2	3	µg	mAUM	Silicone	0.753	0.336	13
15.2	2	3	µg	mAUM	Silicone	0.663	0.325	14
15.2	2	3	µg	mAUM	Silicone	1.258	0.374	15
15.2	2	3	µg	mAUM	Silicone	0.894	0.291	16
15.2	2	3	µg	mAUM	Silicone	0.833	0.34	17
15.2	2	3	µg	mAUM	Silicone	0.833	0.328	18
15.2	2	3	µg	mAUM	Silicone	1.034	0.297	19
15.2	2	3	µg	mAUM	Silicone	0.713	0.362	20
15.2	2	3	µg	mAUM	Silicone	0.673	0.366	21

15.2	2	3	µg	mAUM	Silicone	1.065	0.317	22
15.2	2	3	µg	mAUM	Silicone	0.872	0.281	23
15.2	2	3	µg	mAUM	Silicone	0.781	0.285	24
15.2	2	3	µg	mAUM	Silicone	0.84	0.325	25
15.3	3	3	µg	mAUM	Silicone	0.874	0.29	1
15.3	3	3	µg	mAUM	Silicone	1.062	0.274	2
15.3	3	3	µg	mAUM	Silicone	0.754	0.289	3
15.3	3	3	µg	mAUM	Silicone	0.682	0.251	4
15.3	3	3	µg	mAUM	Silicone	0.745	0.287	5
15.3	3	3	µg	mAUM	Silicone	0.946	0.34	6
15.3	3	3	µg	mAUM	Silicone	0.745	0.33	7
15.3	3	3	µg	mAUM	Silicone	0.86	0.305	8
15.3	3	3	µg	mAUM	Silicone	0.726	0.353	9
15.3	3	3	µg	mAUM	Silicone	0.669	0.357	10
15.3	3	3	µg	mAUM	Silicone	0.73	0.345	11
15.3	3	3	µg	mAUM	Silicone	0.796	0.298	12
15.3	3	3	µg	mAUM	Silicone	1.009	0.283	13
15.3	3	3	µg	mAUM	Silicone	0.661	0.305	14
15.3	3	3	µg	mAUM	Silicone	1.191	0.317	15
15.3	3	3	µg	mAUM	Silicone	0.991	0.319	16
15.3	3	3	µg	mAUM	Silicone	0.881	0.287	17
15.3	3	3	µg	mAUM	Silicone	0.866	0.305	18
15.3	3	3	µg	mAUM	Silicone	0.81	0.323	19
15.3	3	3	µg	mAUM	Silicone	0.761	0.348	20
15.3	3	3	µg	mAUM	Silicone	0.602	0.317	21
15.3	3	3	µg	mAUM	Silicone	0.483	0.289	22
15.3	3	3	µg	mAUM	Silicone	1.047	0.308	23
15.3	3	3	µg	mAUM	Silicone	0.857	0.253	24
15.3	3	3	µg	mAUM	Silicone	0.705	0.234	25
15.4	4	3	µg	mAUM	Silicone	0.825	0.272	1
15.4	4	3	µg	mAUM	Silicone	0.822	0.34	2
15.4	4	3	µg	mAUM	Silicone	0.835	0.314	3
15.4	4	3	µg	mAUM	Silicone	1.425	0.377	4
15.4	4	3	µg	mAUM	Silicone	1.027	0.28	5
15.4	4	3	µg	mAUM	Silicone	1.024	0.247	6
15.4	4	3	µg	mAUM	Silicone	1.274	0.235	7
15.4	4	3	µg	mAUM	Silicone	0.86	0.291	8
15.4	4	3	µg	mAUM	Silicone	1.01	0.29	9
15.4	4	3	µg	mAUM	Silicone	0.66	0.365	10
15.4	4	3	µg	mAUM	Silicone	1.028	0.295	11
15.4	4	3	µg	mAUM	Silicone	1.093	0.276	12
15.4	4	3	µg	mAUM	Silicone	0.689	0.295	13
15.4	4	3	µg	mAUM	Silicone	0.58	0.266	14
15.4	4	3	µg	mAUM	Silicone	0.995	0.309	15
15.4	4	3	µg	mAUM	Silicone	0.551	0.338	16
15.4	4	3	µg	mAUM	Silicone	0.701	0.261	17
15.4	4	3	µg	mAUM	Silicone	0.68	0.235	18
15.4	4	3	µg	mAUM	Silicone	1.118	0.28	19
15.4	4	3	µg	mAUM	Silicone	1.091	0.323	20
15.4	4	3	µg	mAUM	Silicone	0.825	0.352	21

15.4	4	3	µg	mAUM	Silicone	0.737	0.314	22
15.4	4	3	µg	mAUM	Silicone	0.697	0.295	23
15.4	4	3	µg	mAUM	Silicone	0.694	0.284	24
15.4	4	3	µg	mAUM	Silicone	1.114	0.29	25
15.5	1	3	µg	mAUM	Silicone-DLIP	0.93	0.29	1
15.5	1	3	µg	mAUM	Silicone-DLIP	0.586	0.321	2
15.5	1	3	µg	mAUM	Silicone-DLIP	0.51	0.261	3
15.5	1	3	µg	mAUM	Silicone-DLIP	0.882	0.321	4
15.5	1	3	µg	mAUM	Silicone-DLIP	0.725	0.274	5
15.5	1	3	µg	mAUM	Silicone-DLIP	0.87	0.327	6
15.5	1	3	µg	mAUM	Silicone-DLIP	0.899	0.369	7
15.5	1	3	µg	mAUM	Silicone-DLIP	0.821	0.353	8
15.5	1	3	µg	mAUM	Silicone-DLIP	0.761	0.277	9
15.5	1	3	µg	mAUM	Silicone-DLIP	0.887	0.377	10
15.5	1	3	µg	mAUM	Silicone-DLIP	1.242	0.297	11
15.5	1	3	µg	mAUM	Silicone-DLIP	0.914	0.298	12
15.5	1	3	µg	mAUM	Silicone-DLIP	0.906	0.269	13
15.5	1	3	µg	mAUM	Silicone-DLIP	0.955	0.329	14
15.5	1	3	µg	mAUM	Silicone-DLIP	0.669	0.329	15
15.5	1	3	µg	mAUM	Silicone-DLIP	0.567	0.25	16
15.5	1	3	µg	mAUM	Silicone-DLIP	0.703	0.309	17
15.5	1	3	µg	mAUM	Silicone-DLIP	0.906	0.348	18
15.5	1	3	µg	mAUM	Silicone-DLIP	0.838	0.329	19
15.5	1	3	µg	mAUM	Silicone-DLIP	0.778	0.414	20
15.5	1	3	µg	mAUM	Silicone-DLIP	0.826	0.348	21
15.5	1	3	µg	mAUM	Silicone-DLIP	0.762	0.305	22
15.5	1	3	µg	mAUM	Silicone-DLIP	0.676	0.427	23
15.5	1	3	µg	mAUM	Silicone-DLIP	0.717	0.258	24
15.5	1	3	µg	mAUM	Silicone-DLIP	0.749	0.294	25
15.6	2	3	µg	mAUM	Silicone-DLIP	0.704	0.362	1
15.6	2	3	µg	mAUM	Silicone-DLIP	0.974	0.321	2
15.6	2	3	µg	mAUM	Silicone-DLIP	0.655	0.302	3
15.6	2	3	µg	mAUM	Silicone-DLIP	0.838	0.295	4
15.6	2	3	µg	mAUM	Silicone-DLIP	0.726	0.323	5
15.6	2	3	µg	mAUM	Silicone-DLIP	0.799	0.289	6
15.6	2	3	µg	mAUM	Silicone-DLIP	0.62	0.303	7
15.6	2	3	µg	mAUM	Silicone-DLIP	0.607	0.307	8
15.6	2	3	µg	mAUM	Silicone-DLIP	0.953	0.294	9
15.6	2	3	µg	mAUM	Silicone-DLIP	0.745	0.328	10
15.6	2	3	µg	mAUM	Silicone-DLIP	1.04	0.287	11
15.6	2	3	µg	mAUM	Silicone-DLIP	1.023	0.323	12
15.6	2	3	µg	mAUM	Silicone-DLIP	0.57	0.28	13
15.6	2	3	µg	mAUM	Silicone-DLIP	0.686	0.249	14
15.6	2	3	µg	mAUM	Silicone-DLIP	0.807	0.252	15
15.6	2	3	µg	mAUM	Silicone-DLIP	0.888	0.305	16
15.6	2	3	µg	mAUM	Silicone-DLIP	1.283	0.318	17
15.6	2	3	µg	mAUM	Silicone-DLIP	0.872	0.33	18
15.6	2	3	µg	mAUM	Silicone-DLIP	0.621	0.369	19
15.6	2	3	µg	mAUM	Silicone-DLIP	1.111	0.257	20
15.6	2	3	µg	mAUM	Silicone-DLIP	0.731	0.309	21

15.6	2	3	µg	mAUM	Silicone-DLIP	0.86	0.269	22
15.6	2	3	µg	mAUM	Silicone-DLIP	0.862	0.337	23
15.6	2	3	µg	mAUM	Silicone-DLIP	0.791	0.305	24
15.6	2	3	µg	mAUM	Silicone-DLIP	0.766	0.345	25
15.7	3	3	µg	mAUM	Silicone-DLIP	0.856	0.314	1
15.7	3	3	µg	mAUM	Silicone-DLIP	0.867	0.259	2
15.7	3	3	µg	mAUM	Silicone-DLIP	0.659	0.296	3
15.7	3	3	µg	mAUM	Silicone-DLIP	0.827	0.344	4
15.7	3	3	µg	mAUM	Silicone-DLIP	0.618	0.309	5
15.7	3	3	µg	mAUM	Silicone-DLIP	0.481	0.261	6
15.7	3	3	µg	mAUM	Silicone-DLIP	0.686	0.317	7
15.7	3	3	µg	mAUM	Silicone-DLIP	0.928	0.336	8
15.7	3	3	µg	mAUM	Silicone-DLIP	0.572	0.286	9
15.7	3	3	µg	mAUM	Silicone-DLIP	0.821	0.323	10
15.7	3	3	µg	mAUM	Silicone-DLIP	0.807	0.255	11
15.7	3	3	µg	mAUM	Silicone-DLIP	0.601	0.317	12
15.7	3	3	µg	mAUM	Silicone-DLIP	0.685	0.272	13
15.7	3	3	µg	mAUM	Silicone-DLIP	0.845	0.278	14
15.7	3	3	µg	mAUM	Silicone-DLIP	0.694	0.324	15
15.7	3	3	µg	mAUM	Silicone-DLIP	0.776	0.317	16
15.7	3	3	µg	mAUM	Silicone-DLIP	0.975	0.266	17
15.7	3	3	µg	mAUM	Silicone-DLIP	0.758	0.329	18
15.7	3	3	µg	mAUM	Silicone-DLIP	1.032	0.332	19
15.7	3	3	µg	mAUM	Silicone-DLIP	0.817	0.326	20
15.7	3	3	µg	mAUM	Silicone-DLIP	0.904	0.363	21
15.7	3	3	µg	mAUM	Silicone-DLIP	0.991	0.289	22
15.7	3	3	µg	mAUM	Silicone-DLIP	1.527	0.333	23
15.7	3	3	µg	mAUM	Silicone-DLIP	1.042	0.356	24
15.7	3	3	µg	mAUM	Silicone-DLIP	1.169	0.283	25
15.8	4	3	µg	mAUM	Silicone-DLIP	1.019	0.289	1
15.8	4	3	µg	mAUM	Silicone-DLIP	0.77	0.29	2
15.8	4	3	µg	mAUM	Silicone-DLIP	0.943	0.292	3
15.8	4	3	µg	mAUM	Silicone-DLIP	0.7	0.287	4
15.8	4	3	µg	mAUM	Silicone-DLIP	1.092	0.276	5
15.8	4	3	µg	mAUM	Silicone-DLIP	1.026	0.292	6
15.8	4	3	µg	mAUM	Silicone-DLIP	0.778	0.298	7
15.8	4	3	µg	mAUM	Silicone-DLIP	0.821	0.269	8
15.8	4	3	µg	mAUM	Silicone-DLIP	1.138	0.241	9
15.8	4	3	µg	mAUM	Silicone-DLIP	0.881	0.307	10
15.8	4	3	µg	mAUM	Silicone-DLIP	1.165	0.25	11
15.8	4	3	µg	mAUM	Silicone-DLIP	0.779	0.336	12
15.8	4	3	µg	mAUM	Silicone-DLIP	1.003	0.283	13
15.8	4	3	µg	mAUM	Silicone-DLIP	0.674	0.302	14
15.8	4	3	µg	mAUM	Silicone-DLIP	0.729	0.29	15
15.8	4	3	µg	mAUM	Silicone-DLIP	1.026	0.25	16
15.8	4	3	µg	mAUM	Silicone-DLIP	0.65	0.315	17
15.8	4	3	µg	mAUM	Silicone-DLIP	0.984	0.269	18
15.8	4	3	µg	mAUM	Silicone-DLIP	0.935	0.377	19
15.8	4	3	µg	mAUM	Silicone-DLIP	0.822	0.363	20
15.8	4	3	µg	mAUM	Silicone-DLIP	1.103	0.305	21

15.8	4	3	µg	mAUM	Silicone-DLIP	0.961	0.333	22
15.8	4	3	µg	mAUM	Silicone-DLIP	0.535	0.321	23
15.8	4	3	µg	mAUM	Silicone-DLIP	0.762	0.323	24
15.8	4	3	µg	mAUM	Silicone-DLIP	1.238	0.336	25
